# Supplementary material for: Healthcare professionals’ views on how palliative care should be delivered in Bhutan: A qualitative study
Source: PLOS Glob Public Health. 2022 Dec 12;2(12):e0000775. doi: 10.1371/journal.pgph.0000775 (PMC10021767; doi:10.1371/journal.pgph.0000775)
Supplement: S1 Data — (DOCX) [file pgph.0000775.s002.docx]

**Field Note for FGD with HCPs in Bumthang Hospital**

Date: 24.5.2019

Focus Group Discussion with HCP in Bumthang Hospital was conducted on 24^th^ May 2019 in the afternoon after the OPD was closed. The participants included the Chief Medical Officer, a general medical doctor, two nurses from the ward and the pharmacist. Yesterday when I was approaching the participants the pharmacist was out of station but his staff told me that he should be back today. So I contacted him through phone and he said that he is interested to participate for the discussion and that he will make it if it is in the afternoon. As we were starting with the introduction session he joined us at around 4 PM and it was good that he could make it and be a part of the discussion group.

Bumthang hospital had two *Drungtshos*, the traditional physician. However, one who could speak English was on tour and the other could neither understand nor speak English. I felt that including her in the group discussion would be inconvenient and would prolong the discussion and thought maybe I will do an in-depth interview with her. When I approached her if she would participate in the study she said that she would recommend her colleague to at least participate for the survey even if he is not able to make it for the discussion. She was not really interested to participate.

So altogether there were five participants for the focus group discussion. The discussion was conducted in the small cosy meeting hall of the hospital which already had a round table sitting arrangement and except for the noisy door while opening and closing there was no disturbance and interferences during the discussion.

All five participants were very interested and participative in the discussion. There was no one who was very dominative in the discussion or someone who talked very less although as usual the nurses talked lesser than the others and it could be to do so much with the hierarchy we have in Bhutan where doctors are considered superior than the nurses. However, I ensured that the nurses were engaged in the discussion as well. The two nurses, however, were participative and expressed their opinion whenever necessary. The discussion lasted for 56 minutes and 42 seconds. Overall the discussion was very fruitful and satisfying.

Thank You.
