# Supplementary material for: Healthcare professionals’ views on how palliative care should be delivered in Bhutan: A qualitative study
Source: PLOS Glob Public Health. 2022 Dec 12;2(12):e0000775. doi: 10.1371/journal.pgph.0000775 (PMC10021767; doi:10.1371/journal.pgph.0000775)
Supplement: S2 Data — (DOCX) [file pgph.0000775.s003.docx]

**FGD with HCPs in Bumthang Hospital on 24.5.2019**

| Participant 1 | Chief Medical Officer |
| --- | --- |
| Participant 2 | General medical doctor |
| Participant 3 | Nurse 1 |
| Participant 4 | Nurse 2 |
| Participant 5 | Pharmacist |

**May be to start with, now having read my participant information form, what motivated you to participate in this discussion?**

Chief Medical Officer:

For me I feel like this is clearly aah… that palliative care is lacking in our country. So through this study if you are going to come up with some real evidence based aah…guidelines or something like aah…which should be helpful for our population. I think that’s going to be very helpful for me as well..*smiles*..if I happen to become a patient one day…*laughs*…(doctor)

General medical doctor:

In my opinion aah…palliative care is virtually non-existent in our country and since we are seeing an increasing number of chronic illnesses like kidney disease, liver disease, other increasing number of malignancies I think it’s a very important step and I think this is one aspect aah… where patients are receiving sub-optimal care. Palliative care can help improve their lives and that of families as well.

Nurse 1:

In my opinion I think palliative care is given the least attention and after this research may be from your outcome you can, like, change something into better…

Nurse 2:

For me I felt that I will be able to know about palliative care so that I can practice for patients and be able to give them better care.

**I think all of you here today are directly or indirectly involved in taking care of patients with advanced illness, right? May be from the time of diagnosis or after being diagnosed elsewhere patients are with you for various reasons. What are some of your experiences in dealing with those patients?**

Chief medical officer:

Firstly, we do not have clear guidelines, you know, there is nothing like at which level what to give. We do not have that clear guideline. So I think like aah…whatever we see in the patients when they come diagnosed at JDWNRH (the national referral hospital) where they were given some written instructions and may be some verbal instructions to the family members. But then when they come to us we do what we have learned from the medical college and that is I think not enough. In India where I studied I think palliative care is clearly lacking in India also. So what I have done so far is just pain management only. Other than that is nothing. So what we practice right now is what we learned during those college days where we got trained. May be in Sri Lanka they have something. So otherwise there is no clear guideline or step wise you know systematic aah..dealing or systematic way for caring for the patients.

General Medical Doctor:
I think it is the same with us, having studied in Sri Lanka. There was no specific aah… since we were just doing undergraduate, I am not sure about the postgraduate, but for the undergraduates we didn’t have any specific course on palliative care and moreover, what we see here is aah…people diagnosed with terminal illness are told that they are going to die within a month or year or so. So because of that there is very poor motivation in them. So that is one factor I think that is hindering from giving proper care. There is poor motivation not only in patients but in family members as well because whatever we do they think the patients are going to die anyway. So I think aah… that’s one barrier I think that needs to be looked upon and to be thought about.

**So you mean when patients and families knows that they have a terminal illness they don’t take care and are demotivated?**

General Medical Doctor:

Yes.

**So do you think that they should not be told about their diagnosis?**

Chief medical officer:
I think usually they are not told about their diagnosis.

General Medical Doctor:

Some of them are told about the diagnosis, for example, I had seen one 70 year old man , he was diagnosed with advanced stomach cancer and he was really depressed and he said that he could neither live nor die. It is better he dies. That is what he said. And there was an old lady from *Kurjey* (nearby community), visually impaired, she had severe knee joint osteoarthritis. She was always saying aah…to give her some medication like aah…to kill her. I always advise her that this medication is going to make her feel better but instead she was asking for some medications to kill her. So because of that I think motivation level aah…I think one thing we are lacking is motivating the patients, they are poorly motivated.

Chief medical officer:
Can you repeat the question? I think we are going away…*laughs*…

**My question is, what is your experiences in dealing with patients who are diagnosed with advanced illness?**

Chief medical officer:
OK so we have seen number of such cases here. I have seen a woman with an advanced Ca Breast and the wound looked like a cauliflower, I have the picture, did you see? (*He asks his colleagues*). We also had three four cases of Ca stomach, the one from *Gangrithang* (another nearby community) you remember (*asks his colleagues*) and it was an advanced case and we had an advanced case of ALD (alcoholic liver disease). So my experience is like ...we know we feel pity on them (patients). I think if we have that capacity or if we have some knowledge in taking care of them better their quality of life could be better at least till they die. They deserve to be given you know the optimal care. But these people who have died so far, from my experience, I feel they really suffered. They really suffered out of pain, first thing is that we are not able to manage pain appropriately and adequately you know . Of course we have few options only (with drugs) but we don’t have, like you know, we are not experts in giving all these things. And that’s why I feel that we clearly lack some guideline or something. Basically we are not educated or we are not qualified enough to give such care (palliative care) for such people. So I feel like you know really feel pity and sorry and aah..…

**Helpless?**

Chief medical officer:
Ya..helpless as you said.

Nurse 1:
According to me I think they (patients) are really demotivated as Dr (participant 2) said. So I think like we have to give psychological support for them (patients). For that we should have a good counselling skills and the second one is pain management like we do this according to the doctor’s prescription. And the third one is aah… we try our best to prevent them from bedsores and like other injuries and overall I think we have to support them psychologically.

Nurse 2:
For me I feel the same thing but to support them psychologically is a big challenge and we are not able to convince them for their disease prognosis because sometimes they feel that they will feel better if they go for some other local treatments also (traditional healing practices).

**Interesting…aah… what do you do when they want to go for local treatment? Do you encourage them or do you stop them or how is it?**

Nurse 2:

For me I don’t stop them because they come with lots of history about this local treatments and so I tell them to avail it.

Chief medical officer:
I think to put it together we clearly lack the component of palliative care, the psychological component, pain management component, nursing component, then spiritual component. We do not know about all these. We lack capacity (for palliative care) . So if we don’t have the capacity how can we give the service? So I was saying like from our college days we did not get proper education or qualification and in our country there is no clear policy and guideline. So that’s why that’s what I feel.

**As a pharmacist, what is your experiences Sir? Like you know there are group of drugs that are required for patients with advanced illness, especially like opioids and all. What is your experience in making it accessible and available?**

Pharmacist:
Ok..In medicine we have classification. We have the vital medicines, essential and we have non-essential. As of vital medicines we should have them available at all times in the health centre and that is our responsibility. But the problem with keeping all the vital medicines is when we have it in the stock there won’t be patients so it won’t be used. And then suddenly it gets expired and then suddenly the need for these medicines arises. So we have this challenge.

**So did you ever come across a situation where there was a patient with an advanced illness in pain and then you didn’t have the drug that the doctor has advised or, you know, not only in this hospital but elsewhere wherever you have worked? A patient had severe pain requiring an opioid and the stock was out in your work station. Did you have such situation?**

Pharmacist:

Ever since I reached here I have not encountered anything like that but I think we will encounter. I feel we will encounter such things because like I said we should keep all vital medicines available but medicines also has expiry dates and gets expired. Then before expiry we need to mobilise. And then some medicines they are vital but they are not widely used. So we keep in limited stock and when they are required in huge amount then we have to look for mobilisation.

**Aah… Do you have morphine in this hospital?**

Pharmacist:

Yes, both morphine tablets and injections are available in Bumthang hospital.

**Ok. So it is not a problem and if you have a terminally ill patient who is on morphine 4 hourly, may be say 10 mg 4 hourly for months, are you scared that it may run out of stock or ..**

Pharmacist:

Since morphine is quite frequently used so we make sure that it is always in the stock and available whenever it is required.

**I think I will come back to the doctors and sisters (nurses) here because you are seeing patients with terminal diagnosis. What are some of the main needs in these patients and their families?**

General medical officer:
Aah… different patients have different needs. It depends on their background, some are financially weak and they need financial support. Some have social issues like they don’t have children and they don’t have anyone to look after them. So for them they need moral or psychological support. And some have severe pain. Some though terminally ill they don’t have any pain. Some though not terminally ill they might be having chronic pain syndrome. So in different people there are different needs. So in this regard I think we have to be specific with specific patient.

**What is your understanding of palliative care?**

Nurse 1:

(PC is ) Giving care to the terminally ill patients (All agreed)

**Yeah…so palliative care is giving care to patients with advanced illness to improve their quality of life and that of their families as well. Palliative care is a holistic approach and includes the physical component, like pain and other symptom management, along with the psychological, social and spiritual support. I like this, palliative care is also known as ‘care beyond cure’.**

**So now when we consider palliative care in a hospital like Bumthang hospital, what would you say on the infrastructure and manpower resources? Do you think it would require more infrastructure and more manpower? Or what comes to your mind if we are to start palliative care here?**

Cheif medical officer:
I think infrastructure wise I think we do not have problem because we have enough number of rooms where they need to be isolated or kept you know. But in terms of manpower yes, we have no dedicated staff or like who had special training on palliative care because firstly we need to assess the needs of such patient, what care they require, what are the components of palliative care, so that is what I was exactly mentioning before that we do not know about it. By definition we know what it is but we do not know the components of palliative care. So I think somebody should be given some kind of training so that we will be able to plan our management or care accordingly. Then another thing is like when it comes to services like especially aah… in terms of supplies like may be some patients would not be able to take orally and where we need to give parentral nutrition. This things are not available in our setup. So that is clearly lacking and that’s what I felt long time back. Even if they can afford to buy it is not available in the market. So I think it is still not suitable in place like ours to give palliative care. I think it should be in Thimphu aah…if not then these things should be made available through policy or something like that.

Nurse 1:
I think like if the government can open palliative care centre or hospice care because we don’t have that in our country. And as madam (researcher) said like it is a holistic approach. So if we are trained for this palliative care then we can provide good care, a holistic care.

General medical doctor:
Basically what I see here is lot of sympathy but no empathy for terminally ill patients because we have been seeing them for long time and most of us feel that they (patients) are just seeking attention, we are not considering what pain they must be going through. So I think to build that empathy training is a must I think for health care providers, training is a must. I think aah…unlike what sir (Participant 1) said I think there is a need for separate infrastructure. I think there is a need for palliative care hospital may be if possible. Just like the CKD (Chronic Kidney Disease) patients they have a *Kidu* (Wellbeing) unit in Thimphu. They have whole building dedicated for kidney patients. Just like that other terminally ill patients also need a place to stay where devoted healthcare professionals like you (researcher) can make a tour around and consult a specialist may be, palliative care specialist, as soon as possible, to fulfil their need.

**Very important point raised here by a young doctor. So as a doctor what is your opinion, will there be doctors who would be interested in specialising in palliative care?**

Chief medical officer:

Of course. This subject looks very interesting for me, very attractive for me..*smiles*…of course definitely. The first thing is I think the Ministry of Health should be convinced, the highest decision making people should be convinced.

**We are positive because our current Minister who is the founder of Bhutan Cancer Society knows what palliative care is. I met her this time and she was saying that ‘palliative care is very close to my heart’ and she was asking me like how we can start it. So one of the things I mentioned was we should think of at least one or two doctors specialising in it la. Because then we will have a team. In the team of course there can be other doctors who can prescribe but having a palliative care specialist, the outlook itself will be different. So one of our priorities is having a doctor go for palliative care specialization.**

**Now to our Pharmacist Sir, what would you say about the narcotic drug regulations in Bhutan? Is it very stringent that the opioid drugs are not easily accessible? Or is it flexible enough that patients have easy access?**

Pharmacist:

Well…our current opioid regulation or our current practice is good enough la. We need to have the documentation you know where the drug has been used and how it has been consumed. So if the documentation is there we can use as much (opioids) as you can. So it is flexible la. We have to make sure that it is not misused but if it is used for patients then it is easily accessible.

**That’s very encouraging. Thank you. Can we now discuss on the need for palliative care training**

Chief medical officer:

I think palliative care training is very important. With training many of our staff I think have potential to give very good palliative care. Given the opportunity I think many of them will be very good palliative care providers.

Pharmacist :

There needs to be someone who is taking the initiative. Otherwise as a team I think we have potential but, I think as of my understanding, to give a holistic care it involves doctors, pharmacist, nurses. As of now I think for the critically ill patients it solely involves the doctors and nurses and no one else is really involved. So there needs to be like the physiotherapists for rehabilitation purposes, then the pharmacists, the lab. So these things could also be included. So first there needs to be sensitization then everybody will take part la. So we do have potential but there has to be some sensitization and someone to take the initiative. Then the whole team will come together and there will be a multiprofessional approach.

**Very interesting. So with little introduction on palliative care today and with your strong motivation do you wish to have palliative care service started in Bumthang hospital?**

Chief medical officer:
Yes definitely (palliative care service is needed in Bumthang Hospital) . Because I am not thinking of them (patients) you know, it can be one of us one day. So that way we can give importance to the patients. Everyone has to go through this. Which means everyone needs palliative care.

**Bumthang is a very religious place and people in Bumthang are very religious which is often associated with spiritual, right? When caring for patients with advanced illnesses what are some of the challenges and enablers related to spirituality?**

General medical doctor:

Aah… I would say being spiritual first thing is people’s acceptance level is quite high (meaning spirituality enhances people's acceptance level). I think compared to, I have experienced aah…it may be my biased judgement aah..I have compared people from south (southern Bhutan), people of Bumthang and from northern part of the country. People in the south are usually very intolerant to pain I have seen. They are intolerant, have low threshold for pain and have less acceptance. Whereas here (in Bumthan, central Bhutan) I think because of their spiritual account aah…they are ok, most of them (patients) are ok with it. They are accepting and they are very glad and they are very thankful that we are at least doing something to facilitate them. So I think spirituality has a big role. May be it plays around 90% role. But at the same time there is a hindrance as well because they (patients) are mostly connected with their *Lamas* or *Gurus* (religious leaders) who tells them that injections might kill them. So because of that too much belief systems or too much faith or too much beliefs in the *Gurus* can also hinder our treatment. (Need to train the religious leaders in PC)

**What is your experience as a nurse, when you have a dying patient in the ward how does spirituality affect the scenario? Any experiences sister (Nurse 1)?**

(There wasn’t anything from Nurse 1)

Chief medical officer:

The recent one. Can you (nurse 1) tell about the recent one? An old man who was oxygen dependent with COPD patient from Tang. He (patient) once told me (doctor) that he wanted to go home to die. He said he didn’t want to die in the hospital as he knew that he was going to die soon..*laughs.*.

**So did he go back home?**

Chief medical officer:

Ya, he went home. I think he must be living still

Nurse 1:

Yes, he is still living

Chief medical officer:

But then on and off and on and off in a very bad condition. I really feel pity on him. He is a case of COPD, very bad. Quite often without oxygen his oxygen level will drop to below 60s and even with that oxygen supplementation he cannot sleep. That’s there you know he cannot sleep. Literally he is really suffering. He tries to sleep and poor him his brain demands sleep and as soon as he sleeps he loses that posture control with the sleep and then he goes into hypoxia. It is so pathetic.

**Actually, one of the participants today would be *Drungtsho*, our Traditional Physician. In other hospitals so far that I have been the Drungtshos were well versed in English. So there wasn’t any problem. Aum Drungtsho (Addressing an senior lady Drungtsho)could not participate today because she cannot understand and speak English. So I slightly changed my strategy and I am thinking to do an in-depth interview with her if it is feasible.**

**Our patients with advanced illness also look for other options, right? And Traditional medicine, a part of our health system, is one option that people look for. How do you see a role of Drungtsho or Traditional Medicine in palliative care in Bhutan?**

Chief medical officer:

Ya, they (Drungtsho, traditional physicians) will have a bigger impact in PC

Pharmacist:

As you pointed out the importance of spirituality, may be they (Drungtsho, traditional physicians)

can play that (spiritual) role in healing patients through spirituality (pharmacist)

General medical doctor:

Plus most of the medications, that formulation of traditional medicines is mostly the herbs and plants. So people have more faith and trust. One thing from my experience, what I have seen is people having more trust get better soon. But people who do not trust, who are sceptic about treatment or who are not willing to get treated, they take some time aah… may be to heal or something. So people have trust in traditional medication. So I think it will play a huge role in palliative care.

Pharmacist:

And like for those patients, as you (researcher) have pointed out, for those patients who do not have treatment I think traditional medicine is an option.

**I understood that Traditional Medicine in Thimphu is starting with all these healing practices like they already have in place steam therapy and all but they are also starting with meditation and yoga for patients which was quite interesting.**

General medical doctor:

Aah… regarding yoga I want to tell one of my experiences. I was participating in one of the yoga classes in south India. There was one man about 70 years old. He said that he was diagnosed with liver cancer. Aah…his alpha fetoprotein was in thousands which means he was having advanced liver cancer. After may be three to four months of doing some yoga practices it (alpha fetoprotein) came down to aah… in single digit. I am not sure whatever information he was giving was 100% true or not but according to him he was trying to convey that yoga was able to reverse his liver, his total liver damage. So we are not sure, it is not proven yet but it has a great role in boosting the morale and might also cure some of the illnesses may be.

**There are so many literature on these different healing practices that have helped patients improve their quality of life.**

**Now what do you all say about the awareness on palliative care? How aware are our health care fraternity and the general public?**

Participant 2:

The awareness (on PC) is very poor. Especially in our community it is very poor. As I already mentioned that most of us (healthcare professionals) consider people with terminal illness are attention seekers because for an acute case we can just check his pulse and see that his pulse is high, BP is elevated, he is sweating but in chronic patient dealing with chronic pain or chronic illness aah…the body is used to it, acclimatized, so you don’t see the change in pulse, breathing is fine, BP is fine so we underestimate the pain. So the overall knowledge and awareness (on PC) is very poor I think in the health staff and forget about the public even among the health staff it is very poor. (Indicates high need for PC education and training)

Chief medical officer:

I think you (generag doctor) are definitely right but I think it is wrong to say as attention seeker because is it ok to say that? How do you say they are attention seeker? I never thought that way so far. When the patient is diagnosed with terminal illness if we (doctors) feel that we are just waiting for the death and that there is no other better options that means we clearly lack knowledge. So I think there is lack of awareness among the health workers as well the public and I think we need to create more awareness.

**We have two sisters (nurses) here from the ward, who spend about 90% of your time with the patients, right? You have seen the most of patients’ suffering, right? You will be seeing more and at this time you are learning something about palliative care. How do you feel about it?**

Nurse 1:

I feel that we have to give more care to them like the psychological support and all because the first thing we have do is the psychological aspect I feel then only the medications will work. Otherwise I don’t think giving too much of opioids when patient is not happy and if he cannot digest the diagnosis then it will be difficult for him to live or cope with the remaining days. I feel that more than giving the opioid I think we have to give a good counselling because we just can’t go there and talk about the diagnosis and the pain and all but rather we should give a good counselling like how to cope up. So good counselling would be a good attention. (Indicates high need for PC education and training on developing communication skills)

**Do you feel, as a nurse, that you have more opportunity to help such patients?**

Nurse 1:
Yes, yes, (as nurses) we definitely have more opportunity to make a difference. We can talk to them, listen to them, we can share our stories like some of us might have experienced through past patients and we can share with them, listen to what they are going through, just don’t ignore what patient is going to tell you.

**Very important point, listening. Sometimes I think the health worker just listening to the patient is so much healing to the patient.**

General medical doctor:

I want to add to that if I am allowed to.

**Yes please, of course you are.**

General medical doctor:
These days we have seen lots of changes in the health system. People often mention that health workers are very friendly and I think that is one most important thing. One important thing is not just prescribing and giving advises and counselling, the most important thing is, as already mentioned, listening. So how can we do that? If we are not friendly with the patient they won’t be able to open up with their problems, they will hide most of the things, 90% of the things they will hide, they will just say, fake a smile and they will not be able to open up. So being friendly with them, creating a friendly environment, not just giving counselling but making or giving them options like this , this and this asking which option he/she wants. Throwing some details about the information, for example, if there is a surgery what is the chance of getting cured and if there is a medication what is the chance of getting cured. So if we give them open options we create a friendlier environment which I think we are inculcating in our health system. Previously this was a different case but now I think people (HCPs) are becoming friendlier and patients are able to open up and I think this is a progress, a great progress, and we can go a long way from here. That’s my personal opinion. (Enabler for provision of PC)

**That’s very encouraging? Is there anything else that you wanted to discuss and we haven’t covered yet? Is there anything that you want to ask?**

Pharmacist:

I have heard of this term (palliative care) but I didn’t really know what it exactly was. After listening and being part of this discussion I felt that it is very important. As you said it is care beyond cure I think it really is. Recently one of our HA’s (Health Assistant) child got sick and was critically ill and that time I was not here but most of our doctors and even most of our health workers were here giving support (to the HA’s family) . The doctors and nurses were taking care of the child and other health workers were , you know, they were taking care of the mother, the HA, giving her psychological and emotional support. So this is one good example of palliative care. And ya, like that if we provide to all critically ill patients it would be so helpful.

General medical doctor:
Not just to the patients but to the family as well.

Pharmacist:

Ya. That time I think some of the health workers did not sleep the whole night giving her (the HA) support and the next day the child was airlifted to the national referral hospital.

**What happened to the child, very interesting?**

General medical doctor:
She is doing well now. She was in coma for about a week but now she is out of it. She is undergoing physiotherapy and is in the paediatric ward and is able to walk. They are just keeping her because of the deranged liver function but now she is doing very well.

**So that was a short term suffering with lots of support, lots of help and then the cure aspect was good. Imagine a patient who is there for years with an advanced cancer with so much of pain, so much of social stigma and discrimination and other social factors, so much of spiritual distress. How much our support and care would make a difference, right? That’s what literally motivates me and I know many will be motivated once they really know what palliative care really is.**

**Is there anything else, any burning issue there?**

(Nothing)

**So you are all aware that this is a PhD project on developing a suitable palliative care model for Bhutan and the objective is to integrate palliative care into the Bhutanese health Care system. Do you have any advice or a comment or a suggestion to me? Because you all are the ground reality, you are seeing such patients everyday. How do you feel or what would you advise me regarding this project?**

Chief medical officer:
May be some of you can comment but for me it is very clear. Most of the time like after studies lot of theoretical aspects comes but I would like to suggest that you come up with something realistic, doable after your study gets completed, simple and uncomplicated one. That is what my expectation is from you. It is of course mentioned here that it will be spiritually, culturally and socially applicable and it should be realistic.

**Thank you so much Sir . Well, the emphasis of the project is to make it locally appropriate and I am hoping that we can do it.**

Pharmacist:
Aah… since it is your plan to integrate this palliative care into the health care system so you have your long term goals to train the doctors. And as short term goal my suggestion would be to sensitize the health care workers, train someone to initiate but then again if you try to appoint someone as a focal person don’t go for someone who is already involved or those who are already very busy. Like if you appoint a doctor I don’t think they will be able to initiate because they are very busy. Aah.. you should compare the work load among the health care workers. There are some whose work load is quite lighter than others so maybe you could target those health workers train them and as a short term solution you can train them and let them start this. So let them form the team and they will take care of everything.

**That’s a good suggestion. Thank you Sir.**

General medical doctor:
Aah.. I think Kipchu sir (pharmacist) has spoken our heart out. I said there are people who are very busy and they may not be very good advocates for palliative care. We try our best but even then we get busy with other things but there are people who are bit free, not that they don’t do work, but they are bit free so they can be good advocates. Other thing is we can also, you know there are animations that they present on news channels like BBS (Bhutan Broadcasting Service). So we can make animations of palliative care as well. Mostly what they are doing these days is regarding STIs (Sexually Transmitted Infections), acute illnesses, NCDs like diabetes and hypertension. Like that we can also do on palliative care, chronic illnesses. I think that would also motivate people to get into palliative care.

**Very good suggestion for public awareness. News channels and social media are important platforms to advocate PC to the public. Thank you for the suggestion Sir**

**Anything from my nursing colleagues here, any suggestion?**

Nurse 1:
Aah…training is very important for us, first for the staff. Then only come up with sensitizing the public. (nurse) Once we have facilities then we can provide to the public.

**Right. This has been a very fruitful discussion. Thank you very much. I will take all the points, use it as a data for my project and do my best. Thank you very much.**

Chief medical officer:
All the best for the future.

**Thank you very much Sir.**
