# Supplementary material for: Healthcare professionals’ views on how palliative care should be delivered in Bhutan: A qualitative study
Source: PLOS Glob Public Health. 2022 Dec 12;2(12):e0000775. doi: 10.1371/journal.pgph.0000775 (PMC10021767; doi:10.1371/journal.pgph.0000775)
Supplement: S3 Data — (DOCX) [file pgph.0000775.s004.docx]

**Field note on the interview with HCP in Dagana BHU I**

I came to Dagana BHU yesterday and came to know that the only doctor in the BHU is away on medical leave. The BHU had four nurses and usually one nurse is posted for each shift, morning, evening and night. The BHU did not have pharmacist, physiotherapist and even Drungtsho. Although the participant information form was also given to two other nurses that I met yesterday and a Health Assistant only one nurse and the HA were willing to participate. Since the HA said she will be very busy in the Mother and Child Health clinic till late in the afternoon and so was not really keen to participate in the interview. A nurse who was posted for the morning shift was interested to participate in both survey as well as the interview. And luckily there was no patient admitted in the ward which made me feel comfortable because the only nurse on duty could fully be engaged in the interview and we didn’t have to worry about the care of patient that could be compromised.

The interview was conducted in the nurses’ duty room and there was not disturbance during the interview. The participant seemed to be very interested in the topic and he did have lots of experiences of having been involved in taking care of patients with life threatening and terminal illnesses. After completing the interview when I met other categories of health workers they told me that this brother, the participant whom I interviewed, is one of the best nurses there in Dagana BHU. I found him a smart professional and interested in palliative care. He was very happy that he got this opportunity was he kept repeating how much he learned from the interview and even from the survey questionnaire.

Thank you

Date 18. 6. 2019
