# Supplementary material for: Healthcare professionals’ views on how palliative care should be delivered in Bhutan: A qualitative study
Source: PLOS Glob Public Health. 2022 Dec 12;2(12):e0000775. doi: 10.1371/journal.pgph.0000775 (PMC10021767; doi:10.1371/journal.pgph.0000775)
Supplement: S4 Data — (DOCX) [file pgph.0000775.s005.docx]

**Transcript of interview with HCP in Dagana BHU I on 18.6.2019**

This is an in-depth interview as it was not possible to include other health workers. The only doctor was away, there was no pharmacist and physiotherapist and this participant was the only nurse on duty.

**Thank you Karma for willing to be part of this study. To start with the interview can you please share some of your experiences in taking care of patients with advanced illness in this hospital or any other hospital that you have worked so far.**

Thank you madam. Aah… palliative care in Bhutan is in fact very premature I would like to say. As per my experience palliative care need more attention from the doctors, nurses and from the patient’s attendants. Palliative care should include personal hygiene like mouth care, hygiene, bed bathing and feeding, assessing feeding. As per my experience the patient requiring palliative care are stroke patients, cancer patients, then severely disabled patients like blindness, then paraplegic patients. Then also those patients who are admitted in the hospital for a long time like leukaemia patient, then fractured patients and patients with diabetes, especially with diabetic foot, those with hypertensive neuropathy. So all these cases needs palliative care.

**Did you attend any kind of training on palliative care so far?**

Till now I have not attended any workshop or seminar on palliative care.

**Do you have any idea what palliative care exactly is?**

Yes la, palliative care means providing care till the last breath of the patient which includes medical, nursing and physiotherapy and also daily, the day to day life personal care.

**And also the psychological**

And emotional needs

**Ya, emotional needs and the spiritual needs of the patients because when the patient is diagnosed with a life-threatening or life-limiting illness which means there is no prospect of cure a patient also goes through lots of emotional, social, psychological and spiritual distress along with the physical pain and distress. So palliative care is a holistic approach of care to improve the quality of life of patients and the family members all together.**

**What is your experience in taking care of patients with terminal illness and their family members?**

Aah… we had one patient admitted in the BHU one year ago. Aah… he was admitted as a stroke with paralysis and we had to provide all the personal hygiene. Then the basic needs which includes aah…fooding, then the urinary care, then all those day to day care. Then we had to also provide the dressing for the wound (bed sore) which developed during the stay in the hospital

**Was it a bed sore?**

Yes, bed sore. Earlier I think it was only a second degree pressure sore but later it became a third or even fourth stage and the patient stayed here for more than four months. We have done with all the nursing care, changing of position two hourly, then propped up position, then dressing but ultimately it didn’t work (*smiles*). We had to, even we had given, with the advice form the doctor, antibiotics and all these things but it didn’t work.

**So when you say it didn’t work you mean the wound didn’t get better?**

Ya, it didn’t get better. So we could even see the sacrum, then the bony part of the knee.

**So he had bedsores all over?**

Yes, yes, the whole body.

**Aah… what were some of the challenges while caring for this patient?**

Aah… here in Daga BHU we have got an acute shortage of nurses. I have to look after the patients in the ward and then do dressing in the MOT (Minor Operation Theatre). Then again I have to go to delivery room to conduct delivery. The patient workload is very less, of course, but the quality, the quality of care I think we have not provided to him (to the stroke patient). But we have done daily dressing with two drums of gauze, sterilized gauze (*smiles*) and we have provided everything, we have done everything but I think it is high time to have palliative care in Bhutan. That is my opinion.

**How did you feel when you could not provide adequate care to this patient? Do you think there is lack of palliative care knowledge among doctors and nurses?**

Yes la. Doctors, nurses and all health care providers definitely lack knowledge on palliative care. It will be more beneficial if the Ministry of Health provide workshops, seminar or short term course in palliative care for us. And even a training on TOT (Training of Trainers) if they give us it will be more beneficial. We can provide better and holistic care to the patients.

**OK. So besides the wound care, the bed sore wound in that patient, what were other needs in the patient when he didn’t have the prospect of cure? You said he was a stroke patient with paralysis, did you also see other needs in that patient besides the care for his bed sore?**

Yes la, he (the stroke patient) had spiritual needs, then he needed nutritional support, balanced diet, then he also needed emotional support, all these things. We had to always assure him that ‘ok , you will be alright,’ Then we had to provide that emotional support and all these things. But ultimately patient got into depression. He became depressed of the long term illness. So we had to even give counselling. Then again we had to repeatedly give emotional support and we had also requested the doctor to give some medicines to improve his emotions.

**Besides the doctors and nurses, were there others to provide psychological or spiritual support to that patient?**

Yes, one *Lama* (religious leader) visited and gave him a spiritual teaching and *wang* (blessing).

**Do you think that it helped him?**

Yes, I think for one week he became normal, his emotions were normal but again after one week his emotions were up and down (*laughs*). Emotional swing was there.

**So in such patients they require continuous counselling and support and from spiritual persons as well, right?**

Yes, definitely la.

**From your experience in taking care of such patient what are some of the changes you would like to see in the health system so that you are able to provide a better care. From our current policies, you know, what are some of the changes that you would like to see?**

Aah… In Bhutan we need nurse prescribers like in the other countries so that we can give opioid analgesics to bring down the pain and suffering of the patient because nobody wants pain right? We ourselves do not want pain and we want to give a peaceful death to palliative patient, I mean palliative care patients, we want to give a peaceful death. So we need nurse prescribers in Bhutan to break down the problem. Then we also need support from the patient attendants, then the local leaders, relatives, and we want to give sensitization on palliative care to them also. The Ministry of Health should give trainings and seminars on palliative care for the health workers and doctors so that we can give quality care to the palliative care patients.

**Interesting. What are some of the analgesics that are available in this hospital, It is a Grade I BHU right?**

Yes, we have almost all the opioid analgesics.

**Like?**

Like morphine, pethidine, then codeine phosphate, then we also have tramadol. We have all those medicines. (Opioids are available in BHU I)

**So you have no problems with drugs required for patients who has severe pain, right?**

No, but we lack knowledge in using them (opioid analgesics) and the right to prescribe

**OK. Interesting. You also mentioned about having less staff here, right?**

Yes, we just have four nurses, one doctor and at the moment even he is on medical leave. I am the only nurse for the morning shift and there are three other nurses on station for the other shifts.

**How about the infrastructure? If you are to provide palliative care, do you think, the current infrastructure would be sufficient or you think that you would need more?**

Yes, definitely we need a right infrastructure to provide better quality palliative care. Like we need more air mattress to prevent pressure sore. We need a proper hospital bed, one we can raise the head end and the foot end. Then we also need well heated rooms to prevent hypothermia in winter and ACs to prevent too much heat in the summer. Then we also need some diagnostic facilities to prevent more complications.

**Like?**

Like aah…machine like a photometer to see cholesterol and all these levels. Then we also need ultrasound to prevent more complications of the palliative patients. We also need X-ray, all these facilities to prevent complications and enhance the life of aah….patients who needs palliative care

**So at the moment, do you refer the patients to Dagapela hospital (district hospital) if they are in need of ultrasound, X-ray, is that right?**

Yes. We do not have ultrasound at the moment but in the twelfth five year plan it is coming. The unit is under construction as you can see there (*shows the construction site that can be seen through the window of the nurses’ duty station where we had the interview*)

**I see. So the infrastructure is coming up which is very good.**

So that is for X-ray and ultrasound. At the moment we refer patients to Dagapela hospital and again we bring them back to Daga BHU (*Laughs*) after availing the diagnostic facilities there.

**So you mentioned that you don’t have issues with morphine stock, right?**

Yes

**You have adequate morphine stock which is very good. So I am understanding that to provide a quality care to the patients who are terminally ill and are dying you would like to have change in the system where a nurse is allowed to prescribe. And additional staff so that you have more people to provide palliative care. You also need air mattress, hospital beds right? Anything else that you would think of when it comes to taking care of patients with terminal disease from your experience?**

We need sympathetic care givers with empathy, sympathy, all these things

**And compassion**

Compassion to provide better care. If we don’t have these empathy, sympathy then we will definitely lack the care. Even if we have huge knowledge, if we lack empathy, sympathy and compassion from inside our heart then we will lack this one (he meant ability for PC) but till now we have been providing with all these (*laughs*) components to all the patients for palliative care.

**Since you brought up the topic of empathy and compassion in taking care of patients, how is your experience when it comes to taking care of patients who are very, very ill, very frail, nobody to look after, so much in pain, and are dying? How does empathy and compassion help you provide care to such patients?**

Yes, empathy and compassion helps in providing better care like we see, being Bhutanese we are more compassionate I think

**Being Buddhist you mean?**

Yes, being Buddhist we have more compassion and with compassion we can definitely provide better care like we say in Buddhism caring for a patient is like caring our mother, you know, we say like this. All sentient beings are our previous mothers so we would develop better energy and give better quality of care. (Role of Buddhism in caring)

**And when you understand that the other person is exactly like your mother you have that drive from inside to take care of that other person irrespective of whoever he or she is, right.**

Yes

**Amazing. And I wish all the nurses have that understanding and determination to provide care.**

Definitely we should have a bonding between patient and care provider. So when you have a strong bond between the patient and the care provider they will ask this doctor, this nurse should come and give care to them. So when they say, for example, some patients they call me at midnight also. ‘Please come and help me’. So I come even at 1 AM also and provide care like provision of pain killer, then changing of soaked dressing and I do all these things without hesitating and I give them counselling as well. For example, patients often call doctor and sometimes they call me. Patients call us at midnight also and even on holidays. When we give them better care patients develop trust on us. Most important in providing health care is gaining of trust between the patient and healthcare provider. When they trust even if we give a simple touch therapy they have a full confidence and hope that we will do right things and they are receiving the right medicines and right treatment from the health centre. So I think gaining trust is the most important for palliative care provider.

**Interesting. What is your experience dealing with patient’s family members? When you have a chronically ill patient it is not only the patient who is suffering but also the family members, right?**

Aah… for chronic patients and patients needing palliative care patient attendants (patient's family member) at the most they look after them only for three weeks. They give full care including personal care. After three weeks to one month then they lose hope, they give up and they leave. So our responsibility is that we give counselling to patient’s families also. So we have to encourage them (*laughs*)

**How is their response?**

Some of them genuinely look after the patients but some of them get frustrated but counselling does help. Some family members say that even after being in hospital for three months the patient is still not getting better and instead is getting worse. But when we counsel and give our best to care for the patient the attendants are convinced and they cooperate as well.

**Has there been issues where the family members didn’t want the patient to know the diagnosis and continued to care?**

Yes. There was one patient. I think a case of brain tumour. The patient didn’t know (about the diagnosis) and the attendant wanted to hide it from the patient.

**Was it an advanced tumour?**

Advanced, 4th stage brain tumour. So the attendant didn’t want to let the patient know.

**How old was the patient?**

78 years old male patient

**How was the experience?**

We have kept him I think for more than a month here. Then after one month the patient’s attendants knew from the doctor who explained that it is a terminal case, 4th stage brain tumour, so he was kept here for one month and then the patient had requested for discharge from hospital. They took him home and patient expired on the day of discharge. He died without knowing what he was suffering from (what his diagnoses was)

**How did you feel about it as a care provider?**

As a care provider I think it is not right because patient himself should prepare for the death. I think it is not right to not let him know about his disease.

**Why do you think the patient’s families did not want the patient to know about the diagnosis of a terminal illness? What do you think is the main reason behind?**

I think to prevent the patient from depression. If you let him know his disease he will have panic and worry and all these things and finally will go into depression which may lead to suicidal attempts, you know? So I think the patient’s attendants did not want to tell the diagnosis.

**So do you think this should change or do you feel that this should continue and how can we change this?**

I think we need to change the system and I think we need counsellor in the BHU so that patient diagnosis has been known and the counsellor will give more client centred counselling to the patients and prepare for the better life. (Need for counsellor)

**Do you have anything else to share besides what we have discussed? About your experiences on palliative care or anything else that we didn’t discuss and you feel we should have discussed?**

Yes. Patients also need some financial support to fulfil their spiritual need. They say that they need to perform *pujas* (rituals) to get better but they won’t have money. When their no financial help then their spiritual need is compromised.

**From your experience working in this hospital, in this community what is your experience on the public’s perception on death and dying? How is death and dying taken here culturally?**

Death and dying in Buddhist community is respected and in fact they say that a patient, a chronic patient who needs palliative care they say that if they die it is ok because they have suffered a lot and they have even spent a lot of resources. So they say that it is ok for them (*laughs*)

**Is that what you have understood from the public here?**

Yes

**From your experience, and from your beliefs and values, when there is a patient with a terminal illness how do you regard him/her as a dignified individual? How can we assure a dignified care, a dignified death, that dignified transition? How can we provide that?**

Dignified care means that the patient has received adequate care. Adequate care like both from medical, then nursing, which was received adequately. We do not want to, for example, a patient with stroke, we do not want him to die of pressure sore. And paraplegic patient we do not want him to die of pneumonia, simple disease which can be cured by antibiotics. We do not want death to happen with simple diseases. We want to give everything we have in our facility and if he needs referral we would refer him. When a patient dies after fulfilling his wishes maybe we can call it a peaceful death or as a dignified death.

**Till now have you experienced providing a dignified death to a patient or do you regret not being able to provide a dignified death? Any instances?**

Yes la. We have so many instances where patients have died with simple diseases like a pressure sore and with septicaemia, then with, recently a patient has died, a stroke patient died due to pneumonia. We had given antibiotics but it was too late. Patient was brought from home in a gasping state. We gave him ampicillin, gentamycin, oxygen and everything but it was too late. After X-ray we found that his whole lung was collapsed. So yes la we have so many problems.

**Interesting. Now, you have understood that this project is a PhD project and the objective of the project is to develop a suitable palliative care model for Bhutan. From whatever little information you have read from the information form and from the survey questionnaire, do you have any specific advice or suggestion or comments. Because you are a ground reality, you are an experienced health worker here, any specific advice or comment.**

Palliative care is very immature in Bhutan but after doing the survey and the interview I got good knowledge which is very helpful. Palliative care in Bhutan is very urgent now, badly needed and it will be more required in future. Just now we are lacking everything when it comes to palliative care I hope madam’s project will improve the situation. I wish you good luck madam.

**Thank you very much.**

**I am hoping that this project will introduce palliative care into the Bhutanese health care system and I am happy that I could interview you. Thank you very much. The information and the data that you have provided will be very helpful for the project and I appreciate your participation. Thank you**

Thank you madam
