# Supplementary material for: Healthcare professionals’ views on how palliative care should be delivered in Bhutan: A qualitative study
Source: PLOS Glob Public Health. 2022 Dec 12;2(12):e0000775. doi: 10.1371/journal.pgph.0000775 (PMC10021767; doi:10.1371/journal.pgph.0000775)
Supplement: S5 Data — (DOCX) [file pgph.0000775.s006.docx]

**Field note for FGD with Drungtshos, Department of Traditional Medicine, Thimphu**

Date: 12.7.2019

Venue: Conference Hall, Traditional Hospital

This focus group was planned on 9^th^ July when I came to approach the Drungtshos for participation in the research. While six Drungtshos consented to participate in the survey four were further interested to participate in the focus group discussion. All four were very young enthusiastic traditional physicians who became very interested in the concept of palliative care.

The discussion was conducted this afternoon in the conference hall of the traditional hospital following some tea, coffee and snacks organised for the participants. One of the Drungtshos was senior to the other three and she had just recently completed her Masters Degree and she was also much more experienced and so she talked a bit more than the rest but that didn’t make the rest bored or deprived of opportunity. The three young Drungtshos respected her opinion and had so much of regard for what she was saying actually.

This discussion with the traditional physicians helped me understand better the potential of traditional medicine in palliative care for Bhutan. It helped clarify many of my own doubts and ignorance about traditional medicine. These young Dungtshos were all very positive about the project and were so motivated to be a part of palliative care team in future. I realised having a Drungtsho in the palliative care team would make so much of difference in the quality of life of our patients in Bhutan and their families. The discussion went on for one hour twenty eight minutes and nineteen seconds and the language used was a mix of English and Dzongkha.

I am very happy that it was one of my supervisors, Professor Claire Johnson, who suggested to do a separate focus group with the Drungtshos only and that was a very important suggestion for which I am very thankful.

Thank you
