# Supplementary material for: Healthcare professionals’ views on how palliative care should be delivered in Bhutan: A qualitative study
Source: PLOS Glob Public Health. 2022 Dec 12;2(12):e0000775. doi: 10.1371/journal.pgph.0000775 (PMC10021767; doi:10.1371/journal.pgph.0000775)
Supplement: S6 Data. Transcript — (DOCX) [file pgph.0000775.s007.docx]

**Transcript of FGD with Drungtshos (Traditional Physicians), Traditional Hospital, Thimphu on 12.7.2019**

| Participants |
| --- |
| Drungtsho 1 |
| Drungtsho 2 |
| Drungtsho 3 |
| Drungtsho 4 |

**Kuzuzangpo la (*Bhutanese way of greeting*)**

**To start with the discussion, because you have read my participant information form, the first thing I would like to hear from you is what made you all feel like to participate in this discussion. What is your motivation to participate in this focus group?**

Drungtsho 1:

Las (ok) Kadrinche la (Thank you). Actually for me when there is something to do with research I never deny. Even before I got my Master’s Degree I was always very interested in research although I didn’t really know how research is carried out and I often used to be confused. Now that I have completed my Master’s Degree I feel I am on track with understanding about research. And after completing my Master’s this is the first time I am participating in the research and I got very interested on your project because this is going to be very useful in the future for those *Nyam thapai gi* *nape* (patients who do not have cure). Today if we deny to participate, with the fear of wasting time, when someone has initiated such a project and we, being the relevant people to be involved, from the Traditional Medicine side, if we do not participate and if we do not get involved then later we will not really be able to help them those patients who do not have cure. So that’s why I became very interested in participating. I am already interested in such work from before and now I am even more interested. This topic is really of interest to me.

**Thank you very much.**

Drungtsho 4:

I understood that your research is about helping patients more than ever before when they are at their last stage and I felt that I can learn how to deal with such patients better. So that is why I was interested to participate.

**Thank you**

Drungtsho 3:

So the main reason why I got interested to participate in your focus group is just recently I faced a situation where a lady was diagnosed with stomach cancer and the family was told that she will not survive for more than six months. The patient was in so much of pain in the abdomen and her back at the sides. It was intolerable pain and their last option was us because the lady had a hope that Traditional Medicine may help her. So she met our specialist (The Traditional specialist) here and she was admitted here (at the Traditional Hospital) and she was taken care by some of us. What I have been feeling is that our treatment and our therapies here are not really going to help her so much and I have not come across such serious patient in the past, I have not experienced. So I didn’t know how to deal with her, what advice to give, how to counsel. Because in this situation the patient did not know that she was actually in her last stage, she did not even know that she had cancer. She was referred to India and was brought back as terminal case. Her two attendants came to me and told me about it. And they just wished if we had something to do for her. That time I was dumb you know. I didn’t know how to approach. I did not know how to counsel the patient because she didn’t know about the terminal nature of her illness. Just talking to the attendants was not very useful. So in such situation I wished if we were aware about how to approach the situation tactfully, what type of counselling to give. At least if we knew about it we could help a bit more I felt. So with your research project I am sure something will come out of it and we will also be benefited. There is an opportunity that we will be able to help such patients in future. So that is why I got interested to participate in your group discussion.

**Thank you**

Drungtsho 2:

For me the main reason that I am participating in your research is because although we are all there to help patients and we do try our best to do whatever we can to make our patients as happy as possible. However, when it comes to terminal cases we have been doing what we can but we do not know anything about palliative care, how it is delivered. When madam (researcher) is here with such an idea to introduce something which is very important and exploring through research I naturally felt that I want to participate and that I will also get to express what I have been experiencing and that’s why I am here to participate in the discussion.

**Thank you very much. It is very encouraging listening to your motivation.**

**Now the second point of discussion is, what are your experiences in managing patients with advanced illness like advanced cancers, heart failure, lung failure or any diseases which doesn’t really have a prospect of cure? Like what type of patients usually come to you? How do you manage and what are your experiences? What challenges do you face?**

Drungtsho 1:

If I have to say about my experience I think I have come across not more than two to three such patients with advanced illness. One was a case of a liver failure due to chronic alcohol intoxication. The other was stomach cancer. I think I came across those two only. For such patients from our traditional side we have two ways to approach. Our specialty is, even if the patients are told that they do not have cure from the hospital, from the allopathy side, our unique approach in traditional medicine is, among the 404 different types of diseases that are there, we categorise 104 as *Karmic* disease, *tshe ngen gi lay* (due to past karma), so all these cancers and other chronic diseases are all because of one’s past *Karma* (principle of cause and effect). Because of one’s past karma today one has to die before his/her life expectancy is completed, one has to fall ill before it is time to really become ill. So this is the reason. So what we know is if our past karma is purified the disease can be cured. And we do have people who are told that they will die in six months but are living for years and years. So that may sound to the allopathic practitioners as a mere coincidence or they may have other scientific and technological justifications. But from our side if the past karma is purified the disease naturally goes away. So to our patients we first emphasize on *tsho sa gop* (accumulating merit), then asking them to have faith and take refuge in the *Kencho Sum* (the three jewels, Buddha, Dharma and Sangha) and we ask them to recite *Sangay Menlha’s* (Buddha of Medicine) mantra. They will not be able to prostrate but we ask them to make offerings and to rely on the Three Jewels more than ever before. So that’s how our approach is for such patients. If I give an example, you may be aware of Lopen (Sir) Kinley Gyeltshen. Do you know? He is from Punakha.

***Shook head indicating that I do not know.***

Drungtsho 1:

He was the head of the, what you say, the University, the *Lekshey Jungney Shedra* (University of Buddhist Studies) in Punakha. He was given a guarantee of six months from the hospital for liver failure. So what Lopen did was, he had never done meditational retreat in the past, he was mostly teaching in the universities. So when he knew that he is left with just six months he started to meditate. When he initially started with mindfulness meditation what he did was he planted a peach seed in the ground. And he said that he will continue to meditate on mindfulness till the time when the peach tree gives flowers. And he continued to meditate and he lived for six years. It was so amazing. And when he finally died there were so many unnatural signs shown. From the ashes, after his cremation, it is said that there were *Ringsel* (relics) found. There were relics of five different colours that was found. So this is very unique from the context of Buddha Dharma. So that’s why some even say they are cured when they are told that they have cancer. If we have to categorise the terminal illness we say it as *Zhiwa ngen gi lay*, karmic diseases, hundred and one karmic diseases, so the terminal diseases falls under this category. One thing is we say that one needs to accumulate merit and do positive actions. The other thing is, once it is terminal they have pain, right? So we need to focus on managing pain even if there is no cure. When we think of pain management and if their Hb (haemoglobin) level is normal then for the liver disease there are so many points, bloodletting points. So when we release the impure blood they will feel relieved from pain. Then if the pain is at certain body parts we do cupping to release those pains. And if the patient is anaemic we do have dry cupping therapy.

**Without letting the blood flow out?**

Drungtsho 1:

Yes. So it is said that the disease is brought out from the pores of the skin as steam. And the pain will be reduced. Then if it is like the whole body pain we have steam bath, body massage. So we have so many ways in the traditional medicine when it comes to pain management. Now challenges are as one of my colleagues mentioned earlier. It is always a difficult situation when a patient is told that there is no cure but I want to say that we do continue to give hope to the patients. How can I say that ‘you are going to die’? I tell them ‘you will get well’. Actually the patient would have already known that he/she is going to die because they are told that they have cancer and the hospital cannot do much to them. And we tell to the patients ‘no one knows when one will die. You are given six months from the hospital but in reality we don’t even know whether we would wake up tomorrow morning after we go to bed tonight’. And we say ‘six months is quite long. So you should not give up. If you lose hope then the disease will harm you more’. That’s how we continue to give them hope that if one is able to purify ones past karma those 101 karmic diseases shall be cured. And so far there were not many challenges but one challenge is as my colleague mentioned about patients not knowing their disease and the prognosis.

Drungtsho 4:

Till now from my experience of seeing patients, this is my fifth year that I have been seeing patients, and I did come across cancer patients. They were mostly cervical cancer and breast cancer. By the time they come here they would have finished all the chemo there in the hospital and if it is breast cancer they would have had the surgeries. They (cancer patients) do not come here for a particular problem of cancer but mostly for so many other pains. And most patient do say that they have cancer and that they are on treatment for cancer from the hospital. Some would be still continuing the treatment. Here they will come for other pains like backache. So when we know that they are still on cancer treatment we do not give medicines from here. Because the cancer medicines are strong and so we do not mix our medicines with it. But for those who are not currently on medicines and there are some who will not be on medications and of course there are some who are continuously on medications. So for those who are not currently on medications we assess and although we do not have medicines to cure cancer but we give medicines to relieve pain. But we do give counselling and advice because most of them will be depressed because they have cancer. They will have lots of stories about their illness journey, what all they have resorted to and say that they have finally come to Traditional Medicine and we also counsel them accordingly just as my colleague said we encourage them, give them hope and tell them to keep their minds happy. Because everything is made by the mind and if our mind is happy the disease will also be lessened. So the mind has to be taken care. Even if they come to us saying they have cancer we say that cancer will not kill them immediately and we advise them accordingly.

Drungtsho 3:

So most of the things are already told by my two colleagues. But if I share one of my experiences, there was an old lady who had both her kidneys failed and she was no more on dialysis. She told that there is nothing to be done any more. When she was brought to me she was stable but her daughter was depressed because her mother was going to die. The hospital had told her that there is no treatment for her mother. And she brought her here. She was so affected that she lost her bag that day, she didn’t remember where she kept it and she was struggling with it. And her last hope was the Traditional Medicine and was telling me ‘if Drungtsho could give some medicines to my mom’. So when I did the assessment I was told that the hospital was clear that she was not going to survive but she was still called for review every month for ultrasound and also to see some kind of level in the blood report whether it has reduced or not. If that level reduced then she may live for some more but if it continues to go high then she will not survive. So they (patient and family) wanted us to help them by any means. But then when the hospital could not treat the disease with so many strong medications that they have we have lesser chance to cure and so we cannot assure them that we will cure them, you know. Nevertheless, we can counsel the patient and the family, when I say counsel I mean talking to the patient, the way we talk because I have experienced myself in the hospital when I go there sometimes with my patients I really feel that compared to the allopathy it is much better here in the Traditional Medicine. (*She laughs and continues*…) not because I work here and I say that it is better but I am sure patients must have noticed as well that compared to the doctors there (in the allopathic hospitals) we *Drungtshos* (Traditional Physicians) here are much more approachable in many ways like the way we talk, the way we care. Even when we go there as patients the doctors there are scary to even go nearer, you know. So for this lady she was given very short survival time from the hospital but as I sat in the OPD and followed on her for two years she continuously took our treatment and she was stable for two years and when she went for the follow up each month that level in her blood was decreasing (which was good).

**Probably that was creatinine level**

Drungtsho 3:

May be, and it continued to decrease.

**And she was not on dialysis, you said, right?**

Drungtsho 3:

She had dialysis in the past but it was stopped. And then I started working in the in-patient and I lost track of this patient. So even if patients are given short duration to survive, some, may be, depending on their past karma, if the treatment there (allopathic hospitals) doesn’t work it does work here (traditional hospital) sometimes (a good option for patients and families). And they will have so much of hope, with that last hope they come to us and I do feel that we are often able to help them. That is my personal experience because I have seen this patient myself (smiles).

**But she was doing well for two years, right?**

Drungtsho 3:

Oh yes and she was doing well for two years.

**So there is in-patient ward here as well? I didn’t know about it.**

Drungtsho 3:

Madam, you remember we developed the nursing curriculum in your faculty?

**Yes**

Drungtsho 3:

After that we completed the three months nursing course there in your faculty and following that we started keeping patients in the ward here.

**Do you also keep patients with advanced illness as well?**

Drungtsho 3:

We don’t really keep patients with advanced illness.

**So you treat them only on OPD basis?**

Drungtsho 3:

Yes we do admit them but not for acute care, you know.

**How about your experience (Drungtsho 2)?**

Drungtsho 2:

My experience is that patients come to us only when they have no other options left. I tell this to my patients sometimes. Because the patients would have lost hope on the allopathic side but they still have hope to live and when their hope and expectation for the treatment is no more only then they will come to Traditional Medicine. When that happens the patient is already diagnosed, you know, treatment like chemotherapy and all are completed but still we do not tell them that we cannot do anything for the disease. We do give them ‘false’ hope that they will be alright. But we indirectly, like my senior colleague mentioned, explain about the different types of diseases and we deal with patients from a Buddhist perspective. Like there were cases in the past where a patient with terminal illness received blessings from *Rimpoche* (an honoured Buddhist Master) and the *Rimpoche* had advised the patient to do circumambulation of Thimphu *Chorten* (The memorial Stupa in Thimphu) for hundred thousand times. When the patient made ninety thousand circumambulations the cancer was nowhere. So there are stories like this we can base on. At the moment I have one patient with cancer of rectum. And she has lost all her hopes. She was asked to undergo surgery but she didn’t want to do that so in such situation it is not just her but the whole family is traumatised, you know. So the challenge is not just to her but for the whole family. And each time they (patients and families) come to us we can make out from their face that they have so much of expectation from the way they look at us, the way they talk to us. And we are not professionally sound to give palliative care, you know. So what we are doing now is the whatever little we know we just use that experience. And sometimes I reflect about it in the evening/night and I feel so hopeless and helpless because we just witness the death of the patient in front of us. That is one of the challenges right now. (Limited knowledge on PC)

Then the other thing is if I talk about future scope, we have started a collaboration where every month the doctors and *Drungtshos* make clinical case presentations, exchange knowledge where we supplement and that is one platform that is created. So here after I think when it comes to palliative care also, right now of course the model is not developed but when you develop the model if you could gather knowledge and opinions from different stakeholders and come up with a very different (practical) modality I feel that will really help. Then the other thing is till now, this is my fourth year in the service, I have not really been able to handle terminal cases. Not many have come to me but even those few who came, when I was attached with the hospital, I would directly refer them to the allopathy doctors because I thought they need immediate interventions and those days there were challenges right from having certain equipment in place. But here in the Traditional Hospital we do give symptomatic treatment and some do respond to the symptomatic treatment. And one thing is that *tshosak drip jang* (accumulation of merits) and practicing those core Buddhist values is what we emphasize to patients.

**How do we directly translate *tshosak drip jang* into English? Is it accumulating merit?**

Drungtsho 1:

Accumulating merit and purifying negative *Karma* (Past negative actions)

Drungtsho 3:

It is basically accumulating merit and purifying bad karma.

Drungtsho 1:

It is to cleanse (one's past negative karma)

**So it is that mainly accumulating merit through positive actions?**

Drungtsho 1:

Yes, that’s right.

Drungtsho 4:

So along with such kind of guidance and advice and whatever possible traditional medical interventions are possible we continue to do it. Till now that’s all we are able to do. And more than that we are not competent.

**As per my understanding, and as mentioned by *Drungtsho* 2 earlier, usually patients come to *Drungtshos* only when there is nothing possible from the allopathic side, as an alternate option.**

Drungtsho 4:

It is not always like that but there are few cases

Drungtsho 3:

I also feel it is few cases who comes when there are no options available.

**Do patients come at an initial stage of the illness? When it is not even known that they have cancer or they are just diagnosed? And if so, is there a system where you refer the patient to the allopathic doctor? How does that work?**

Drungtsho 4:

Since very recently after the collaboration with the modern medicine is started we have developed some protocols. So this collaboration has created a forum and we have developed certain forms, referral forms are developed, and so if the patient is in need of immediate investigations or further care beyond our capacity then we directly refer them to the allopathic doctor. We call the doctor to inform about the referral and the doctor does all the necessary things. And in the same way we are practicing otherwise.

**So you get referrals from the doctors as well?**

Drungtsho 4:

Yes, yes.

**Oh, that’s very encouraging.**

Drungtsho 1:

Can I add something?

**Oh yes, yes, please**

Drungtsho 1:

So when it comes to referral it is how my colleague has just explained. But for those patients who comes to us at an initial stage when it is not known that there is cancer, for example, a case of a lump which doesn’t give any pain but is increasing in size comes to us we do not start any therapy here and we refer them directly to the allopathic doctors.

**Like a breast lump?**

Drungtsho 1:

Yes, and for such lump if we give needles or do any interventions then there are chances that it can spread. So if we doubt then we do not initiate any therapy but if it is mild case then the external therapy doesn’t really harm but an advanced interventions like inserting needles and all can be risky. So today the referral system has improved. In the past we used to lose track of the patients, we of course used to send patients asking them to go to the hospital but we wouldn’t really follow up but now after having started the collaboration the system is really good.

**That’s so good. I am glad the collaboration has finally happened.**

**Now my next question is what are some of the needs of these patients cancer or any other advanced illness, where there is not much of cure prospect? Like some might have pain. And besides the physical pain what other needs do you see in these patients?**

Drungtsho 1:

So with patients that I encountered I think I could not really understand what their psychological and other needs were but in my opinion, when the patients are with a terminal illness I feel they need emotional support and for some they do have that support from their family, right? But for those who do not have support from the family I really feel it is high time for Bhutan to start palliative care. As the country develops diseases like cancers are increasing and most of the people do not have time to care for their ailing parents because they are all in the jobs and they have no time for their parents. And the parents expects the children to be with them and talk to them but when there is no time for all those the disease also becomes worse and even if they were to survive for five to six years they would die within three to four years.

**So you mean psychological needs are significant?**

Drungtsho 1:

Yes, psychological issues can deteriorate the patient’s condition. So if palliative care is started it will have a care provider and from my opinion it should also have some spiritual components like access to Buddhist teachings, some mindfulness practices. If the patients are very critical who cannot sit up that’s a different issue but even for them they can be provided teachings that are pre-recorded which they can listen lying down. So I feel all these support should be provided. And when palliative care centre is initiated if it can be collaborated with traditional medicine we have many pain relieving therapies here you know. That’s purely my opinion. And till now I don’t really know other needs in such patients. But I feel all these will help such patients

**What are some of the pain relieving therapies that are available here?**

Drungtsho 1:

Like I said earlier, for example if it is whole body pain which is sometimes difficult to assess. So we categorise the patients’ symptoms. Whatever may be the diagnosis made in the allopathic hospital we assess the patient and establish our own diagnosis here to assign a therapy. So accordingly we have separate therapies for heat and cold. So if the patient has so much of internal heat where the fire element is significant then we cannot give heat therapies such as steam bath like herbal steam bath because it is like adding fuel to the fire and it will get worse. So for such patients we do dry cupping therapy, massage and we also have a therapy where we can reduce the heat if the heat is excessive, like water therapy. Then if the patient’s cold element is very high we can add little bit of heat. So when the internal humour becomes balanced definitely their pain will go away and they will feel relaxed. So we have all these therapies. For heat therapy we have steam bath, then we have gold needle therapy if the pain is localised. So that is needle therapy.

**So is acupuncture same as gold needle therapy?**

Drungtsho 1:

It is different. We also have acupuncture which can be used as pain killer. So that way I feel we have lots of opportunities for palliative care.

**That’s great. I can see so much of potential in the traditional medicine. Umm…I just wanted to ask do you all know what palliative care exactly is.**

*All four shakes head indicating that they do not know what it exactly is*

**So palliative care is an approach of care to improve the quality of life of patients diagnosed with a a life- limiting illness. The focus of palliative care is both patients and families because it is not only the patient who suffers but the family equally suffers as well. The whole family is disturbed, right? And the main objective of palliative care is to give symptomatic treatment including physical symptom management for pain, constipation and headache not just that, because when the patient has a terminal illness they go through what is called ‘total pain’. And total pain includes physical pain, psychological pain, emotional pain and spiritual pain and the pain of the family members as well. So because there is so many different types of pain when diagnosed with an advanced illness it is termed as total pain. Palliative care can be started at the time of diagnosis because at the time of diagnosis, like for example, a patient is waiting for her biopsy report and finally she gets the report and she is told that she has breast cancer. It is not only the physical pain or discomfort that the patient goes through but then that mental, and psychological and that emotional and spiritual distress and everything you know. And she needsto go through surgery, the chemotherapy and all you know. So palliative care can be provided along with other treatment like surgery, chemotherapy and so on. Then the quality of life becomes much better. Studies have found that those patients who had palliative care from the time of diagnosis along with curative treatment are found to live longer with better quality of life compared to those who did not have palliative care.**

**So now that you have understood what palliative care exactly is and with our earlier discussion, do you think that PC training can be helpful for you?**

Drungtsho 4:

When it comes to the training, actually we did learn about cancer in our *bso-wa-rig-pa* (Knowledge of Healing, pronounced as so wa rigpa)) curriculum. According to the *bso-wa-rig-pa* cancer is to do with the changes within the body. And now more and more cancer patients are coming to see us. So I think if we are included in the short term trainings on palliative care we will be able to render better service. I think we will be more comfortable and confident to help patients. Because at the moment most of the time we are helpless. Even when we have patients in front of us we do not know how to communicate well and later in the evening at home we recollect about the helplessness and that way patient will just deteriorate. Instead if there is a training for us at any case, by any means, we will be able to help the patient better. So some kind of PC trainings would definitely be beneficial I feel.

Drungtsho 3:

As we discuss about the need for palliative care training and the need for the ‘total pain’ we do have a separate chapter in our curriculum about how to deal with patients, how to care for them and I feel that is related to palliative care too although that chapter do not exclusively cover about total pain. In total pain if we take an example of a patient with an advanced illness his/her family is largely affected. So we did not learn about taking care of the family. So as my colleague mentioned if we receive some kind of training where we are updated on taking care of the total pain and how to relieve such pain through group discussions and experience sharing I think there will be lots of benefits.

**Palliative care is a multidisciplinary approach and the team usually involves a doctor, a nurse, a psychologist you know, then a spiritual person, as per the religious background of the patient and the family, and also the physiotherapist, pharmacist. So all these different disciplines are involved in the team. And in our context here in Bhutan, definitely *Drungtshos* can play a big role. I am understanding that you not only have the competence for physical therapies but you all also have so much competence for the spiritual wellbeing of the patients and families. And so all these different disciplines coming together, having a common training would definitely be beneficial.**

Drungtsho 2:

The other thing is that there is nothing that is not included in our *bSo-wa-rig-pa*. It actually covers all these aspects in general but today what science has done is that it has developed specific modalities in patient care and so if we are included in the palliative care team then we can contribute our *bSo-wa-rig-pa* knowledge into the scientific context then there can be an opportunity for a new essence, a new knowledge or a new value. The other thing is, *bSo-wa-rig-pa* is more related to the Buddhist essence whereby the care will not only be based on the scientific aspect but also from a Buddhist aspect which would be more beneficial. And since majority in Bhutan are Buddhists I feel it is really compatible if we can develop a modality.

**As you mentioned earlier that some 104 diseases are due to the *Karmic* causes which are as a result of one’s *karmic* actions. How easy or challenging it is to explain this to your patients? And how do they respond?**

Drungtsho 1:

When it comes to this, since I have not come across more than two to three patients with advanced illness and they were all Bhutanese Buddhists so when we advise them to practice Buddha Dharma and when we tell them that Buddha Dharma is not just for the benefit of the present moment but will also benefit at the time of dying it is not very challenging. And I feel this will not be challenging even in future for us in Bhutan because as Buddhists we believe in cause and effect you know. We believe that even if it is not going to benefit us now it will benefit in our next life. Like it is said that even if the knowledge we gain in this life does not benefit in this life it will benefit us in our next life. So likewise most of us are aware that the Buddha Dharma that we practice now can benefit us in our next life as well and so it will not be so challenging at all. And your idea of developing palliative care in a Bhutanese context is very good. Because if we take our context elsewhere then it will be challenging. It will not work because of the variation in the belief system. Although I have come across only few patients so far and there were no challenges, I really feel that even if you include it in the model it will not be a challenge. And that’s my opinion.

**Drungtsho mentioned a very important point here that if palliative care is developed according to our context then there will not be any problem. That’s the main objective of this research project. You know palliative care exists in many parts of the world like in Australia, UK, the US, in Kerala in India. It is all very good for their own context like their socioeconomic status, their culture. The WHO emphasizes that palliative care has to be according to the cultural needs of each country. Otherwise I could have just borrowed Australia’s model of palliative care or even we could bring in India’s concept. But then as Drungtsho mentioned, we cannot borrow somebody’s palliative care model. We have to have our own context based model.**

**Now, my next question is, although majority of the Bhutanese are Buddhist but then, as a care provider, we also have to remember that a small proportion of our population also follow other religion like Hinduism and we do have Christians as well. How is your approach with patients of other faiths? I think my assumption was that only Buddhist patients come here but this afternoon I also saw an Indian patient in front of Drungtsho’s chamber and I became curious. So how is it?**

Drungtsho 1:

Actually, in the past, Traditional Hospital was mostly opted by the elderly Buddhist Bhutanese but these days, although we continue to see more of Bhutanese patients, but we also increasingly see tourists who are interested in Traditional Medicine. And likewise the Indian labourers, and Nepali community (Bhutanese in the southern Bhutan are of Nepalese origin) also comes. However, since we do not assess what religion they belong to we don’t really know their religious background. We don’t ask that. So far we treated them with the focus on the disease. But if we are to provide palliative care we then we will have to ask their religious background. And even then, when providing palliative care, we can ask them to invoke their own faith based idol and help them I feel. As a care provider it is not right to ask everyone to practice Buddhist values if they are non-Buddhist and just because they are in Bhutan. Since they will have their respective idol to worship we can ask them to have faith and pray accordingly. In *bSo-wa-rig-pa* there is something called *milam gi takpa* (signs from the dreams) which is known as *Jigtey*.

So when you see certain dreams it actually indicates that the person may die soon. What is advised is to accumulate merit by doing positive actions. For example, if a person recites *Mani* mantra (Mantra of *Avolokiteswara* – Buddha of compassion) hundred times every day then he/she is advised to recite thousand times every day. So likewise be it Christian or Hindu, they will have their own idol to worship every day and if they can double or triple that effort of devotion in their own ways of practice and prayers I am sure it will have benefit. Of course we don’t directly focus on the religion when we treat patients. And before I forget, we mentioned about *tsho sak dribjang* (accumulating merit through positive actions) earlier and for that I think it is not only the Traditional Medicine’s perspectives but would also be good to involve the Central Monastic Body (CMB) and the *Lams* and *Khenpos* (Buddhist Masters and Buddhist scholars). For us we have learned that 101 diseases are due to our past *karma* and so we got to counsel the patients accordingly. I feel it is very important to involve the CMD to really help with the practices on how to purify the *Karma*.

**The main reason why I have asked you about the religion is because till now we do not have such multi-disciplinary team to care for patients, right? In the conventional medicine, religion hardly comes into the picture of patient care and so patients, irrespective of any religion, goes to the doctor and there is hardly any conversation about the religious background there, right? And in Bhutan we are predominantly Buddhists and often everyone takes that for granted.**

**However, in palliative care we also have to be open to any patient with any religious background. If a Muslim patient happens to be in our country, say for example, a Muslim Indian labourer who is ill with an advanced illness and chooses to die in Bhutan, then we need to modify our approach of care for him, you know what I mean? He is dying and we have to respect his beliefs, not necessarily that we have to start preaching or learning about Muslim culture but then we can modify our approach of care to facilitate and fulfil his and the family’s wishes you know.**

Drungtsho 2:

If there are differences related to the religion, I feel, there may or not be challenges in palliative care. However, I feel we can contextualise it into, what to say, values, right? Like we can provide care based on human values. Values can apply to anyone. So we can approach not from a religious point of view but as values. Whatever we do values are important and when we think about it I feel there won’t be much problem there.

Drungtsho 1:

So here if I can go a bit in depth, actually, *bSo-wa-rig-pa* is said to belong to no one. In the times of Buddha there were Christians who came to listen to His teachings and many others with different faiths. There were humans, angels, deities as well. Today although Tibetans claim that Traditional Medicine originated from Tibet and we Bhutanese claim it is our traditional medicine (*laughs*) where we try to kind of own it but *bSo-wa-rig-pa* actually belongs to none and so it is for everyone.

**That's very interesting. Thank you Drungtsho.**

Drungtsho 1:

Yes, because as I said during the times of Buddha, there were Christians following Him. So *bSo-wa-rig-pa* actually should not pose any discrimination related to religious background. However, that is my personal understanding. So once we start the palliative care service/centre now if the patient and family really wants to do elaborative rituals in the centre then there could be issues. Otherwise asking the patient to invoke one’s own deity/idol and to provide treatment should not cause any issues. Like I was in India in the past and you must be aware of the Lotus Temple (in Delhi). So this Lotus Temple is empty inside you know. There is no statue of any Gods or Idols or Buddha. So anyone can go there, remove the footwear, get inside and invoke one’s own deity or idol. There is nothing there. So we may develop palliative care approach in that way. Or we may have a separate halls for meditation, for example. So at the time of implementing it may be necessary to involve the relevant stakeholders, discuss with them and I don’t see any issues there. But when it comes to *bSo-wa-rig-pa* there is no discrimination at all. Everyone is same.

**Thank you Drungtsho. I think I was really ignorant about it. With my limited knowledge on Traditional medicine I was always thinking that it is more related to Buddhism. I now understand that it is so open to everyone and that is even more encouraging for palliative care.**

Drungtsho 1:

Lord Buddha has said, although it was in Sanskrit, that through His innate blessing he could help everyone to relate to their own idol and likewise when we hear the teachings today in our language it is actually the same. It is to meet our individual needs.

**I see. Thank you Drungtsho for this wisdom.**

Participant 2:

One thing is in Bhutan *bSo-wa-rig-pa* is known as *Nangpai men*, and *nangpa* is often misunderstood as Buddhism but actually it is not like that. *bSo-wa-rig-pa* is the art of healing or the science of healing when we directly translate it. So when we say art of healing there is no religion. But in our context it is because we are Buddhist and when we know that it is taught by Buddha we are trying to contextualise and we say that it is our Buddhist medicine. Although Buddhist values are inculcated there but if we are to provide palliative care we can always segregate the Buddhist values and relate it to the science of healing. So in that way challenges can be minimised.

**Thank you very much for helping me understand this better. It has been a big education for me.**

**So to summarise what we have discussed so far, we have started with what motivated you all to participate in this discussion, and then your experiences in managing patients with advanced illnesses and the challenges, right? Then we started discussing about what is palliative care and we also tried to clarify what palliative care exactly is, right? And we started to discuss on people’s faiths and people’s culture and people’s religion which was very educative to me at least. Do you have anything else to add on what we have discussed so far?**

Drungtsho 1:

I don’t think there is anything more to.

**Now that we are all clear that palliative care is required, right? So the next point of discussion is, if we are to start palliative care, how do you feel about the human resource and the infrastructure? Do you feel that we would first need more people and then start? Or can we start with the existing staff as the future graduates come in as usual?**

Drungtsho 1:

Aah… if we are to start as a centre (separate PC centre) it is a different issue, right? Because then we will have to focus on the infrastructure for the patients to be kept, the care provider. So there should be a separate set up. But to initiate (PC) within the health system, so long we get the essential trainings, I feel we can give palliative care within the existing infrastructure and the staff. Even with manpower there is nothing very different that is required I think. Because it is mainly to do with the mental, emotional and psychological issues and the symptomatic treatment. And we have mindfulness therapy here in traditional medicine too. We have started it with other healing therapies. And all these can be provided according to the need of the individual patient. I don’t have to say about the allopathic medicine but when it comes to Traditional Medicine I feel we can start it (PC) but before that we would of course need a kind of training on how, when and whom to provide palliative care. How to do the recording, supervision and monitoring and if we are trained on these things I think we can manage with the existing manpower and the infrastructure. I don’t see anything extra and we had been doing it in some ways I think but what we lacked is those aspects like the reporting and all those

**Thank you Drungtsho. Training is definitely important and necessary.**

**Now the other thing is, pain like cancer pain is almost always moderate to severe at an advanced stage and in the allopathic side it is usually managed by the opioids like morphine. If patients come to you in such a severe pain do you have medicines to manage such severe pain here in the traditional medicine?**

Drungtsho 1:

I think we do not have such strong pain killers here. And if I add something here. Actually there was a research that was conducted and I am not very sure who the samples were. A group of Tibetan doctors have researched about what we call *Rinchen ribu,* the precious pills. And they have found that this *rinchen ribu* has cured hepatitis B, then having treated breast cancer but then I don’t know how they have done the bias assessment. So there are reports about all these. If we think about including these drugs we can do a research here in the traditional medicine and implement it accordingly that would be good I think.

Now when it comes to pain killer drugs for severe pain we don’t have it at the moment. All we have is acupuncture for immediate pain relief and nothing more. I think our therapies here will not really be of benefit in case of severe pain, right? (*asks the fellow participants*) I think we can manage till the patient has moderate pain but if it is a pain to be managed by opioids then it will be a challenge for us. However, if allopathic and traditional medicine comes together, like when the two hands comes together there is sound produced, I can see some fruitful results. Pain can be managed from the allopathic side and we can support through other supplementary therapies. It will be really very good.

**That would be excellent. As you said pain can be adequately managed from the allopathic hospital and if you can provide other healing therapies along with the emotional and spiritual support, right? What a beautiful team work that would be.**

Drungtsho 1:

Yes. That will really work I am sure. But if we are to manage here all by ourselves then I think it will be a challenge for us.

**Any other opinion besides what we just discussed?**

**(*Everyone shakes head to indicate there is no other opinion*).**

**Now we will discuss about creating awareness on palliative care. How do you see about educating and creating awareness to the general public? How should we start? When to do it? How should we go about?**

Drungtsho 2:

Here I think first we, health workers, need to be well equipped and prepared. So like if our preparation is likely to be over in a month’s time then if we start with creating awareness. That would be better because by the time the message reaches across in a month’s time patients might start coming forward, that’s what I feel. So first start with preparing the health workers and when there is about a month left to complete with the health workers then start with the general public. If we start advocating immediately and if the health care providers are not ready then it will not be good. It is not a one or two individuals’ task right? You need to involve stakeholders, requires certain resources. So we need to be ready first.

**Any other different views?**

Drungtsho 1

I think, first train the health workers.

**Thank you. I think that’s a great idea.**

**Do you have anything else to discuss besides what we have done so far?**

*Everyone shakes their heads*…

**Do you have any specific advice or suggestion or a comment for the project?**

Drungtsho 3:

Let me say one thing. It is very good that madam is doing this research to start palliative care. I really feel that if there is palliative care it will really help patients to be happy even at the end of their life, their remaining days can be satisfying, and if they have peaceful deaths it is such a merit for us, right? So I think we became very interested in your project madam and we are interested to participate in it.

**Thank you very much.**

Drungtsho 4:

What I personally feel is firstly we, the palliative care professionals, need to develop on our attitude because the patient is already in such a stage where there is nothing possible for him/her and if the doctor is rude then we will be in fact letting the patient down rather than helping him/her. So from my opinion it is very important to develop our communication skills, the way we talk to the patients and families, to deal with them. I think we need to really prioritise on these things. No matter how good one’s mind is, sometimes people do say ‘oh this nurse’s way of talking is like that but her mind is very good’ but nobody can see the mind, right? First thing is the way you present physically and that is what seen by others and how much ever good the mind is it is all destroyed by the way you talk or present yourself to others, you know what I mean? So the first thing that I feel very important is to develop on how to communicate with the patients and families. And madam has already started this research and I do hope and wish that all goes well as aspired and I have my best wishes for you (*smiles*).

**I totally agree. Thank you very much.**

Drungtsho 2:

I don’t really know whether it is a comment or a statement but I have got two things to say. One thing is that I can see something emotional within you. As a researcher you have really felt that pain and that is very good. So please do not leave it through half way. Keep going. If there is any necessary help that we are capable of helping we are definitely ready to lend our helping hand. I would encourage you to keep pushing on what you are doing. Second is from the time you start or as you start training people the model should inculcate the ethics and values so that care givers have a different set of values and ethics in them. It is not that we do not have ethics at the moment. Modern medicine has lot of ethics to study about, in traditional medicine we have a module on ethics but still we fail because I think it was just done as a part of the course. I think it never touched the heart of the caregivers. So therefore, develop a model that can really touch the core of the heart which can be started small because I understood that palliative care is not just the physical treatment but so much emotional and psychological support. If the model can help the person to transform within I can see that there will really be a unique charm in your model. That’s what I wanted to say.

**Thank you very much. I will note this down.**

**Thank you very much everyone. I am so happy and I am so touched that palliative care is taken very positively and I look forward to working together, seeking your assistance. As Drungtsho 2 said ‘if help is required’ and this, your participation, is a big help for me. Your information and your time is invaluable. Thank you very much la.**

Drungtsho 1:

We have our full support la

**Thank you so much.**

*Strength for Bhutan: having both allopathic and traditional medicine co existing under the ministry of health*
