# Supplementary material for: Healthcare professionals’ views on how palliative care should be delivered in Bhutan: A qualitative study
Source: PLOS Glob Public Health. 2022 Dec 12;2(12):e0000775. doi: 10.1371/journal.pgph.0000775 (PMC10021767; doi:10.1371/journal.pgph.0000775)
Supplement: S7 Data — (DOCX) [file pgph.0000775.s008.docx]

**FGD with HCPs ERRH, Mongar on 18.5.2019**

| Participant 1 | Anaesthesiologist |
| --- | --- |
| Participant 2 | General surgeon |
| Participant 3 | Gynaecologist |
| Participant 4 | Drungtsho |
| Participant 5 | Pharmacist |
| Participant 6 | Nurse in-charge Dialysis Unit |
| Participant 7 | Nurse in-charge Medical Ward |

**I would like to thank each one of you for your time to participate in this discussion. Can we start discussing on what motivated you to participate in focus group?**

Anaesthesiologist:

Firstly, because as an anaesthesiologist, I am involved in treating pain, be it cancer pain or neuropathic or any acute surgical pain. So may be because it is my field and that’s how I am involved in palliation. Secondly, I think we have seen many cases with advanced cancer pain, and I think also because as a Buddhist you know. So may be because of these two factors I feel that palliative care is very important and I want to be involved in palliative care. So that is how I am motivated in attending this discussion.

**Thank you very much Sir**

General surgeon:

Thank you. This is my 16th year as a civil servant and working as a medical professional. Now over these 16 years and even before also our life expectancies are increasing, we saw in the past and we will see in the future many cases, especially cancer cases where the incidence is increasing very much. So in that way so many people, so many patients will require palliative care at one point of time. I think it is not too late that our ministry looks into it and congratulations to you for taking up this very important topic. So that is the only single factor which motivated me to take part in your research project because we need this palliative care and end of life care so much in times to come. Thank you

**Thank you very much sir.**

Gynaecologist:

If you now look at Bhutan’s health care system palliative care is actually not known. There are I think few nurses trained in palliative care but after they came back I think they started doing the same. So no one is actually trying to, you know, construct or built this type of services for the people actually. So it is not available at present and what I hope from your study is that it will have some great impact on the health system. May be this will have impact on the ministry and then my focus I think is actually palliative care is one of the health care means, you know, in the health care system. So that’s why as a clinician and care giver I thought I should also take part in this study.

**Thank you Sir**

*Drungtsho*:

I am a traditional physician. So when I go back to my past experiences and when I think about the care that we have to give to the patients, *bSo wa Rigpa* (term used for traditional medicine, pronounced as sowa rigpa) is also a holistic care and palliative care is one of the holistic care for the patients. So that’s why having dealt with patients for so long I think palliative care is very important because it is already included in traditional medicine. However, we have not come up with much of development you know. So this has motivated me to participate in your project. Thank you

**Thank you Drungtsho**

Pharmacist:

Good afternoon. As a pharmacist I felt that my responsibility is ensuring the availability of drugs required for palliative care and indirectly I feel I am involved in palliative care activities. So that’s why this is one of my motivation to I mean ensure the availability of medicines and all. That’s why I am here to participate.

**Thank you madam.**

Nurse Incharge, Dialysis Unit:

Good afternoon. I am from Dialysis and we have 27 patients undergoing dialysis at the moment. This service is started from 2010 in this hospital and as far as I know only ten patients went for kidney transplant but out of ten only five got the transplant. And most of these patients who are on maintenance dialysis are staying in the hospital guest house. I think these people often feel that it is their end and not much can be done here after. And we also cannot do much because we have not got such facility at the moment. What I feel is may be if you could come up with this palliative care this might help these patients. These patients (patients on dialysis) are often found to be moody, sad and that we (nurses) have to often accept when they throw tantrums as this is the reality of their life. Nothing much can be done. Most of them are not able to avail the transplant and I think this is one of the reasons why they throw tantrums, pour their anger on the staff. Some of them are even throw away their (dialysis) appointment books. So I think through this palliative care may be there will be some improvement, at least in the outcome of their mood and that’s why I came to participate.

**That was interesting. Thank you**

Nurse Incharge, Medical Ward:

Good afternoon. I work in the medical ward. I have come across many patients whose disease condition cannot be cured and so live with the disease till they die. Patients are admitted for palliative care where we (health care professionals) are limited to only pain management and few other comfort measures. So with your new concept of palliative care I think I am going to learn more if it gets through and if you come up with this new program in your faculty (Faculty of Nursing and Public Health). I think we should start palliative care in every region focussing in the central, east and south. There are so many people who cannot go to the higher centres for such care.

**True. Thank you so much.**

**Now, the next point of discussion is, if you can share your experiences in taking care of patients with terminal illness like advanced cancer or any other non-communicable diseases which does not actually have the prospect of cure. How has it been so far?**

General surgeon:

I have come across quite a number of cases that were initially referred to India. Most of the cancers that are diagnosed in our context are always advanced at the point of initial diagnosis. So most of our patients, especially cancer patients, are sent back for palliative care. So in that way I have quite a number of experiences of seeing patients with advance cancer but, to be frank, with very limited knowledge apart from managing pain which also is very difficult. So more or less the palliative care that our patients are getting, I personally feel, are similar to the care given to other patients, conventional patients. In palliative care, I mean there is medically no hope for that patient, that he/she is not going to come back but the other aspects, the social aspects, the spiritual aspects, then how do we prepare him/her to accept the definite which is death is a challenge. And unless we have a good team starting from physician to nurses to spiritual heads to community, families, relatives. Even if we have the best technical people and medical people because personally I feel the medical people may not be able to do much for such kind of cases. So my experience is that we have come across so many cases but the service or whatever the patient requires or deserves is absolutely not enough.

Gynaecologist:

Palliative care as such actually from the time I graduated from India I would like to tell that the first time I experienced how a patient dies. It was I think a chronic obstructive lung disease patient who was gasping on the bed you know. So I was trying to see how patient dies, never experienced before. That was during my internship. So I was seeing he was gasping actually and I told my professor and Sir said ‘don’t touch’ *laughs*..The professor told me don’t touch. And in a while he (patient) actually expired. So then I carried the same experience and when I was in Yebilaptsha (*a district hospital in central Bhutan*) a Ca cervix patient was left in the hospital to die.

The family members wanted the patient to die in the hospital and did not want to take her home. Actually they must have wanted palliative care to be provided in the hospital but since we, doctors and nurses were not educated enough to give palliative care you know. So then the patient died and I also applied the same logic and the same technique as told by my professor (*everyone laughs*…). And Bhutan is a Buddhist country and most of time it is thought that it is your *Karma* (the principle of cause and effect) and you blame the *Karma*. We never try to improve what is available and possible but we often blame other things. That’s what I feel but now as I experience more and more as a medical professional I think we definitely lack this palliative care services.

**Very interesting Sir. You remembered your professor and maybe the professor also learned that from his professor (*many laughs*…). When he said ‘don’t touch’ how did you feel as a novice doctor?**

Gynaecologist:

Actually later on I learnt why they were not allowed to touch patients because that is the time when patient is actually trying to gain higher knowledge or trying to go to heaven *laughs*…

Anaesthesiologist:

So it is social component here I think.

**Or maybe cultural and spiritual component.**

**How is it today when a patient with advanced cancer cervix dies in your ward?**

Gynaecologist:

Today we know that anyhow the patient has an advanced cancer and that they will die but before that we don’t try to do lot of things. Once I was in Tsimalakha (*another district hospital under Chukha District*) a breast cancer patient was kept there. I think breast cancer had involved her lungs and she was oxygen dependent. And actually I once had a quarrel with her (patient) also because you know she was consuming one cylinder of oxygen every day and we were running out of stock. Then I actually told her that she has to go to Thimphu (*Laughs*…) and patient felt very bad because she thought we were not trying to give her enough care and that because of one oxygen cylinder, you know, we were trying to get rid of her. She thought that we were making lot of mess you know. Then later on I thought bring more oxygen and give it to her. So these are some of the small things that really counts. We have to look at both the sides I think on what is possible and available and what is not there. But regarding not touching the patients at death I think it is good. We are learning what we are supposed to do and when someone is dying and what we are not supposed to do.

Nurse In-charge, Medical ward :

I want to share some of my challenges. Patients who needs palliative care should be kept in a separate room where they are seen by different group of staff so that there the concept of palliative care is not mixed up. If we keep the patients who need palliative care in the same ward with the other patients I think their care is compromised because we cannot provide them with whole hearted approach to that patient and their family. That’s why I think the patient and the family members are frustrated and the staff who are taking care of general patient and patients requiring palliative care are also frustrated.

Anaesthesiologist:

One aspect of palliation is in the ICU. A patient who is already brain dead and there is no hope of coming out. One issue that we face, I think even in JDWNRH, I don’t know how it is today but during our residency the problem was when to withdraw the ventilator. And when we talk of palliation it is not only the patient but also the people around the patient (family). That’s why the challenges we face is we do not feel confident to withdraw and stop the breathing. In fact scientifically we know that patient is brain dead. So those are the challenges. Even the patient party have the same feeling. They do not want to abruptly withdraw and we have to use all the facilities that impacts the cost. Sometime we have to keep going the ventilator not expecting the patient to come out you know. This is the challenge. May be when palliative care comes in this things will be thrashed out and reach to a conclusion where there will be some protocol and all you know.

**Very interesting. You are right and this issue is not only in our part of the world. I have read that withdrawing devices is one of the main challenges everywhere but with a strong palliative care team things will be much simpler because it is the mainly communication that is involved when a patient is on the ventilator and when it is understood that there is not much hope having the patient on ventilator. Communication is one very important aspect in palliative care.**

**So those were very interesting and important experiences. Now, with your experiences in having taken care of terminally ill patients, what are some of the most important needs among those patients and families?**

General surgeon:

The biggest needs to provide to those requiring palliative care is those basic care and thats a big challenge. This is what I found. Who is going to provide those basic needs? They (patients) are not able to pass urine, they are not able to pass stool, they are not able to eat well, they are not able to change their positions, and this is the biggest challenge. Now with your palliative care research and whatever the findings, whatever the outcomes from your research and in collaboration with the Ministry, I think the first thing we have to address is how we can take care of those basic things. At the moment our biggest challenge is this. Now in this forum I will have to say that people who deal with urine, blood, stool needs to be rewarded little bit more. Otherwise there is no one to do all these (*expresses this in louder voice*...) who is willing to work on all these wholeheartedly. So palliative care will not only be pain management. If you take care of these then your mind can justify that you have given the best palliative care.

Anaesthesiologist:

I think it is mainly I feel that from the anaesthesia point of view we have enough drugs for pain management for palliation. I think there are some interventional pain management which will need lot of specialist but we do have the basic medications and drugs for pain management. As said by sir (Participant 2) here we need a set of people who are dedicated and I think structurally we have. We just need people who really are there for palliation and who are trained and I think that would be the biggest step to be followed in starting palliative care.

Nurse Incharge, Dialysis Unit:

May be I will say from our dialysis point of view. Firstly they are always trying to throw their temper and their mood disorder.

**Can I interfere here? Why do you think they do that? Why do you think they are so angry at you or at the nurses?**

Nurse Incharge, Dialysis Unit:

Well, what they say is, especially those who are staying in the patient guest house, that they (patients on dialysis staying at the patient guesthouse) are frustrated because they cannot get transplant (kidney transplant) That is one reason.

**Is it because they don’t have donors in the family?**

Nurse Incharge, Dialysis Unit:

No one to donate. I think only in the beginning few of their relatives come to see them. At the moment we have an old couple who are both above 70 years and it seems that they were thrown out of the house. They were staying with their daughter for about eight years but now they don’t want to go back and they have been living in the hospital patient guest house for the last two years.

**Was it that the daughter threw them out because they were sick?**

Nurse Incharge, Dialysis Unit:

Yes. They said that the daughter’s family could not find time to drop them to hospital for dialysis and take care of food and other things for them. Here again, regarding food, all they (patients) want to have is those unhealthy foods like cakes and all such foods when they are on dialysis. They keep saying anyway they are going to die soon. And they also don’t want to control on diet. They only want to whatever they want and go on with dialysis since there is no hope for transplant.

Anaesthesiologist:

As an average, because you have 27 patients on dialysis, how many would you say belong to that category, who are really frustrated and who really don’t care for their health?

Nurse Incharge, Dialysis Unit:

Actually, they are the ones who are in the patient guest house and they are 17 of them. They (patients) do so many things like throw away the appointment books, show anger on us.

**Do they listen to what you…**

Nurse Incharge, Dialysis Unit:

*Even before the question is complete*…No, no they don’t want to listen. They don’t want to listen. They also want to have dialysis according to their convenience only and they also want to have food without control. They don’t want to listen. They even say that (*Translated from sharchop*) ‘the staff in the dialysis will say anything. We need to eat everything or else we will die’. That is what they say (*Others laugh*…). They only say they are going to enjoy (for the rest of their life).

**How do you feel brother, (Dialysis nurse) when they are so arrogant and ignorant about their diet and their own health? You are a very senior staff taking care of such patients for so long. How do you feel about them?**

Nurse Incharge, Dialysis Unit:
aah… Not much we can do (*Others laugh*…) At least we try to provide some food assistance through our Bhutan Kidney Foundation and some monetary help. We have found that even those well off people, having their sons in high position, do not want to stay together and they stay separately away from their kids.

Anaesthesiologist:

One thing what I feel is, as the time goes by even the circle of friends they (kidney patients) had once upon a time is withdrawing. How the natural thing goes on but what I feel is aah.. they are not engaged you know and they are not seriously ill and they can do something (rehabilitative needs/occupational support, like in Pallium India for spinal cord injured patients). So that way I feel that you need to have some activities where they are engaged, where they feel themselves important, because I think they are frustrated because they are left out and they feel they are not important. I think they feel like that. All their frustrations comes from there. Now they are 17 of them they can do a small activity, do something, you know, to be engaged and they will feel that they are productive and may be they can also make some money out of it. So I think we need to engage them.

Have you not got such plans brother (asks the dialysis nurse)? How many of them have certain skills like carpentry?

Nurse Incharge, Dialysis Unit:

Ya, actually, there are few who can do carpentry but many doesn’t have any skills or interest even. But some of the family members can weave and some of them have worked in the past in the construction sites but there are some who haven’t done anything.

Anaesthesiologist:

And those will be the ones who will start thinking about so many things.

Nurse Incharge, Dialysis Unit:

And they will not listen to whatever is said, regarding their diet, and even if there are some visitors they will not even be interested and they will not even take care of their surroundings.

**Traditional Medicine is a part of our health system, right? And our patients, especially when they do not have cure, they look for so many alternatives. Traditional Medicine is one alternative that most of us are aware of. Can we discuss about the role of Traditional Medicine in PC?**

Drungtsho:

From my point of view palliative care is very much necessary. Because in order to build and promote palliative care I think the collaboration between Traditional Medicine and allopathy medicine is a must. Because aah.. I don’t know whether it is there or not in science, aah.. because our Traditional Medicine text mentions that in total we have 404 diseases. Among them 101 diseases are not curable. So there are healing practices in the Buddhist philosophy like circumambulation of *Chortens* (monument) and like that. And also to promote palliative care from the traditional side we have also come up with this book which is about traditional meditation and exercise. So now we are working on it. So that is why I think if we collaborate we can promote palliative care.

**How about others?**

Anaesthesiologist:

I think Drungtsho is right. We have to collaborate and Traditional medicine should be integrated into PC.

**Now let‘s discuss about drugs for PC**

**Madam (pharmacist) can you please share your experience regarding availability and accessibility of morphine and other drugs?**

Pharmacist:

Aah… In the regional referral hospital we have almost all the drugs that is in the essential drug list but we do have problems. One thing is the timely supply which is an issue. Sometimes there is delay in getting the supplies from the central supply depot. That is one thing. Morphine is well utilised here in Mongar and at times we have to procure additional stock also. The other thing is patients who are referred outside the country comes back with a list of medicines and if some of the drugs are not available here we have to procure it through Form II which at times gets delayed. It is because patients will not inform us beforehand and they will come to pharmacy only when their stock gets exhausted and we, at times, are not able to issue immediately. May be they (patients) think that all drugs are available in all hospitals and that is one thing where supply gets delayed when we have to procure through Form II. (Issue in the system)

**Pain management drugs are available in the regional referral hospitals but not in the BHUs. The patient is there in the community but the drugs are not there in the BHUs. As a pharmacist, what do you say about this?**

Pharmacist:

Actually they apply Form III and take from our stock. It is available here and we can provide them unless they have asked from JDWNRH. Till now it has not been so difficult because we always had adequate stock but may be at times we are not able to and they will have to get from JDWNRH and there can be transportation problems.

**Sir what is your opinion as an anaesthesiologist?**

Anaesthesiologist:

Instead of Form III which requires definitely a process and transportation issue and we know our settlements are very scattered. So there is always interruptions in the continuation of the medications. Once you are Form III user, so it would be better if we extend the EDL (Essential Drug List) to the BHUs. Of course after ensuring especially cancer patients requiring opioids. Once they are on opioids for one month or two to three weeks they are actually very tolerant and they do not express so much of complications. If it is acute, for example, myself if I had not taken opioids and if I take 20, 30 morphine there is a risk of respiratory depression. Once chronic patient, having received for so long, it (complication) is very rare. So actually in those cases we can extend the daily supply as a routine supply at the BHU level. That way there won’t be so much interruption. (Need to train BHU staff on pain relief and PC)

General surgeon:

Now if we are thinking towards providing probably good palliative care or end-of-life care for the benefit of the patients, before we develop a team, now to begin with, educate the public that we have expanded services with dedicated and motivated people working in there. So his stay till he dies will be comfortable. So form a team, create awareness among the public, I do not know, I mean that, of course there requires to involve community, social workers, and spiritual people. So educate the public telling that we have all those facilities to keep such patients and that is one way.

And one thing I think you are all aware now that our economic development is having adverse effect on the social cohesiveness. We see many patients there sent to the hospital, may be they will be there for 3, 4, 5, 6 days after that they are left. We see lot of such patients. Recently I had one patient admitted there post I mean he was treated for Hansen’s. Ohhh….

Anaesthesiologist:

One with the bedsore?

General surgeon:

Ya…*went silent for a while*….so… it is challenging… this economic development really has adverse effects on our social harmony. The standard of living has not improved, cost of living has improved …*many laughs*…. and that is dragging people more towards making money and forgetting the…*laughs*….relationships, and other aspects, social issues. It is a big challenge when we look into all these aspects of palliative care. Best is …the answers are left to you (researcher)….*laughs*….as a researcher, because I mean for sure nobody has done a PhD thesis like this on such topic and nobody has better idea. You have better idea and after this four years of your study you can share some of your experiences and challenges over the period of time.

**May be as my study progress and the findings unfurl we might have more answers. Thank you sir.**

**Can we discuss about the needs of families?**

Anaesthesiologist:

In some families there may be one or two members and one of them has to come here and stay with the patient. That becomes really a distressful situation for the family because there is no one to replace and they need money. So their goal in such case is to take the patient home so that they can attend their routine works. And of course they want the patient to be comfortable at the same time. In that case pain management is very important. The family member already knows the prognosis and seeing their sick family member in lot of pain and becomes another distress for them. So after they are given the information that the patient’s illness is not curable and that the patient is given some time like three or six months survival chance they really want to take the patient home with adequate pain management to keep them comfortable. So there is a need to take PC as early as possible to the community.

Anaesthesiologist:

And one thing is madam, the chain of management so when you have one centre or one dedicated team in the hospital that hospital will manage at the higher level for certain period of time where the patient is prepared, the modality of pain management is prepared, the modality of surgical intervention is prepared and then they can then take the patient to the next lower level like the district and from the district slowly to the BHU and from there to their home. So this chain of management is important.

Gynaecologist:

Main thing is I think people who give care to such patient should have good attitude. Presently if you see in Bhutan although we say we are Buddhist but what we do is we are ritualistic Buddhists. Doing all these *rimdros* (rituals) and such rituals you feel that you are more Buddhist by doing that than actually helping somebody. Because chronic patients stay for long time with you and you get fed up at some point of time because you have your own agenda to do and lot of things. I think if people don’t deviate from what we actually preach and practice I think we can provide the same level of care and I think that should come from within your own mind not doing prostrations four five times every day. I feel that may not have any benefit but doing some good things I think that carries lot of weight. That will actually take you to heaven.

General surgeon:

Regarding the need of the families, now in our context the need of the family members of patients undergoing palliative care depends on the socio-economic background of that patient. If the patient is socially economically well off, they have certain knowledge, certain knowledge of palliative care then the demand will be more for instant pain relief, to provide all those things. But if you have patients’ relatives or families with low socio economic status, who do not have knowledge to provide them adequate information on palliative care it is a different story. So every patient who needs palliative care or end-of-life support should be provided with adequate information be it whatever the socio-economic background. So unless we provide those information I think they will not know and…*laughs*….they will always take back the patient. So I think that socio-economic demography should be well assessed. Actually the need for palliative care specialist is genuine, team requirement is genuine but till then we can start. We need not wait for that perfect team to come into picture but it is a challenge. But definitely the need of family, I have one patient here 22 year old boy diagnosed with small cell tumour, advanced, nothing can be done but for him there are enough relatives, quite well to do, quite frequently at night they are having problem with the nurses. The problem is because patient is demanding painkillers, off and on patient is demanding painkiller and I don’t know sometimes…*laughs*..staff are also getting irritated. So when I go in the morning I just say just don’t worry but give him painkiller. So there are so many such differences.

**I actually interviewed that patient and the family member yesterday and one of the biggest needs the family mentioned was that their situation is understood. I was asking the family member what else do the family wish from the hospital besides pain management or the medical care. And then the family member became tearful and said ‘everything is fine and we are happy with what is done. We only wish that our situation is understood better’ and was saying that for the mother who is witnessing her son in such a pain finds it very difficult to ask for a painkiller from a nurse. It is not that we are blaming anyone but it is a discussion here and that was very, very difficult for the family member (patient's sister) to express. We were behind the closed door yesterday and the family member expressed that her mother cannot witness her 24 year old son dying in pain and at the same time she (the mother) cannot ask the nurses for pain killer. That was the only wish expressed otherwise the family was happy with the care provided from the hospital.**

**Now we are coming to the end. And to summarise, we discussed about experiences and challenges, we discussed about drugs, we discussed about families, umm…some policy issues also came into picture. Is there anything else that you feel that it was important to discuss but we haven’t?**

General surgeon:

Now, many of our patients who comes to us from JDWNRH as palliative care patients whose families are being told that ‘nothing can be done’ and they are sent to us for supportive management. The biggest challenge we face is that the patients and families do not have adequate knowledge or information about the disease process. They do not have adequate information, adequate knowledge on why the disease is not being able to treat or cured. And that keeps on lingering in the minds of the patient and families and they come here and what better things can be expected to do here? So at the initial stage if it is well explained when they reach here, ok, they are mentally satisfied, they got adequate information on why I am not being able to be cured or treated then things become easier. If the starting is not well done then quite challenging once they reach here. There is no one who doesn’t want to get cured. Everybody who is sick wants to get cured. But they should know why he/she is not being able to be cured.

Gynaecologist:

Regarding that we actually once discussed in Thimphu. I think I don’t know that it is culturally accepted that if one is suffering from cancer that I am not supposed to tell him/her directly. I have to tell the family members and family members never tells the person that he has the disease. And that patient will keep on suffering…*laughs*…The disease never gets cured he knows but he doesn’t know what it is. And I think it is understood that in the long run you will know that you have cancer because people also expect that I think. That’s why I don’t know why people don’t tell. Actually in the west they have a right to know what disease they have.

Anaesthesiologist:

But aah… the problem here is because looks like it is socially associated because they say that once they have this news, bad news, in fact they die faster… *laughs*…May be their assumptions but I don’t know it is only in our setting. Outside (other countries) they (patients) know, they are aware of what is happening.

General Surgeon:

I think we have to prepare them (patients) well.

Anaesthesiologist:

Right, prepare them well. Actually looking at our patients, generally speaking, our patients, most of them, are not prepared to get such bad news. That is what I feel.

Drungtsho:

And one thing is there is a risk that they might commit suicide. Because they might consider that anyway one is going to die, so why not today than to suffer so much. That can be one risk if we tell the truth to the patients.

Anaesthesiologist:

That is one risk, yes.

Gynaecologist:

I think this part is related to spirituality. Not that we should not care but.. there are patients with cancer who come to us from Thimphu (after being diagnosed at the national referral hospital ) asking what is their diagnosis. And I ask them ‘were you not told?’. Then I feel whether I should tell the patient or not. You know, I struggle in front of them, should I tell or not. We also hesitate.

Anaesthesiologist:

We also hesitate definitely.

**From palliative care perspective I think it says that we should see whether the patient is ready to hear the news or not.**

Anaesthesiologist:

I think we need to consider that.

**If the patient is not really interested or maybe he would want his son/daughter to know, that’s different. But there are some patients who really want to know. So if they really want to know, then discuss with the family members and gradually may be with everyone's consensus they can be told.**

Anaesthesiologist:

So not generalizing in all the cases

**No, not at all. Never generalize. PC is said to be individual patient- and- family- focussed**

**Sister (Nurse Incharge, Medical ward) has something to say.**

**Nurse Incharge, Medical ward**:

Last year we had a patient who was Hepatitis B positive. And the patient was curious and kept asking ‘how is my report, what does the report say?’ Then I told her that she has Hepatitis B. I was then told that she actually was Hepatitis B positive since five years ago and the husband knew about it but he never wanted his wife to know and he had hidden the report from his wife. He told me he did that because his wife is psychologically very weak and often goes into depression due to her poor health. But I didn’t know about all those and so when she asked I just told what was there in the report and unfortunately her husband was not there. And later, you know, when the husband told me about all those I nearly went into depression myself for having told the truth to the patient.. *laughs*…

**That happens, isn’t it? I think as Sir (General surgeon) mentioned we have to prepare the patient and really prepare everyone, the family as well. It is said that it has to be at the right time, with the right people around, by the right person you know. It is complex I know.**

**Any one has anything to add or is there anything that you wanted to discuss and we haven’t done yet?**

**OK. So before we conclude do you have any specific suggestions, comments or advice, you know something that I can take forward because you are the ground reality, you are seeing patients with advanced illness and their families and you know the challenges. Is there anything pertinent that I need to carry forward?**

General surgeon:

Aah… I have reflected in the survey questionnaire, now at some point of time I think we have to come up with good team of palliative care and end-of-life support. And this care is going to be intensive, time consuming, long duration. The financial aspect is also going to be a big burden. With the experiences of past sixteen years whatever team or whatever number of medical professionals you train or put into place if he/she is not really motivated to do his duty, to move forward can be a challenge. If we do not have adequate resources just by putting a team in place cannot move forward. It is going to be resource consuming, intensive and challenging…*laughs*… So Ministry of Health should really be made aware and committed that OK the ministry of Health is also 100% committed to initiate and put in place some form of palliative care for the benefit of our patients. (Educate relevant people in the MoH and the policy makers on the importance of PC)

**Thank you Sir. That’s very important.**

Anaesthesiologist:

We need to have a separate program at the ministry of health who is dedicated in developing the module and whatever foundation to be provided. You might provide all kinds of recommendations but if it is not coming from the head up then it won’t be so … we need to have a dedicated unit, a program will be required. Even if it is not initially possible but gradually there should be an option to expand then that will be a biggest achievement from your part. Because till now hardly anyone in the ministry knows about palliative care. So if there is a program created and keep space to expand gradually that will be the biggest achievement.

General surgeon:

There is something like what the Health Minister said on the Nurses’ Day, the International Nurses’ Day, one of the four things that she said is developing a Nursing and Allied Division. So in the division it can really be looked into because I also shared with my colleagues that by virtue of our profession we are also involved in providing patient care but for 20 to 24 hours the patients are with the nurses. 70 to 80% of patient care depends on the nurses. I share during meetings also that they are the most important people. So by initiating this palliative care services and end-of-life support, unless we have those group of people who are motivated, dedicated …because at the end of the day we have to rely on this group of people. 60- 70% we have to rely on these people. Whatever the care processes you initiate in the hospital we have to depend on them (the nurses). So with this Nursing and Allied Division which is very important, some 1400 hundred nurses that we have and out of some 4000 Ministry of Health staff, it is a good thing. So with that division coming up more will be looking at the welfare and benefits of those group of people who deserves more than some group of people among our Ministry and from other ministries also. If there is no one to clean the urine and shit, not getting bath and patients lying there in the bed for months and months, nothing is done with their hygiene, what care are we giving? What care are we giving? (*repeats*) Everybody can walk in and give Paracetamol, dilute an injection and give it. If you train for ten days one can give Paracetamol, and injection. I tell that if I bring one security guard and teach him how to do appendisectomy ten times he will come and do it. He will come and do the appendisectomy. Somebody can put urinary catheter, even if he is not a nurse or a doctor, somebody can put the urinary catheter. So it is very difficult these days unless….

**It is a very, very important point la. I read about the Minister’s address as well.**

General surgeon:

Well, I think time has changed and I think nurses are also running away to developed countries like Australia for better income. I really feel nurses are depressed because the profession demands all kinds of things to do

**I think what sir has mentioned is a very important point.**

Nurse In-charge, Medical Ward:

Why we are not cleaning the patient is sometimes even if there is an attendant do you…are we supposed to do? I think we should involve patient party, care givers because one day this patient will go home and they will have to take care of this patient. They should be cleaning the patient’s stool and urine.

**Now from a palliative care perspective, we can train the family members by involving them while in the hospital so that they can do it when the patient is taken back home. In Kerala I saw family members doing wound dressing, some used to do it so nicely. It was taught by the palliative care nurses and they have learned how to prepare normal saline at home, they are taught to innovate resources and they do wound dressing using home prepared normal saline. And they do almost all the nursing care except for procedures like changing of urinary catheter where the palliative care nurse do it during home visit. So it is not always that nurses have to do but we have to show the interest to teach the family members how to do all those. Some of our patients and families do not know the imporatnce of hygiene. We all know that. Then even the family members, if we had shown genuine interest to care for their patients, I don’t think that they will not want to learn. In many other countries patient’s families are not allowed to stay throughout with the patients in the ward and so everything has to be done by nurses. Of course they have handful of patients but in our context family members are allowed to stay beside the patient so we can involve them and teach them in care giving. Palliative care is involving family members and I am sure they will do if they are really motivated and taught how to do. If we are committed and interested to do for their patients they will naturally be committed and interested to do for their own patients. They will feel like to take care of their patients.**

General surgeon:

Yes, yes.

Nurse in-charge, medical ward:

We do have such family members, the father of one of the patients, does everything.

**So do we have anything pertinent to discuss before we close la?**

Nurse in-charge, medical ward:

What is the status of palliative care in JDWNRH?

**In JDWNRH, they have developed a group, palliative care team, with a doctor and there are few nurses. They do home visits in the nearby community in Thimphu. I could not meet the whole team during my brief stay in Thimphu this time but I will be meeting them and accompanying them in the home visits after I complete data collection in the districts. The nurses in the group have received 10-days training at Kerala and I was also told that the team is being further trained by the group of palliative care experts from Singapore who came to the country and conducted workshops. I was told by Sister Yangden, who is the team leader, that the workshop was done module wise.**

**Anything else?**

**Nothing..**

**So if there is nothing more I would like to again really thank each one of you for the valuable information which will really be useful for the project.**

**Thank you very much.**
