# Supplementary material for: Healthcare professionals’ views on how palliative care should be delivered in Bhutan: A qualitative study
Source: PLOS Glob Public Health. 2022 Dec 12;2(12):e0000775. doi: 10.1371/journal.pgph.0000775 (PMC10021767; doi:10.1371/journal.pgph.0000775)
Supplement: S8 Data — (DOCX) [file pgph.0000775.s009.docx]

**Field Note FGD Mongar ERRH**

Date: 18.5.19

Focus group among health care providers in Eastern Regional Referral Hospital (ERRH) in Mongar was arranged on 18^th^ May, Saturday, since I was told that tomorrow on Sunday some of the relevant participants will be leaving for a workshop in Thimphu for a week. Hence, we (researcher and participants) discussed and the participants agreed to participate for the FGD even on Saturday afternoon. The time for FGD was decided to be at 12. 30 PM after the participants cleared their OPD and other activities. The venue for the discussion was in the conference hall of the hospital and it had round table sitting arrangement with minimal disturbances and there was no interference during the discussion. A simple lunch was arranged for the participants and following the lunch we started the discussion.

The participant consisted of an anaesthesiologist, general surgeon, gynaecologist, pharmacist, Drungtsho (Traditional physician), and nurse in-charges of dialysis unit and medical ward. The physiotherapist of the hospital was approached yesterday and he was willing to participate in the discussion but today he sent a verbal message through the pharmacist that he is not able to join the discussion.

It was one of the longest discussion so far and the discussion mainly prolonged due to the concern raised by one of the participants, the general surgeon, on the lack of basic care of patients in the hospital who are terminally ill and in general. Because I was also informally informed in Trashigang Hospital that nursing care there too is becoming very poor and because I was really concerned about it, today in the discussion since it was raised in the forum I thought we will discuss on it further because palliative care is all about giving care to improve the quality of life of the patients and families and it does require committed and compassionate caregivers like the nurses to meet the basic nursing needs of such patients and families.

It is understood that nursing care in general is deteriorating and that patients do not receive adequate nursing care. And as a nurse educator myself and currently a palliative care student I was really concerned because it was not only the lack of nursing care but the attitude of the nurses towards patients and their families that was raised. I really wanted to understand it thoroughly and so we had a thorough discussion.

Besides the issue on nursing care other participants like the pharmacist and the Drungtsho were also keenly interested in the discussion. All seven participants expressed their opinions and challenges in taking care of patients with advanced illness and some of them did express their interest in palliative care as well. The discussion lasted for one hour thirty five minutes fourteen seconds but the participants were still very proactive in the discussion. All of them expressed that palliative care is necessary and is timely to introduce in Bhutan.

Thank you.
