# Supplementary material for: Healthcare professionals’ views on how palliative care should be delivered in Bhutan: A qualitative study
Source: PLOS Glob Public Health. 2022 Dec 12;2(12):e0000775. doi: 10.1371/journal.pgph.0000775 (PMC10021767; doi:10.1371/journal.pgph.0000775)
Supplement: S9 Data — (DOCX) [file pgph.0000775.s010.docx]

**Field note on FGD with HCPs in CRRH, Gelephu**

FGD was planned for 7^th^ May at 1 PM. I was suggested to arrange a simple lunch for the FGD participants instead of tea and snacks as the participants would be hungry and exhausted after a busy morning and will be least interested to participate which I agreed and arranged accordingly. Prior to the FGD there was an ad hoc meeting in the hospital where some of FGD participants were involved and that meeting continued till 2 PM. So soon after that meeting, the participants were first provided with lunch which really boosted their energy and we commenced the discussion by 2.30 PM.

There were eight participants comprising of pharmacist, Drungtsho (Traditional Physician), general surgeon, ICU incharge (Nurse), Gynecologist, Gynae Ward Incharge (Nurse), Dialysis unit Incharge (Nurse) and Ortho surgeon. The group however consisted of only one female. Somehow I could not include the physiotherapist even after making two attempts to approach him.

I was assisted by one of my friends working in CRRH who helped me with note taking and other logistics. The venue was a small cozy reading room inside the library of the hospital where there was no disturbance and interruptions. The hall already had a permanent semi-circle table which was very comfortable and convenient.

The participants were very happy with the lunch provided prior to the discussion and they were all energized, focussed and everyone participated in the discussion. I ensured that everyone got a chance to speak and identified who talked more and also who talked less.

There were no one who dominated the discussion although doctors spoke more on the subject than the nurses which is usually the trend in Bhutan but I ensured that nurses also spoke and participated in the discussion. The orthopaedic surgeon had to leave in between the discussion because there was an emergency call. However, there was no disturbance or any interference in the discussion. It was very interesting that the Drungtsho, the traditional physician, in CRRH spoke fluent English and he also recently graduated with Masters in Public Health which is unusual for a Drungtsho. He had some exposure to palliative care experience while undergoing MPH in Bangladesh and was keen on the subject. The discussion was mainly in English although we also used Dzongkha occasionally. Everyone seemed to be very interested in the subject, Palliative Care, and they were all very encouraged. The discussion was very fruitful and satisfactory which lasted for 1 hour 6 minutes and 20 seconds.

Thank You
