# Supplementary material for: Healthcare professionals’ views on how palliative care should be delivered in Bhutan: A qualitative study
Source: PLOS Glob Public Health. 2022 Dec 12;2(12):e0000775. doi: 10.1371/journal.pgph.0000775 (PMC10021767; doi:10.1371/journal.pgph.0000775)
Supplement: S10 Data — (DOCX) [file pgph.0000775.s011.docx]

**Transcript of FGD with HCP at CRRH, Gelephu on 7.5.2019**

| Participant A | Pharmacist |
| --- | --- |
| Participant B | Drungtsho |
| Participant C | General surgeon |
| Participant D | Nurse in-charge, ICU |
| Participant E | Gynecologist |
| Participant F | Nurse in-charge, Gynae Ward |
| Participant G | Nurse in-charge, Dialysis Unit |
| Participant H | Ortho surgeon |

**Good afternoon everyone. Thank you very much for sparing your time to attend to this discussion. Can we start by discussing about your experiences in taking care of patients with advanced illness like advanced cancer or other chronic diseases like lung failure, liver failure, or gynaecological cancers where the prospect of cure is limited?**

**Gynaecologist:**

First I would like to share my experience. Aaah..I had an experience of working with chronically ill patients since the time I was a general medical officer that was in 2012. And then even during my post-graduation we had come across lot of women with cervical cancer, ovarian cancer and vulvar cancer, all the gynaecological related cancers. And they had to undergo major surgeries which is quite devastating to the patients as well as to the family both in terms of psychological, social, mental and economical, it is quite devastating. At the end of the day, it is most of the time not curative in intention. They need lot of therapy - radiotherapy, chemotherapy but at the end they die… after lot of struggling. And luckily in our institution (in Bangladesh where he did his post-graduation) we had palliative care centre. We have undergone basic course (in palliative care) as well and we used to refer the patients there. And life becomes totally different at palliative care centre. The patients are managed by palliative care specialist supported by the dedicated nurse, social workers, volunteers, spiritual leaders. There they (patients) know the reality. When they are in the medical ward we do not directly talk about death, we hide the death, we sort of give false promises to the patient saying that you will be alright and we are doing everything for you. But when the patient gets into the palliative care centre the approach towards patient is different. They make the patient understand that death is inevitable. You will die one day but till you die we are going to give a dignified life, painless life, struggle- less life. If you can’t eat we can give through IV fluid. If you can eat we can give you the best food you want. And the patient party as well is being counselled. So from that experience I was thinking Bhutan should have a palliative care institution as well as a geriatric team, the old age home. So this story is very important but at the moment we are discussing about palliative care in Bhutan. So from gynaecological perspective I find it so important and it will definitely apply to other clinical field as well like chronic illnesses, everything la. So my experience says palliative care, not only in Bhutan but throughout the world, to be holistic in nature. Palliative care is one centre where holistic care can be given in the same place till he/she (patient) dies and palliative care continues after death as well – the bereavement counselling to the family members.

**General surgeon:**

Aaah… as per my experience, the pain, pain itself aah the types of pain, you know, not only terminally ill patients because of the cancer, even in post op (operative) you know the initial post op day 1, 2, 3..they have lot of pain and the knowledge that we have (on pain management) is very limited. The..the.. the training period that we had is very short (for pain management). We just had one chapter and that was it. But when you are dealing with the patient who has severe chronic pain it is different, like it is very difficult to tackle. You give all sort of analgesics – paracetamol, brufen, anything, it does not work. You give multiple dose does not work. Now some of the high level analgesics like codeine, morphine is there in limited stock. When patient needs it is sometimes not there. So these are the main challenges and these are some of the experiences I have come across. So if we have enough medicine in stock, have good experience, knowledge and skill, it may not be just oral and injectable sometimes even other form of pain management can be provided and that would be very helpful. And yes, as our colleague said having the team (PC team) is very, very important I think in our setting.

**What are some of the needs in the patients when they don’t have the prospect of cure for their disease?**

**Nurse in-charge, Gynae ward:**

It’s the supportive care that they need. Patients who are suffering from cancers or like terminal illness I feel that they really need supportive care from the care giver plus the nurses.

**What would you say brother (ICU In-charge)? You are there dealing day in and day out with patients who are dying?**

**ICU nurse in-charge:**

In this context can I narrate one story?

**Yes please**

**ICU nurse incharge:**

There was a young man. I think he was one to two years younger than me. He had an advanced cancer and had undergone bone surgery and had chemotherapy as well. There were so many issues with him including even rejection from the family. He was even taken to court. And after seeing these things what the patient experienced I feel that we should be educated in such care and should have knowledge so that we can help the patient. I think he might not have gone through such problems if we could provide social help, even the shelters, we could have set up shelter for him and he could have lived there if palliative care was instituted in this country. Although it is late for him, he is gone, but for the future patients it is important if we know about palliative care.

**Gynaecologist:**

If I can supplement on this, in my experience aah…I personally have some of my relatives with chronic illnesses - my friends’ parents and my own parents as well. So what I feel the main thing lacking in the society is the psychological support. That is the main thing and proper symptom control. There should be someone who can control the symptoms without fear and repercussion. And one thing is, because we are poor Bhutanese people, majority, and they cannot afford the transportation to travel from home to hospital. A chronically ill patient going in the taxi is inconvenient and they have tough time. And the main (primary) care givers are their family members and at the end of the day when they have to stay for years and years, like a paralysed patient, whom you know shall never wake up. The patient dies thousand times a day and relatives as well die. So what I feel is the relatives as well as the patient should be given some time to stay separately like if we have a palliative care centre let the patient come to you without anyone, stay for few days, get a lift, let the relatives miss him/her and the patient miss their relatives. Again let them re-join to let them have a better life. I think these things are lacking in our society. From my observation I found it like that.

**Ortho surgeon:**

Aaah…Yes, so I will talk from my field (orthopaedics), I mean from my specialized field. Say like we have as already mentioned by my colleague (Participant C, general surgeon) there are many types of pain. One is physical pain and the other one is mental pain right? So physical pain, if people have, we can give some like morphine where we can still control the pain but the mental pain say for example some road traffic accident. Patients comes with some high C spine injury, C3, C4 fracture dislocation. There the patients will have complete paralysis, alright, but (at the same time) he is very conscious. This person is lying there, we had such three or four cases when I was working in JDWNRH. So now the challenges are as Participant E said first thing is economy. Some patients are from very poor background. They cannot afford to stay in the hospital in Thimphu or Gelephu in the town for long duration. They would like to go back home. And second one is about the care givers. So the challenge is now we don’t have enough staff, enough nursing staff to give complete care to those kind of patients. Sometimes those patients are just lying because such a busy hospital with less staff. So we need more people to take care of that particular three or four people who are just lying there. They have life but they cannot move their bodies. Then after few days, because there is no proper care they start developing bed sores. Bed sores it stinks, it is really bad. If there was a proper care, I mean with adequate staff, patients would not have developed bed sore. And then once bed sore develops though the patient is still conscious but there is a risk, as someone says we have to give proper care before, we know that person is not going to survive. Bedsore ultimately will lead to infection and their life is counted. We have to take again to OT then again and do all these flaps up and down, debridement. Repeated wound debridement we have to put those rotational flaps, drain and all and there are not enough attendants to look after for say like drain care or like the private area parts, urine, stool. People are just lying like that with catheter and there are very limited staff (challenge). And again from the counselling side, I don’t think we have enough counsellors who comes and talk to the patient, give them emotional support, right?.. and then some, kind of, giving them some encouragement from any side, hospital side or from the ministry side. I think we have but that it is not effective.

**Thank you Sir. How about you Drungtsho. We know that Drungtshos are also involved in taking care of patients with advanced illness. What are some of your experiences and challenges?**

**Drungtsho:**

As a practitioner for the last nine years I came across few cases aah..those who have availed multiple services from our allopathic doctors and they even availed some services from abroad and finally, you know, because when patients are ill with long term illness and don’t find solutions then they look for alternatives. And through rumours or with little knowledge that they have or through their own past experiences they come to us (traditional medicine). And to be very frank we have some limitations. Of course, aah.. people may claim that traditional medicine is quite effective, of course yes, it is effective in some means but in terms of like, what you say, terminal cases we do not have such medicines. If we were in that much advanced stage I think we would love to have some magic bullet type of medicine that would either cure or where we can disconnect, sometimes we feel like disconnecting because they are undergoing through such a pain there is no solution at all and they do not receive their social support from their families and friends. And they are just expecting something from us but we do not have any solution. Only thing is I sometimes feel like having a euthanasia that would end up their life peacefully because anyway we are going to die so if we can silently or someway (other participants laughs), but socially and culturally it is not viable but it is my view.

**Umm…As you said that euthanasia is socially, culturally not viable in Bhutan I think for the moment we will not really discuss on euthanasia (everyone laughs). But now we are understanding that patients with advanced illnesses do get physical pain but patients also go through psychological pain and spiritual pain which is termed as ‘total pain’. Patients who were dying not only suffers physical pain but also psychological, social, spiritual distress and also the needs for the families were identified. So Dame Cicely Saunders who founded modern palliative care termed it as ‘total pain’. And now, even if you do not have a magic pill, do you see your role, as a traditional physician, in palliative care team. If so, what would be your role?**

**Drungtsho:**

I, as a practitioner in the Traditional medicine, I should say that I think I am not completely qualified to be the mentor or the core member for palliative care. But if I refer to some of my senior most and highly experienced Drungtshos those who had been working or been in the system for decades because they not only have medical theory but they also have equal theory and practical experience in Buddhism, religion. So socially Bhutanese people have too much faith in Buddhism so Drungtshos can have influence in that.

**OK. So you see yourself as a potential member of the team, is that right? You do see yourself helping patients psychologically, spiritually, which could be a boon to PC team.**

**Drungtsho:**

Oh yes, definitely (Drungtshos can be a part of PC team)

**So let me try to summarize the first part of the discussion. So we mainly discussed on different aspects of pain in a patient who is terminally ill and then how we can play a role there. We also discussed briefly about the challenges we have today on pain management and other aspects of pain where psychological support is required, financial issues, lack of manpower, and other issues like bedsores, which deteriorates further the quality of life of patients, right? And also on the role of traditional physician and traditional medicine in palliative care. Do you have any questions here?**

**(No question)**

**Now can we discuss about the drugs? As a pharmacist in this hospital, today what is your experience? Do we have adequate drugs to manage moderate to severe pain for our patients who are terminally ill? What is your experience?**

**Pharmacist:**

As per my experience working in this hospital for the last one year and four months aah… I think aah..in the past there were issues with morphine supply. …… we used to buy the medicines from the open markets mainly India and Bangladesh. I think the quantity requested for morphine supply was very less. So that’s why the suppliers were not very happy to supply because they will run in loss. That was the problem in the past. In this 2018 – 19 I think we received the supply and currently we have morphine both tablets and injections and we also have codeine in stock.

**So you mean you have enough stock to help patients at any point of time?**

**Pharmacist:**

Aah.. I think aah.. right now we have it in stock but even if we run out of stock we can apply for mobilization from the national referral hospital and from other hospitals where the medicine is underutilized.

**In 2016 I attended a 6-weeks training on palliative care in Kerala and understood that morphine is an effective opioid to treat moderate to severe pain. After coming back I was curious, you know, I wanted to know whether we had morphine in our hospitals. I knew that there is morphine in the JDWNRH (national referral hospital), the regional referral hospitals and the district hospitals but it was hardly used and the drugs often got expired. And that was because many physicians, because they didn’t learn about palliative care and pain management during their medical education, many physicians not only in Bhutan but elsewhere as well, they feared prescribing morphine due to the fear of addiction. But now Bhutan is slowly considering palliative care and people are given education in the form of workshops and short trainings, therefore, physicians have started prescribing. So today there is not enough stock ,drugs are getting exhausted, patients are prescribed but next time when they come back for fill up there is no morphine and these are some of the issues.**

**As a pharmacist, from your experience, what do you say about the current opioid regulations? Do you think that our narcotic regulations are OK to ensure adequate supply of opioids, especially morphine, or do you think that the regulation needs to be reviewed and revised for adequate access to opioids for patients in need?**

**Pharmacist:**

Aah.. firstly for information in this EDL (essential drug list) review we have added sustained release morphine tablets mainly for palliative care. And I think the current regulation is OK because there is a strict regulation for CD drugs, controlled drugs, but that doesn’t mean that access to the patients is denied. They are doing that just to prevent abuse by the general public and in the hospital by the staff. They are doing the strict regulation mainly to control the abuse but not to deny access to the patients.

**Which means the regulation is actually supportive for the needs of patients?**

**Pharmacist:**

Yes. Last Saturday one of the senior pharmacists from the narcotic agency came here and she was talking about it. Actually they are not controlling narcotic drugs for the patients, when they are prescribed, they are strict only to control abuses and misuses.

**Gynaecologist:**

If I am allowed to talk for one to two minutes.

**Yes please.**

**Gynaecologist:**

I think we are focussing more on the morphine and opioid related drugs. But palliative care needs more than opioid group of drugs. Patients with other symptoms - infections, dehydration, pain control other than this. With morphine the laxatives, I think everything is required and have to have all these in the supply and also like especially when we talk about palliative care mostly the patients are with cancer and if they are bleeding or something other than radiotherapy we cannot stop it. Radiotherapy facility, everything has to come together and some mass which is obstructing, intestinal obstruction, surgical team. So I think we have to talk in as a wholesome. So we are focussing more on morphine and opioid group of drugs.

**Ortho surgeon:**

Regarding morphine whether it is available or not, I think morphine is one of the main drugs that we use in palliative care, right? So from my experience, I don’t know whether surgeons maybe using it sometimes and gynae I don’t know whether they use or not , but I think in the hospital I am the one who use morphine frequently for my post operative cases. I always advise morphine 4mg IV stat and then SOS 6 hourly or 8 hourly because we have to give big incisions which goes down to the bone and we have to fix the bone and come back and you give multiple layers of sutures. So patients especially on post op day one and day zero it is very painful. So we always give morphine. So last time in 2017 or 2018 I don’t remember but once it was out of stock.

**Pharmacist:**

2018

**Ortho surgeon:**

So my patients are not terminally ill but if you need for the terminally ill patients we need to have it in stock in the pharmacy department or BNCA or DRA. I think we should have adequate number of vials so as to treat this cases. I think I am the one who uses mostly. This time I think it is there in stock.

**I think our pharmacist Sir here assures us that we do have enough in stock at the moment and the regulation does support us, right sir?**

**And as Sir (Gynaecologist) mentioned there are so many issues with palliative care patients, so many drugs and so many resources required. But one of the pertinent issues, especially in the developing countries, is not adequately utilizing pain medicines like morphine which is very cheap, which is affordable and is very effective. As per the literature, you know, developed countries consume more than 90% of the total morphine produced, way more than what is actually required for them. Whereas, majority of the patients with advanced cancer and other life limiting illnesses are in the developing countries and it said that we just take 5 - 10% of morphine. So in Bhutan we are beginning to understand the importance of palliative care, and we are trying to introduce palliative care and integrate it into our health system. So it is also important to understand drug issues and other aspects and also understand the regulations so that, as mentioned by our pharmacist, we do not misuse. You know the regulation has to ensure that the drug is not misused but also ensure that it is adequately available and used for the patients who needs it.**

**Pharmacist:**

It is also important to know about documentation when we use controlled drugs. We mainly focus on this. We used to go and check the documentation of controlled drugs once a week but sometimes we cannot go due to staff shortage where we do check once a month.

**Gynaecologist:**

Madam (researcher) was talking about the prevalence of morphine use comparing the developed and developing countries. The reason I feel why it is least used in the developing countries is because one is regulations from the regulatory authority. Because they (regulators) themselves lack the idea on morphine, the pharmacology of morphine. So in that way that is number one. And number two is the prescribers lack the knowledge. We ourselves are afraid of prescribing. We think this is addictive drug, the patient will end up with respiratory depression if we use it which actually is not true if we know how to use it. And in reality morphine is a much better drug than pethidine but I myself am more comfortable using pethidine because I have been using it. Even I know the pharmacology from A-Z about morphine I still continue to use pethidine because it has become culturally acceptable in the ward. And if one day I start using morphine all my staff will open their eyes and wander ‘new prescription, new people, new changes’. It is difficult to change the culture. And so I think there is a role of education and awareness in everything. Morphine I would say is the safest pain killer analgesics. Pethidine has a cumulative effect, the more you use the side effect becomes worse and worse, but morphine when you use in the terminally ill patients they say it is not addictive but patient develops tolerance that means the dose needs to be increased. That is the only problem. But it being a very cheap drug increasing the dose doesn’t matter and even if the patient gets addicted when they are about to die there is no harm in getting addiction we know they are going to die soon. Let them be addicted and die peacefully under addiction. I think it should be freely available with a controlled register book.

**Drungtsho:**

If I may supplement on this aah.. along with the drugs that we are discussing here, if we can offer, from the traditional medicine side where we have spa, massage, traditional massage, and we have herbal bath, acupuncture and we have meditation and very soon we are coming up with yoga and meditation. So this will be a psychological and both spiritual healing practices. So it can be like a holistic approach. Only thing is we need to collaborate. We need to carry out some need assessment jointly and if we as a package, complete package, if we can offer to the patient I think it will be very much beneficial.

**So has all these started in the traditional medicine?**

**Drungtsho:**

Yes, yes.

**I know there used to be steam therapy and acupuncture but...**

**Drungtsho:**

Yoga is recently started. So yoga is another, yoga has different branches, the..the….the..the branch that we practice in our traditional medicine is little bit different which will be much more suitable for the patients. I also got trained recently in meditation and the ministry has now ordered us to implement these services here in Gelephu hospital.

**Ortho surgeon:**

I have few things to add up there la. We always talk about the patients and then we forget to take care of the care givers. We always treat the patients but we forget to treat the care giver who is already so much traumatized because of caring for the patient. They have so much stress and we always forget to care about for the caregivers, to take care of the care givers. We always try to say in the ward also attendants are asked to sit in the chairs but they don’t have a proper place to rest.

**General surgeon:**

I think we are deviating from the main topic that we are discussing right now.

**Ortho surgeon:**

No sir, but palliative care means involving care givers as well.

**General surgeon:**

I think we will come to that later

**Ortho surgeon:**

O OK OK, because I thought we will miss this that’s why. We should take care of patients together with the caregiver for maybe better outcome.

**Actually we are coming to caregivers or taking care of family members after this. So do we have anything more to discuss on drugs la. *No response*…. So to summarise on drugs, we understood that we now have an increased stock of essential drugs like morphine at least in the regional referral hospital.**

**Gynaecologist:**

In this regard I would like to make a comment here. Making these important drugs available only in the central regional referral hospital and not making available in the districts and BHUs we are creating more problems. It is the same Bhutanese people getting treatment from the same MBBS doctors everywhere and I think it should be available everywhere. The poor patients who live in the villages cannot come just to get morphine. It is not that the doctor can’t prescribe it is just because of the system and culture it is made unavailable. I think this barrier should not be there. Whatever drugs that are available in the country should be made available to all the prescriber wherever it may be. Like some anti-hypertensive, some people who are at the liberty of getting good drugs, who can afford can easily get it. Those poor people who can’t afford are made more difficult by restricting the availability of drugs. I think it should not be like that and there should be a system change.

**That’s where our regulations comes into play.**

**Gynaecologist:**

Yes

**Pharmacist:**

I want to add something on this la.

Actually I think we can’t make all the drugs available in all the health centres la. That’s why we have a form based medicines la. Aah.. BHU can access the drugs from BHU Grade I on a form basis. The access is not denied but there is separate channel for accessing the drug.

**So if the BHU staff need morphine they can still fill form II and get it, right? And in the long run as we start educating our health care professionals and our public and once palliative care service starts integrating into the health system I am sure the policies need to be changed, you know, and then pain management like morphine may have to come down to the BHU level with adequate training of the health assistants because as Sir (Gynaecologist) said patients at the grassroots level purposely need to come to JDW or the regional referral hospital only to get the pain medications which is often challenging as they are already in pain and distress. So let’s hope that this improves as PC develops. This should support in reviewing the policies, you know.**

**So basically we are understanding that we do have increased stock of morphine. Physicians are now getting educated and prescribing it. It is important for palliative care drugs to come down to the grassroots level, right?**

**Now let us discuss about family members and care givers as Sir (ortho surgeon) was concerned and identified how important it is. In palliative care families are equally important as patients. We cannot take care of patients unless we take care of the family and as a physician and as a palliative care team if we do not focus on family members we will not be able to focus on the patients. So can we discuss about taking care of family members?**

**General surgeon:**

Dealing with the family is very difficult because patient is chronically ill for months or even years. Aah..Once you counsel them (family members) on the nature of the disease and how long s/he may survive, and the anticipated problems, the problem is they (family members) are bogged down with that task given to them, you know, everything they have to do to take care of the patient. Another is financial support. One is follow up. Now follow up is the most difficult thing in our country actually because one thing is due to financial constraints, other is because of the geography, then monsoon season where there is rampant road blocks. So repeated follow up is very difficult because they don’t have financial support from anywhere because the family is very small and sometimes you ask them they have only one or two people in the family. Whether that person is going to take care of the patient or work in the field. So they have all those difficulties.

The other thing is now our people in the village listen more to the spiritual leaders. They sometimes completely block them (patients) aah…telling that, you know, you cannot go to this direction on this day or you cannot go and have surgery on this day and if you have it you will die. All these taboos in the family, in the society, you know. Those are the challenges to manage when it comes to taking care of the family.

**Gynaecologist:**

Aah..in my experience and my opinion to manage a family we need time and resources. So the main reason why we are not able to give a good family support is because we lack time and resources. Time in the sense I, as a gynaecologist, I don’t or won’t be seeing only one patient. For me that terminally ill patient I know is important but my concentration will be more towards the other patients whom I know will go back home if I don’t take care. And to manage the patients with advanced illness we need to know their family very well. Without knowing we cannot give blind therapy. If you try to give blind therapy we are adding fuel to the fire and it will make the family situation worse. So it is sometimes better not to poke our nose without knowing the real condition. So in that sense with us we need social workers, volunteers who will actually visit the home and see the surrounding conditions and they will come to us and tell, and they will take pictures, these are the home conditions, like this and like that. Then we have to understand the situation and from there we can plan the management. So I think not giving family treatment in our setting without knowing the real situation will be better than giving blind therapy. We will be making the things worse.

**Drungtsho:**

From my research perspective and from the management side, we, as primary care givers, it is our sole responsibility to reach out our services to the community. So if we do not know our overall social context and the behaviour of the community so then we are lacking. So now I think what model we, the ministry of health, is following I don’t know. It is the conventional way of practicing. All the specialists, doctors and nurses are confined in one room and always day and night they come and practice. So it is very limited for them to get engaged with the community. So from my perspective I think it is very important that our doctors and nurses to familiarize with the community with the people from time to time. They can visit their homes, their villages so then they can try to analyse and understand what sort of behaviour, their dietary pattern, and the way of their living and livelihood. So from there onwards they can understand the context. We will have close contact with the community. Then the real practice, clinical, social or any therapeutic practices will begin that will be much more valuable and that’s what I perceive perhaps. So if we blindly go like what participant E (Gynaecologist) pointed I think it is very difficult. Because people perceive in different ways and as participant C (the surgeon) said some people are now lost in their own olden practice, say they are talking about *zakar* (suitable dates) when the patient is almost dying. So our doctors and what to say our OT team is waiting for them but somehow the Lamas (spiritual person) insists so that postpones which may lead to complications. So these are main social problems that we need to educate them and as I strongly propose because it is very important for our doctors and nurses to get more engaged with the community.

**At the moment we don’t have social workers in the hospitals. In palliative care social workers are an important member of the team. Social workers can help patient and the family. So basically this discussion is helping us understand that palliative care will also need social workers, who will help support social issues.**

**Drungtsho:**

And we need to introduce family doctors also.

**Gynaecologist:**

And we need to create awareness among spiritual leaders, local healers and local leaders who will play big roles in palliative care.

**Our next point is about how we should create awareness on palliative care. How should we start creating awareness to our health care fraternity, to our general public, to our policy makers, to the and spiritual leaders and traditional healers? How do you think should we go about?**

**Gynaecologist:**

I think this is easy to discuss but difficult to achieve. Actually there are many ways of reaching at the level, mainly through discussions, and education in the public forum, media, social media. Other one is from home to home, home contact, then making posters. It is very easy to discuss. I think what we have to do is there are different groups of health care workers. Some are already in service, some are still undergoing training. But for those who undergoing training those people are easy to catch. You can inculcate a module of palliative care for those who are undergoing training, like nursing training, those who are doing post graduate medical training. If we have a palliative care module and they should be made compulsory to pass that module. They can’t escape. In that way next generation of health care workers will definitely know. Now the problem is with those who are in service, in Bhutan especially, because huge budget is needed to create an awareness. So for this I think we should have palliative care guidelines suitable for the Bhutanese setting. So a group of people should get together. Not only medical professionals but spiritual leaders, volunteers, social workers, all the specialists. Specialties involve in the care of terminally ill patients and we have to develop a guidelines. That guideline has to go from centre to centre. We have to train a trainer who goes from one centre and trains a group of trainers there. So in that way that is the only option to train the health care workers. And once we get to the health care workers to get to the public, best place is to catch the students. Go to the schools give a talk, talk with teachers. So once the students goes to the villages definitely they will spread this story to their family. So we don’t need to do many things but if you catch the critical people at the critical time at the right place things should work. And the things we talk in school should be different from things we talk in health care centres. It is two different stage, the strategies should be there.

**General surgeon:**

Another thing is I think we already have counsellors, especially in the hospitals, psychiatrist, all these people. I think we can give them some training about the counselling.

**Drungtsho:**

And related to the budget. Royal Government of Bhutan when they are allocating budget sector wise I think the Ministry of Health receives very minimal. So as per the WHO standard, the international standard, the health sector should at least book 13 something percent of the total national budget, for public health budget. But till now we are receiving only five to six percent which is very minimum. That is the main gap we cannot bring about all those noble ideas. Like if we want to really, you know, advocate and make aware all that. Because we are very handful of staff in health ministry, around 3000 health care workers across the country. That’s not so much of issue if the political commitment is there. So if they can give more money for the health sector, if they prioritise, I think it should not be the issue. That’s what I feel from my side. And even during last national health conference I voiced and it was reflected in the *Kuensel* (national newspaper). I fought for the budget and Lyonpo (Minister) was laughing. He says every ministry is equally important. Why don’t we keep our health ministry, you know, few steps ahead from other.

**Gynaecologist:**

Surprisingly we have a free health care system with minimum budget…*laughs*…

**Very important information. Budget is the most important issue. Whatever we plan depends on the budget available.**

**Now a summarising question, do you have any particular suggestions, advice, or any other specific comments on the project? You have read the participant information form and you know what the aims and objectives are for this project. I will be happy to take your advice or suggestions because you are the ground reality, you are working here.**

**General surgeon:**

I think not only about coming out with papers and presenting your findings, aah.. wherever you are getting trained I think if they also could support some of our colleagues, staff in training how to give care to the terminally ill patients, how to give care, how to alleviate pain, any interventions that is required and support us in setting up because only you coming here with those knowledge also is I think difficult to build up the whole system. I think if we have multiple aah..characters, you know, it is a multispecialty approach. Even psychiatrist also has to be involved not just the surgeons, not only the counsellors, even nurses. So how to build up those things. Some of us could be trained I think, help us in coming out with.

**Very true. And one of my focus when I am abroad is networking you know. There are organizations who are interested in facilitating palliative care in the developing countries. There are organizations to help introduce palliative care in developing countries like India. Recently there was a group from ECU (Edith Cowan University) in Perth visiting Bhutan. And someone in the group had expertise in palliative care and fortunately I happened to be in the country. We organised to meet and we invited the team leader of the palliative care home care group in JDWNRH. People are interested to provide training where many can be trained. For individual specialization, I would really encourage the person to explore online because these days it is to do so much online and if there are people who are interested, you know, I would be able to facilitate but the passion and the drive has to come from the individual and there are many avenues I am sure. And we can bring the concept to the country. I agree that as an individual it is a big challenge and the opportunities can be given to others. I do agree sir and thank you for that.**

**Gynaecologist:**

So my opinion is, at the end of your study, I think your ultimate aim is to start a palliative care centre here in Bhutan so with this I don’t know whether it is included in your study or not, so by the time you pass out and you are ready to open an institution with no manpower I think it is not going to work. So in the meantime I think you will have some background whether the appropriate manpower is available and amongst them the most important is the palliative care specialists which is a different entity and in the meantime before starting the institution in an empty building I think you have to have at least one specialist to cater to our population of around 700,000 where around 100,000 people may need palliative care. And every society, every nation, the patient may be having the same disease but the needs will be totally different. So I think there has to be study on what particular thing is needed for our Bhutanese setting. Our Bhutanese people may not need morphine but they may need someone to stay together, our local healer to give blessing that may reduce the pain. So we cannot directly adopt the international standards. I think we have to have our own study and it has to be suitable to our society. And in the meantime if we do not have all these data, at the end of the day, you cannot start in the empty house. The empty house will be filled by the ghost and dead bodies.

**General surgeon**:

Ya, that’s what I also feel very important by the time, now you have three more years, by the time you come back, because by profession you are a nurse, right? You cannot prescribe medicines. So if you do not have somebody who can, you know, aah..who can prescribe. Pain killer is not only giving injections, you know, we can put wires and stimulate, you can hire those techniques, skills here I think it will function successfully. That’s what I feel. So I really think some specialists should get training and come with you together.

**Actually that is what I wish too. To have at least one palliative care specialist.**

**I am an academician and project will help bring the concept of palliative care to the country. I can educate students and staff on palliative care. I did mention to the Health Minister about sending doctors, passionate doctors, not just any one because palliative care is so much to do with your own attitude and passion and if you don’t, as Sir (gynaecologist) mentioned, if you don’t have passion it is very, very difficult. So we were also talking about recruiting interested doctors, especially for palliative care course. Another thing is in JDWNRH there is a home care PC team at the moment and there is a doctor in the team who received short PC training. So hopefully he/she will pursue further to specialise on palliative care. Because there is so much difference in having undergone palliative care training and not having done the training. Morphine prescription or any drug prescription in that case becomes very different. So I did raise this point on training doctors for palliative care specialists. In Thimphu, the palliative care team have received training from a Singaporean Palliative Care team on principles of palliative care, pain management. They have completed two modules till now and they are left with few more modules. So what I can take back at the moment is training needs to be spread further and seriously emphasize for palliative care specialist.**

**Gynaecologist:**

If I can share my observation in Bangladesh. Bangladesh is a Muslim society and we think Muslims are cruel (inconsiderate) but it is not like that when it comes to their family members. They have a big sense of brotherhood. If his brother is going to die he can give his kidney right now. If his brother is losing the sight he is ready to give his one eye now immediately without anaesthesia. But if someone else is dying they won’t care. Let him die they will help them to die. So in that sense even a country like Bangladesh they have a palliative care centre. Initially they did not have a specialist in palliative care medicine. And as you said, a dedicated anaesthetist who was working as an ICU incharge, he took up the challenge and he went for a short course and he is now so powerful, big man, experienced man, people listen to what he says, he has a political affiliations. That is what is needed and with that he started a small palliative care project and slowly it has grown up to be a department of palliative care and every year they are producing a batches of doctors who have completed post graduate course in palliative care medicine. So I think if we have the dedication and will it is doable and we can do it. So only thing is we need dedication, motivation, incentives of course to do it.

**Drungtsho**:

Madam, can I add one thing?

**Yes, please**

**Participant B:**

Since here in Bhutan majority are practicing Buddhism, right, now the way of Buddhism is now different. People are commercialising and in any house if we perform some rituals it is becoming very expensive. And the concept and principle of Buddhism is to benefit all people and all beings. But it does not happen any more. All the Lamas , they drive land cruisers, they meditate in the caves or their palaces, whichever they choose to, but the core principle is not practiced. So now when it comes to or if I connect with the palliative care it gives the clear sense that it should be connected spiritually, right, from the spiritual point of view. So how about in your study if you can try to focus some area where those spiritual leaders can be the part of the palliative care. And yesterday as I pointed out I would suggest you to incorporate this hypnosis and mesmerism, very important in palliative care where they can change their mind set, they can, what to say, psychologically, you know, get lost into whatever current situation and they will not have time to, you know, engage in their pain and sufferings. So that’s my message.

**I was also reflecting on if after our discussion yesterday. To be honest I have not understood about hypnosis in palliative care but I will learn about it. I will explore about it and learn further on the spirituality because the model also emphasizes on spiritually appropriate palliative care for Bhutan. We are also involving spiritual leaders from the *Zhung Dratshang* (Central Monk Body) in the stakeholders group next year to help us include spiritual person in the team. Bhutan is predominantly a Buddhist country but we all know that there are also people with other religious faith, right? But because it is predominantly a Buddhist country we focused on Buddhism but then we should also have space for others, if we are giving palliative care we should have space for other communities. So spirituality is a very important point in this research and so I take it very positively and I will also explore about hypnosis and mesmerism. Thank you Drungtsho.**

**Drungtsho:**

I want to apologise because I did not mention about other religion but…

**No no it is understood, as I mentioned because Bhutan is predominantly a Buddhist country and I have also focussed on Buddhism but other religions are equally important and we must ensure the same respect and regard and ensure same dignity to all the patients and families diagnosed with terminal illness irrespective of what religion they follow.**

**Now is there anything pertinent that we didn’t discuss and you think is important to discuss here? I am sorry it is prolonging but it is also very important.**

**Gynaecologist:**

The most pertinent thing is we want to know the findings of your study when you publish it. *Many laughs*…

**Sure, that is assured. You may have to be patient though.**

**Gynaecologist:**

I am quite interested.

**Nurse, ICU in-charge:**

And reach palliative care service to the grassroots levels. If we focus and strengthen the service to the community then we can build relationship with the local leaders, healers and other community support individuals.

**Drungtsho:**

Also madam try to include the home care nursing or the community health nursing.

**Sure, community palliative care and home care are among the priorities. So thank you very much. Your opinions and your contributions is really going to be invaluable and it was beyond my expectation. This is my first focus group discussion for this project and I am very encouraged with your participation.**

**Thank you very much.**
