# Supplementary material for: Healthcare professionals’ views on how palliative care should be delivered in Bhutan: A qualitative study
Source: PLOS Glob Public Health. 2022 Dec 12;2(12):e0000775. doi: 10.1371/journal.pgph.0000775 (PMC10021767; doi:10.1371/journal.pgph.0000775)
Supplement: S11 Data — (DOCX) [file pgph.0000775.s012.docx]

**Transcript of FGD with HCPs at Gidakom Hospital on 24/7/2019**

| Participant 1 | Chief Medical Officer |
| --- | --- |
| Participant 2 | Pharmacist |
| Participant 3 | Physiotherapist |
| Participant 4 | Nurse in-charge, general ward |
| Participant 5 | Nurse in-charge, MDR-TB Ward |
| Participant 6 | Drungtsho |

**Good afternoon everyone. I am very grateful and thank you Sir (Chief Medical Officer [CMO]) and thank you everyone for agreeing to participate in this discussion this afternoon despite your very busy schedule.**

**My first question to the group is what motivated you to participate in this research? Why did you think it is important for you to participate in this discussion?**

Drungtsho:

Should I?

**Of course.**

Drungtsho:

As per what I have understood from the information form is that this is a useful project. It is relevant to our work. That’s why I thought it will be good to participate.

Nurse in-charge, MDR-TB Ward:

For me the concept of palliative care in MDR tuberculosis is new. Initially I thought palliative care is for terminally ill patients only like cancer and also liver cirrhosis or something like that. But yesterday when I saw madam interviewing our MDR patients I realised the concept of palliative care in MDR-TB, though I have been working in MDR for almost last five years. So it is a kind of new concept and it actually motivated me to know more to explore further. That’s the reason for my participation. (Wanted to learn more about PC)

**So you realised that palliative care maybe applicable to MDR-TB patients as well and you became curious to know what it isall about?**

Nurse in-charge, MDR-TB Ward:

Yes, and may be for the future we can know about the model of care and bring certain changes.

**That's good. Thank you**

Pharmacist:

Palliative care when it comes to pharmacy, I haven’t seen much role till now in Bhutan. And then when you have come up with such program and want to involve whole lot of staff for palliative care it motivated me. Because in Bhutan, pharmacy is seen like being at ‘behind the door’ you know. So that’s why if PC can include the role of pharmacy then I think the society will also know how pharmacy can also contribute in improving the patient’s health. And then especially in Gidakom which is the centre for MDR-TB in the country and everyone is working towards ending TB by 2030 and PC is helpful for MDR-TB patients when they have treatment side effects, to have patient compliance which is quite challenging. So I think if PC model comes to our setting it will help us in patient compliance and then I feel aah.. that to end TB by 2030 will be much more achievable.

**So you see that, as a pharmacist, you have a big role in taking care of patients with advanced illness, is that right?**

Pharmacist:

Yes

**Thank you**

Nurse in-charge, general ward:

For me I have been in the service for last ten years or so and here in Bhutan we do not have palliative care as a separate unit. Everyone is kept together, all mixed. So as madam is studying this subject, through your study, because the needs for PC patients are different than the needs for other patients. So even here if there is a kind of unit for PC. Even if there is no separate PC hospital its ok but if there are PC units within the hospital. An area for PC or even a PC corner so that we can keep such patients separately. I came to understand whether there can be any such options.

**Ok.. that’s interesting. Thank you**

Physiotherapist:

My main motivation is aah.. physiotherapy has been there in Bhutan since long time back but I feel it is still not very clear to people about the physiotherapy services in Bhutan. So the moment patients come to us you know what they say? They say that they just want a massage which is completely a wrong perception. So when it comes to PC there is a major role for us (physiotherapists) I feel. PC is most of the time for terminally ill patients who are bed ridden for long time. So when the patient is bed ridden for a long time there will be pressure sore. Our role is if we intervene there at least we can prevent the bed sore through various modalities and through positions. That’s one thing and as per our Bhutanese’ beliefs terminally ill patient means that there is not much hope and they are not let to move their limbs and the patient is just kept like that. But our concept is different where we actuality help such patients with mobility. So I see that our (Physiotherapist) role in PC is crucial. The past model was different. Now if this model is going to include us (Physiotherapist) in PC then we will also take the responsibility and we see our role crucial in PC. And for that I got motivated.

**That’s very encouraging. Thank you**

Chief Medical Officer (CMO):

Thank you for choosing us for your study. Welcome to Gidakom. Aah.. I think PC is a need of time. And then now that we have in terms of health we have worked quite far for primary health care and I think we are at a stage where we need to focus on PC. Basically we want our patients, the chronic patients and end-of-life patients to die with dignity and to die without pain . And then now that we have lot of chronic patients which includes like COPD patients, MDR patients who need to take medications for a long time and also who needs a long time follow-up. And apart from the increasing number of cancer patients and I think in our setup this is something we are lacking and even, as I mentioned in the survey questionnaire, in my last couple of years in service I hardly got any training in PC and I don’t remember anyone really mentioning about it anywhere giving us the importance and making us do whatever we can. So I think this is going to be a good discussion and then your study is going to be fantastic.

**Thank you Sir.**

**My second question is, what are your experiences in managing patients with advanced illness? And what were the challenges you faced?**

CMO:

Aah… we do encounter many such patients and basically in a facility like ours there is nothing much we can do except manage pain and send back home if the family wishes to take the patient home. Sometimes we feel that keeping them in the hospital will expose them to so many infections and they are already immunocompromised and our wards are crowded. And especially in Gidakom hospital we have lot of patients with cough and we have MDR-TB patients. So we feel that it is a high risk and we send them back with oral analgesics and ask them to review whenever possible.

Some of the issues (challenges) are because I think we don’t have a counsellor, a clinical counsellor, in the setting and definitely a counsellor is very important in managing and especially helping the family and relatives to provide support and care to the patient. And other than that we cannot make a home visit. It is very difficult and our policies doesn’t really support for that. The other reason is we already have shortage of staff in our hospital. The other could be because of the financial implications and therefore I think there is nothing much to do unless patient comes back to hospital. And I don’t know whether this is relevant to the question but our BHUs (Basic Health Units), we do have BHUs, small health facilities scattered all over the places but I wonder if they are really managing or if they can take care of such patients because they are nearer to the patients and they are accessible, available and they also know the families and the community, they should be knowing the families and communities.

**So you mean the health workers in the BHU could make a difference to the quality of life of the patients who are at their homes in the community?**

CMO:

Yes, because personally I have been seeing that most of our health care workers in the BHU setting, they are not very competent in carrying out nursing procedures like right from inserting IV lines and it is very difficult.

**So you mean, given an opportunity that they receive training on PC, they (health assistants) may be the right professionals to care for patients in the community?**

CMO:

Yes.

**That’s a very important point Sir because while PC can be given in the hospital as in-patient care, as out-patient, you know they can come to the hospital if they have severe pain and if they require admission due to any symptoms they can be managed in the ward but they can also come as OPD patients. Then PC services can also be provided at the district hospitals and the BHUs and as Sir mentioned it can also be reached at home. PC can be provided even at patient's home as home care service. PC professionals can go to patients’ home to provide care. So one area is, as Sir mentioned, making the BHU staff competent in taking care of patients at end-of-life, right?**

**How is your experience Drungtsho, when patients with advanced illnesses like cancer, which do not have cure, comes to you?**

Drungtsho:

Well, if I talk about my experience, it is been almost thirteen years that I have been serving and many such patients have come to me and I do have experiences with them. So when you say PC it is a kind of terminal care, right? And for that, although, we have not received any special trainings, even then we give an extra thought to do what is best for such patients. What these patients expects from us is mainly to clear their doubts through, you know, counselling on what medications to take and what to avoid (Information needs). And if the medications are not going to be helpful what other approaches to do. And one thing when we are ill it is the mind that we need to take care of. The primary cause of the illness is generally said to be the mind. And as the disease gets advanced if the mind is not taken care then it becomes difficult. So we help to take care of the mind through counselling. And to take care of the mind depends on what religion we believe in, you know. And since we are in the Buddhist country when we are ill with chronic illness it is not only the hospital (medical treatment) to be relied on. I am not sure how only hospital can manage but I know that in the communities we see people perform rituals and other traditional practices and when I see such patients in my clinic I give them those advices, you know, like a reminder that those practices may also help them.

**So you are saying that you, as a Drungtsho, do have a role in providing palliative care?**

Drungtsho:

Yes, we do.

**Thank you Drungtsho.**

**Now to you brother (nurse), as an in-charge of the MDR-TB ward, what is your experience so far in dealing with patients, I mean in managing patients with MDR -TB?**

Nurse in-charge, MDR-TB ward:

Ok madam. Like being a nurse sometimes when we care for terminally ill patients or those dying patients, sometimes aah…we have no choice but to only give psychological support or some family support like reassurance. But we are sometimes so helpless. Aah… in MDR ward, every year at least one or two patients expire. Especially those who have MDR-TB and other co-morbidities like ALD (Alcoholic liver disease), diabetes and also like gerontological diseases like the old age factors. As a nurse in such case we try to reassure the patient parties like the families and also to convince patients regarding their prognosis and also meet their needs like nutritional needs. We also want to ensure a peaceful death and also some kind of pain management as per the directives of physicians. That’s all I think. And also to a certain extent we also provide assistance for meeting the spiritual needs which is very important being in a Bhutanese context, in a Buddhist country. Especially for those dying patients, they seem to have a peaceful death if they have Lamas (Buddhist master) around to perform some prayers for them.

**Do you think those support that you have provided helped improve patients’ quality of life or the quality of death?**

Nurse in-charge, MDR-TB ward:

Yes but there are so many challenges we face like the lack of training as this is a new concept for us and for as I mentioned the concept of PC for MDR-TB is very new to us.

**When PC started initially it was mainly for patients with cancer you know, cancer patients who were dying. But today PC applies to all patients with advanced illness. PC is not only for dying and end-of-life patients and it also is not that PC starts only when there is no cure. PC can be provided to patients who are recently diagnosed with an advanced illness. For example, in a patient diagnosed with cancer you know, there will be so many issues with the patient and his/her family, right? It is not only the physical pain but then the psychological pain, the mental, the emotional, spiritual distress, all these things. So that’s why along with the therapeutic measures if PC is given side by side then it is found that the patient’s quality of life gets better and the treatment also goes well and then the survival is also found to be longer as per the evidence. So that’s why MDR-TB patients definitely requires PC as you have experienced about so many issues they go through when they are diagnosed and treated.**

Nurse in-charge, general ward:

If I have to say my experience it is like what the doctor and the Drungtsho have mentioned. Aah… the moment a patient is diagnosed with a terminal illness we cannot keep the patient in the hospital till they die. So once they are diagnosed they may be kept in the hospital for some time and do whatever is possible medically by the doctors and nurses and ultimately they have to be sent home. And when we send them home we have to tell the patient that the disease may not be curable and if we give them a tentative day for discharge and in the mean time we start giving them some counselling where we tell them that ‘it is not necessary to remain in the hospital as you will miss to carry out so many things that you have to do at home’ but if they face any problem at home then they can come back to the hospital. Because we cannot go to see how they are doing at home as Sir has mentioned about the financial challenges as it is not in the system at the moment . So whatever is possible to be done in the hospital, like for example we have a prosthetic unit here for the whole of Bhutan. We get patients with road traffic accidents or other causes and they stay here till they get the prosthesis and then they are taught with all the rehabilitative measures and finally they are sent home. So that’s how we do till now.

**So you mean that when such patients go home it is not that they do not need any more care, right? They still need care but they need not stay in the hospital. They can be managed at home but at the moment we are not able to reach them at home, is that what you are saying?**

Nurse in-charge, general ward:

Yes, we are not able to reach at home and as Sir (CMO) mentioned earlier if the training is given to everyone and taught how home care should be carried out, since the people in the BHUs knows their community very well, if they are trained in PC they can manage those patients at the community better. That’s what I wanted to say. (training the BHU staff)

**Thank you.**

**Now, as a physiotherapist, what is your experience so far? What type of patients have you managed and what were some of the challenges?**

Physiotherapist:

Since we are mostly in the rehabilitative side we do receive both acute cases as well as chronic ones. The common cases we get in Bhutan is the stroke patients. So we see cases like hemiplegia, quadriplegia, we do get brain injury and we also see patients with RTA (Road traffic accident). We see more of hemiplegia and quadriplegia cases. So when they come to us they are mostly emotionally disturbed after they are paralysed and they feel that they can never regain the functions of their limbs. They feel that they are going to be like that the whole of their life. With that much they are usually emotional disturbed. And one of the problems here is, as Sir (CMO) mentioned, we do not have a counsellor. You know we don’t have a clinical counsellor and so it is very hard to help patients understand. We do whatever we know and are skilled at. When such cases come, from our side we tell them about the rehabilitative services that are possible. ‘Even if you are not able to walk we do have devices, now that the country is developed, we now have wheelchairs with advanced features’ and through that we tell them how much we can improve their lives. Like if he is not able to move his leg at the moment but if his two upper limbs are movable we can train the brain and it can become so sharp that even if the legs doesn’t work he can survive using his hands that are not damaged. So we give such real examples from around the world where people are surviving using the wheelchairs. To every stroke case we rehabilitate the functional capacity and these makes them feel much better and today in Bhutan we have various organizations like Draktsho (a vocational training centre for special children and youths) and we give them examples like those institutes who can support them. And that is how much we do till they are in the hospital. After they get discharged we do not get to know how they are doing once they go back to their villages. That is one of the problems we have. There is no system where we can link to patients who are back in the community. We advise patients to follow up in their community health centres but we do not know how much they follow-up. And we never meet them again. Few we get to see them but not most of them. The other thing is, when a patient is admitted, say a cancer patient has come and some cancer patients are so much in pain they are bedridden. So when they are bedridden the pressure sores gives them so much of suffering in our context. So for such patient pain management in the form of oral medications or injections can be very useful. But sometimes we feel that the patient has pain, he/she is bedridden and there is no mobility and what we do is we try to give them some mobility. And we also educate the patient party on what all to do while at home. But we do not know how much they do at home that we do not know. Thank you.

**Thank you very much. So from the discussions on your experiences treating and managing patients with advanced illnesses we understood that the patients' needs vary, right? Now if you can elaborate more on what are some of the specific needs of these patients and their families? We are understanding that it is not just the physical pain, but they face whole lot of issues when diagnosed with an advanced illness?**

CMO:

Aah… I feel that they (patients with advanced illness) need lot of support you know. They need nursing care, nutritional support and all and I think these are some of the basic things that they need. And then one thing that we are forgetting is their spiritual needs that is more important. Most of our patients are very religious you know and spiritual needs in the home-based settings and in our facilities when they are admitted is important. We all know that spiritual needs are very important and that’s how we now have a Lama (Buddhist master) in the hospital. Basically one of our aims was to reach those patients who are at their end of life or are terminally ill. They can also have support, spiritual support, in terms of any rituals or prayers which are important I think. Even for patients who go back to the community I think those patients definitely need spiritual support. And the other important thing I think, just like for any other patients you know, like for any other diseases say like cancer survivors, they need to interact. They will be motivated. They will be basically prepare you know. For us, being a healthy normal person, we will not understand their suffering but if they interact with someone who has similar illness and problems and if they get to talk and share their stories they will definitely experience common interests and they will together explore various options of healing both pharmacological and non-pharmacological and basically they will look up for themselves.

**That’s very important I think, interacting with other patients. Any other needs that you have come across in these patients? Besides the physical needs, because physical needs are very obvious you know, patients comes in pain and we can identify it. And you have mentioned spiritual needs. Any other needs, as a nurse in the ward, have you seen or witnessed other needs? Because, as nurses, we see patients the most, right?**

Nurse in-charge, MDR-TB Ward:

Especially in MDR, more than anything what I have noticed so far is that the patients need their family support, more than anything else, especially in MDR. Like they need long duration of hospital admission and with family problem it further worsens their condition. Sometimes, like a husband having an affairs (with another woman) when the wife has TB and later there are cases that they even get divorced. They enter (go into) into depression. So for me, when it comes to MDR-TB, family support seems to be more important than anything else.

**I interviewed two MDR-TB patients this morning and one of the very important needs they mentioned for them was the need for family support, especially initially when they were here for the first time. They said they could not imagine having to live all by themselves. Having to take so many medicines.**

Nurse in-charge, MDR-TB Ward:

One thing is because of the stigma and discrimination that’s there when someone gets TB. Once it is TB even the husband doesn’t come close to the patient, he doesn’t do anything for her and that way we have had many divorce cases here and that causes even more psychological issues in our patients. So we have many such issues and our stories will never end if I have to share (laughs)

**So do you get to educate the family members? Do they come along with the patient when they first come in?**

CMO:

Initially during the admission of the patient whenever possible, if the time permits, and if there are accompanying attendants we definitely tell them (the family members) about all these issues and what we plan to do for the patient. But the challenge is that they (family members) also cannot stay here for their own safety and that’s why I really feel the need for a clinical counsellor who can counsel the patient.

**Right.**

**So just to summarise we have discussed so far on what motivated you to participate in this discussion, your experiences with so many patients with terminal illness or advanced illness and the challenges you faced, right? And training is one important area to be looked at, right? And the needs are not only physical needs but support needs, spiritual needs, right? Anything else that you want to add? Anything that we missed?**

Physiotherapist:

The financial need because we do face financial problems to reach the patients.

**You are right. In order to reach the patients at home you mentioned about the financial challenges. Basically a need for a systematic way to do, like how to reach patients in the community and how and who will follow-up, am I right?**

CMO:

I think we need a good policies for this.

Pharmacist:

Besides family support, because sometimes some patients do not have good supportive family members you know. They can be uncle or aunt and not their own mum or dad, so I feel for such patients we the health staff, especially the nurses, because they are directly in contact with the patients, if they can give a kind of a family support I feel that the patients will feel better in many ways. But sometimes if the health worker ourselves show our attitude, on top of not having a supportive family members the attitude of health workers can be more problematic to the patients.

CMO:

I think that’s very important. Our approach to patients, our attitude as health workers is very important. And for that I think we need people to be trained in PC and that will change the mindset of the people. This is one challenge we have

**I see. I think I will mention right here that from my two days of experience talking to the patients here in Gidakom Hospital, believe it or not, this is the only hospital where I got positive feedback from patients who said that they have never seen doctors and nurses anywhere else like they have here . They say you all are very good. Because you know I have been going around and I am a nurse myself, I am a nurse educator and I have often been very embarrassed. I haven't heard so much about the doctors but our nurses’ attitude is really talked about. Some of our nurses have issues even with a dying patients who is in the hospital only for pain killers. They deny his pain medications and had so many other issues. And today I am so happy, I am so relieved and told to myself ‘ok, finally, this is what I wanted to hear’ you know. They (patients) said there are no doctors or nurses like they have here in Gidakom. There is nothing to blame anyone and as Sir (CMO) rightly mentioned the importance of training for health workers that can change their mindset and their attitude. There has to be some motivating factors for the staff as well. So I am really happy to hear about how you all are treating patients despite having to care for patients with such serious and infectious nature. Because it is a kind of infectious disease where you all are at risk but you all are doing so great and that is very encouraging.**

**So that was the first part of the discussion where we were mainly looking at experiences, challenges and the needs. Aah… to move on PC also needs adequate medicines, especially pain medicines and other essential PC drugs. There is a list of them. Analgesics like opioids like morphine which I understand is very cheap and very effective for chronic moderate to severe pain. As a pharmacist, firstly do you have morphine here in your hospital?**

Pharmacist:

Yes, we do.

**Have you ever experienced where you ran out of stock and had to send patients to Thimphu or had to keep them waiting as you ordered or arranged from other hospitals?**

Pharmacist:

Umm.. I do really feel that we have morphine. I have been working here for one and half year and I think sometime we run short of this controlled drugs especially which are usually used for patients with pain. So as far as we in the pharmacy what we do is we try to get drugs from other hospitals. Like they do mobilization of the drug. So especially the store in-charge they have link with everyone so they just contact them and if they have enough stock in other hospitals so we try to get from them. The other issue is sometimes physicians will change the drug like if there is no morphine but if we have pethidine in the stock so we can even change to that drug until we get that other drugs from other hospitals. So even for other essential medicines they have come up with EBMSIS

**What is that?**

Pharmacist:

It is like through computer we need to update everything like within the country. All the drugs that are out of stock or if there is enough drugs, we need to update in that. So everyone will see it and whoever needs it or whoever has enough we try to mobilise. So this system has been developed since last year. So now every indent or every issue of the drug is all done through that system.

**And I think for Gidakom, because it is so close to Thimphu, it is much easier.**

Pharmacist:

Yes, it is much easier.

**How do you feel about the narcotic drug regulation in Bhutan? I mean do you feel that it has to be revised or do you see any stringent rules where the accessibility to the patient is limited or do you see that it is flexible and that patients have adequate access? What is your opinion on the regulation?**

Pharmacist:

Ok. My opinion on narcotic regulation I think aah…the rules are good enough. I mean the regulation is good enough because there is accessibility to the patients who need to come to a physician wherever it may be like the district hospital or BHUs and patients can avail through the doctors if they have any pain. Doctors can give them opioids and that’s how we use to dispense but then if it is more accessible then I am pretty much sure that patients will get addicted to it or there will be so much misuse of those drugs. So I feel that the regulation is good enough. I don’t think there is any need to change it.

**I see. I have also been hearing the same from other pharmacists. It is also important to ensure that we have a proper regulation you know, feasible regulations to support patients and I am understanding that in Bhutan we have regulation which ensures that morphine and other opioids are available enough for the patients but also that it is stringent to prevent abuse, which is good, right? Why I am asking this is aah… morphine is found to be utilised very less in our part of the world, in the developing countries you know. Although morphine is known to be very cheap and very effective for moderate to severe pain, literature says that more than 90% of the total morphine is utilised by the developed countries. Because PC is well established in developed countries and pain management is really adequate you know. The International Narcotic Control Board mentions that only around 5 -10% is going to the developing countries. And actually developing countries has much more patients because of the size of the population. We have much more patients who needs it but because we do not know about PC and adequate pain management morphine is utilised very little in our part of the world. So that’s why I am exploring on our narcotic regulations but every pharmacist that I met so far said that we have supportive regulations which is very encouraging. So the only thing is we need education and awareness on PC.**

Pharmacist:

Yes, and we also indent drugs according to the amount we use in our hospitals. For example, if we needed around ten ampoules of morphine injection this year then based on that we calculate for the next annual use and indent accordingly. So that’s why we may run short of it at times. And it depends on the physicians as well. They have different prescription patterns you know and that also can cause a bit of ups and downs in the stock.

**I see.**

**So here in Gidakom hospital, your main patients are MDR-TB patients, right?**

Pharmacist:

You are right.

**And I read in the literature and I have also heard from some of you in our personal discussion that MDR-TB drugs are very expensive. How do you manage the regular supply of drugs for MDR-TB? And today I am also understanding that it is not only MDR but there is also XDR-TB which requires even more stronger and more expensive drugs.**

Pharmacist:

You are right and we have two separate ways of indenting the usual hospital drugs and drugs for MDR-TB. For the usual drugs we have to do the quantification then we have to send it to MSPD (Medical supplies and procurement division) and only after they review they procure through the main budget. But the MDR-TB drugs, I just discussed about it with our store in-charge this morning and he said that every six months we need to report to the TB program office in the Ministry of Health on how much drugs we use. So based on that the program will procure the drugs. Once the stock arrives at their office then they inform the in-charges and it is collected accordingly. So they have different suppliers for TB drugs.

CMO:

It is also through the WHO incidence report, quarterly report, and based on that they indent from the global drug facility and especially for MDR and now the first line drugs are procured by the RGOB (Royal Government of Bhutan). The first line drugs doesn’t come from GF (Global Fund). It is only MDR drugs they supply through global fund and for XDR (Extremely Drug Resistant), because the cases are very less you know, the indent is based on case basis. If we have a XDR case, like we have pre-XDR at the moment and possibly they will go into full blown XDR because the second line drug testing facility is not there in Bhutan. That’s why we are able to diagnose only up to pre-XDR. So if we ever encounter XDR patient we then inform the program and the program will procure the drugs through GF based on a case basis. That’s why patient will have to wait till the drug arrives.

**I see. When it comes to XDR is there a cure for it? How is it?**

CMO:

Globally the cure rate is only 20% for XDR and I think that is also in the high resource countries. For countries like Bhutan, India or Bangladesh I don’t think cure rate can be even 20%. Because most of the XDR drugs are not really evidence based. There is not enough research done to prove that these drugs are effective on the bacilli. So they just give drugs on a kind of experimental basis you know. And the bacilli has gone way beyond the antibiotic although for almost half the century so many things have been happening on TB.

**Have you been seeing an increased cases of pre - XDR over the years or is it still very less?**

CMO:

So MDR is basically increasing you know probably because of the better diagnostic facilities and now we are getting more numbers of pre-XDR because for the last six months I think we already had six or seven and that’s because we now have second line testing facility, there is a machine in the RCDC (Royal Centre for Disease Control). Before we didn’t have that so before what we did was we sent all the samples to Bangkok supervised through lab WHO. So from there they did the testing but unfortunately we never got any reports so far till now for the last ten years or so.

**Why do you think is that?**

CMO:

There are some problems in the system and that’s why we never really know whether it is XDR or MDR. All XDR are MDR but not all MDR are XDR. From MDR few goes into XDR.

**That’s interesting**

CMO:

Around 90% are MDR. In Bhutan we are supposed to have at least eight XDR cases out of 100 MDR. And we already have one XDR.

**And the cure rate is very low you said, right?**

CMO:

You are right. And in TB it is interesting that there are other factors that are man-made but there are also other factors that are not man-made. It is the natural mutation. The bacteria is natural and that’s why among 100 MDR patients they will naturally mutate even though they are taking medicines and have good adherence the bacilli will mutate. It is a kind of tendency. So that’s how we have to be definite that there will be XDR. But we are not able to pick up the cases.

**Why I am emphasizing is because till now it is only in Gidakom where MDR/XDR patients are kept and so I am trying to understand how important PC is for Gidakom. That’s why I was interested to go into a bit of details. I am now understanding that the cure rate is very low and with those strong medications, I am sure they will have lots of side effects and also those psychological factors of being stigmatized and discriminated, as mentioned by brother (nurse in-charge, MDR-TB ward) and being away from family members I can see the need for PC.**

Nurse in-charge, MDR-TB Ward:

And when patients go from MDR to XDR it is even more challenging for us to help patients. Just recently we diagnosed two with XDR

CMO:

It is pre-XDR

Nurse in-charge, MDR-TB Ward:

Ya, it is pre-XDR and even till now we have not been able to convince the patients. It is hard.

CMO:

He (Nurse in-charge, MDR-TB Ward) is right and we will definitely have more XDR cases hereafter with better diagnostic facilities and because globally right now is only 20% cure rate. So in Bhutan it could be as low as 10%. So out of 100 patients who are on treatment 90 of them will die. So that is how important PC is. And as a physician we cannot do much and as I said even the medicines are not proven to be effective in a way. We are just giving these toxic medicines and these patients are going to have even more side effects which means they are going to be ill even with these medicines instead of curing. Now what we need is we need to counsel these patients because we do not know the outcome for these patients. Even the best physician cannot predict the outcome of XDR patient. This patient might respond or may not respond. That’s there. The chances of responding is very less, 20% in developed countries, which is less than 50%. So these patients might probably die and the chances of surviving is less. So right from the initiation of treatment we have to think that this patient, during the course of treatment we might lose him. So that’s where the importance of PC is. To give a better quality of life. We have to mentally prepare him that he may die or if not if he doesn’t die of XDR bacilli he might die of the medicines which are vert toxic. So these are the things which we are forgetting. Our TB management for drug resistance in the hospitals and for all the health workers really should be taught actually. Because unlike other diseases which are just a matter of time and it will be cured but in MDR, although our program says the cure rate is 93% but the ground reality is much lesser. Even MDR patients we are going to lose some of them. Because even if it is 90% cure rate 10% are going to die and in XDR at least 80% are going to die. So that’s why PC training is really needed for the health workers taking care of MDR patients.

And because we are never trained we never sensitized on this. No one is aware of PC. We do not know the responsibility of PC. Our approach to MDR patients till now is like to any other general patients and even if XDR patients comes our approach will remain the same. As if this patient will recover like any other patients we will continue to do our routine job which is not enough for MDR or XDR patients.

**So true. And the patients also have to be aware of the disease and its outcome, right? And equally the health workers including the treating physician and others taking care of these patients have to be aware and competent because there is a need to take care of these patients holistically.**

**Drungtsho, how do you feel about your role in taking care of MDR-TB patients? Till now are you involved in caring for MDR-TB patients?**

Drungtsho:

No, till now I am not involved.

**Having discussed about it here today do you feel that you can be a part of the team in taking care of patients with MDR-TB? Because we are now understanding that MDR patients will keep increasing and the issues will keep growing, right?**

Drungtsho:

Yes, as I have mentioned earlier because our traditional medicine is based on Buddha Dharma so we do have religious ways and means to help patients and that way I do feel that I have a role to be a part of the team. It is a kind of counselling that is required for the patients and as some of my colleagues mentioned about the support required for the patient mainly for their psychological wellbeing and in that if I participate I can see that I can benefit the patients. As I listen to the discussion from an external angle I am understanding that the survival rate for XDR patients is very low and that’s where PC can be very useful. Now my colleagues are the ones who are taking care of those patients and since you (researcher) are here my doubt is will there be some kind of training on PC?; and irrespective of the training will there be a guideline because we need to approach the patients, right? Educate them because one of my colleagues just mentioned that the patient could not be convinced and such challenges will be there. So from a Drungtsho’s perspective although we do not have the competency for the treatment part but I feel we can educate and counsel and talk to these patients and thus I feel that I do have a role to be a part of the caring team.

CMO:

And I have been fighting for a clinical counsellor for a long time and the first batch of clinical counsellors have graduated. The ministry has promised that they will give us one. And once he/she joins us we are planning to form a multidisciplinary team and we will include Drungtsho, pharmacist and many others and we can then discuss and for those patients who are not responding to XDR medications and are deteriorating then we can discuss whether we need to discontinue the medicines which are very toxic and very expensive as well. So we need to consider both these aspects and when there is no other choice of medicines we may just consider managing him conservatively towards improving his quality of life. So for that we are mentally prepared, I have already thought about that because if XDR cases comes we will need this for sure.

**That's a great idea**

CMO:

And we already have a Lam (a Buddhist master)

**Which is great, right? And Lam can also be involved in the PC team even at an initial stage of treatment. I think Gidakom hospital has a good potential for PC.**

**So we discussed about PC drugs and MDR-TB drugs. And then on the prognosis of MDR and pre-XDR TB and the need for PC, right? We discussed about PC team and a team approach of care. PC is patient and family focussed rather than disease focused. So as we discussed we came to know our potential. It is just that we need to be aware you know. I think we covered quite a lot.**

**Is there anything else you want to discuss?**

**Nothing...**

**So to summarise, this PhD project is aimed at developing a suitable PC model for Bhutan. As a summarizing question, do you have any comment, suggestion or an advice regarding the project?**

CMO:

Even though we have met today although it is for your PhD project but I think we all are interested in it and everyone has thought of something about it. PC is something which is important for everyone working in the hospital and as I have said earlier when we look at our health care system it has improved so much over the years and now we have reached a stage where this palliative care is very important. I think all of us here today understood how important it is. So today this is an opportunity for us and we will continue and we will do whatever in our own ways. You have provided us lot of information during the discussion because we do not know much about palliative care at the moment. All we do is the practical aspects and whatever little we learned from our practices. And I am thinking that we will start with the multidisciplinary team from January next year. Once the counsellor joins we already have a Lam and it may not be a perfect team but we will meet whenever required. And that is our plan and our interest as well. And we will also inform you how we come up. Usually we are all very committed. Our nurses are very caring and at times even if they have to go to see a patient although there are some implications but still we consider. We still send them. Our policies and guidelines do not support many things but these are things that are important and we try to do whatever is possible at our level. Your study will definitely help to frame policies and I think it is a very useful study. Personally I feel that this is very important and we are very happy that you chose to come here and do this discussion and recruit our patients. We really want to see something coming up after your studies.

**Thank you so much Sir. I agree and as I said earlier you have a caring and committed team here. Very encouraging.**

**Anything else from others?**

Drungtsho:

If I may say, even the traditional medicine, like Sir mentioned about XDR being not an evidence based, many feel that traditional medicine is not evidence based but traditional medicine has been practiced for so long and at the moment we have MSP which is Menjong Sorig Pharmaceutical, it is a kind of company or a factory where we produce traditional medicine. So this company has been supported by international funding projects like DSP and later EU. So even if these international bodies supports the company they could not really do a research because the ingredients in our traditional medicine are so many. If it was a single ingredient like in the allopathic medicine it can easily be studied you know. So likewise when it comes to PC for terminal case if the patient doesn’t have physical pain and who do not need any medicine there can be some placebo may be and in traditional medicine we call it symptomatic treatment (*tadha thipai*) which we can give to the patients to relieve certain symptoms. The other thing is as I mentioned earlier about the counselling and as you are doing this project madam, we also have what is called from a traditional medicine perspective *Ayush*, aah… Ayurvedic, homeopathy and so on. And in Australia when I asked people they said there is nothing like this, may be homeopathy but for us in Bhutan the Bhutanese traditional medicine this can go as a holistic approach of care. So if we can include traditional medicine as well because we can give them education, counselling and even if our traditional medicine do not benefit it is not known to cause so much harm . So I feel this is a strong point.

**I agree. Traditional medicine do have a potential for palliative care. Thank you Drungtsho.**

As an in-charge of the MDR-TB unit, because I am really amazed at how you are managing every day, caring for such patients. What do you say about this project? What is your advice to me?

Nurse in-charge, MDR-TB Ward:

As we wind up now, maybe I am not the right person to recommend or give feedback (laughs) but like with an increasing number of terminal illness like cancer, ALD and other diseases I think it is high time that we prioritise in PC. Like yesterday when I first saw you coming to our doctor I thought you were a patient coming for review (laughs) but later when I knew that you are doing PhD in PC it actually aroused some kind of curiosity in me and like I am really curious to see what madam has to come up with in a few years’ time, develop a suitable PC model for our country and maybe it will benefit us one day. That’s all.

**Thank you**

Pharmacist:

Aah… I personally feel that this project is a very good initiative and then as a pharmacist I think we can play a big role in PC. At the moment we are more of like ‘drug oriented’ especially we are with drugs only but till now pharmacist were never like involved directly with the patients. Now every time during some workshops or training the senior pharmacists they usually focus on patient orientation as we were mostly drug oriented you know. And PC project is usually with patients so I feel that it is very important that as a pharmacist also we can get involved when there is some treatment part of the management to be done and we can give suggestions on medications. PC patients are usually terminally ill and they don’t want to take too much medications so that’s why as a pharmacist also I think we can minimise those so many drugs and then and minimise this ‘polypharmacy’ which is happening. And Bhutan, because of the economic status also it is quite difficult compared to the advanced countries. In advanced countries some of the drugs which the patients cannot take orally they can also be given by transdermal and other means but in Bhutan we provide free services we know that, right, and so it is really difficult to directly change this routes of administration. So I hope that with PC model which you will hopefully come forward with and I hope that we (pharmacists) can play a big role in it. Thank you

**Thank you**

Nurse in-charge, general Ward:

Well, actually it is like what my colleague (Nurse in-charge, MDR-TB Ward) here has said. With hope and wish that your study turns out to reality and that we have SOPs (standard of procedure) in place so that we can benefit the patients. We say patients but one day we may be the patient. So it can benefit all of us in general (laughs).

**Thank you**

Physiotherapist:

All I want to say is that I have expressed everything about physiotherapist’s role and I don’t have anything more to add (laughs). This PhD project on PC for which you are almost done with data collection, one thing is that we need to have policy in place. And if this is not got into the heads of the policymakers then it would be difficult. So madam might be working towards it but I would like to emphasize that you please make sure that this really gets into the heads of the policymakers (laughs) because it is high time now as we know that NCDs are increasing and due to better healthcare our lifespan is increasing. So PC is very important to be in place in the health settings. We all know that we will die once, that everybody know, but how we will die nobody knows. So at that moment, right now we are young, we do not have disease, so we seem to be healthy, but we cannot predict the future. So right from now if we have a PC model and if we support it, have policy in place, we may be the recipient of PC who knows. If it is an accident and we get killed on the spot then it is ok but if we are to get a life-long chronic illness then PC is what we really need. Even if we are not worried of today we have to think of the future, we have to think about ourselves I would say. So it is very good that madam is working towards this model. May it get integrated into the policy and come out as reality and we have our 100% support. Whatever role we may get to play but we will play 100% role and we will continue to support for PC I would say.

**Thank you so much. Looking at your motivation I am encouraged to work even harder.** **I will make sure policy makers are involved.**

**Is there anything more you want to say before we close? Anything that you feel is important to discuss?**

**Nothing right? So thank you very much. At this time all I can say is Thank You so much. I mean it from the bottom of my heart. Thank you for giving me your time and for your very invaluable information which will be very useful for the project.**

**Thank you Sir, thank you everyone.**
