# Supplementary material for: Healthcare professionals’ views on how palliative care should be delivered in Bhutan: A qualitative study
Source: PLOS Glob Public Health. 2022 Dec 12;2(12):e0000775. doi: 10.1371/journal.pgph.0000775 (PMC10021767; doi:10.1371/journal.pgph.0000775)
Supplement: S12 Data — (DOCX) [file pgph.0000775.s013.docx]

**Field Note of FGD with Health workers in Gidakom Hospital**

Date: 24.7.2019

Venue: Office of the Chief Medical Officer, Gidakom Hospital

| Dr Chencho Dorji Participant 1 | 111 |
| --- | --- |
| Tshering Yangki Participant 2 | 112 |
| Loday Phuntsho Participant 3 | 113 |
| Neten Gyembo Participant 4 | 114 |
| Sonam Tenzin Participant 5 | 115 |
| Drungtsho Participant 6 |  |

This FGD was conducted in the office of the Chief medical officer (CMO) of Gidakom hospital. The participants included the CMO, two nurses, one each from MDR-TB ward and general ward, a physiotherapist, a pharmacist and Drungtsho, the traditional physician. Although most of the participants did not know anything about palliative care they all became interested to participate in the study after reading the PIF and they were all curious to learn more about it. Each participant felt how they can be a part of PC team and the crucial role they can play to improve the quality of life of patients with advanced illness and those who did not have the prospect of cure. They expressed how important PC has become today.

No one on the group was dominating or spoke too less. They were all eager and curious to express in the discussion. It was a good decision to have included Gidakom hospital as one of the study sites as this is the only hospital in Bhutan to take care of patients with MDR-TB and now there are even patients who go into XDR-TB and who really needs PC as the treatment and cure prospects are very limited I was told during the interview.

The support and interest the participants have expressed for PC was very encouraging and motivating and Gidakom is seen to have a very good potential to integrate PC. It was a very educative and fruitful discussion.

Thank you

(Last time I went to see Lyonchen (Prime Minister) because I thought I will appraise what I am studying because it is a new field you know and also at the moment in the national referral hospital there is something coming up in PC. They have formed a home-based team and there are just the doctors and nurses in the team and their main focus is on cancer patients. They go to see patients at home mainly focusing on pain management. They are doing great despite so many unimaginable challenges you know. So that’s why I went to see Lyonchen. He asked me what my project is on. So I said it is on developing a suitable PC model for Bhutan and I was saying that I thought it was a best one because we don’t have a concept of PC right now. May be I was wrong to say ‘no concept’ but I was trying to say that we do not have PC service in Bhutan. And Lyonchen said ‘ohh ..its not that we do not have the concept of PC. We do have the concept of it because when we have a very ill patient in the ward we at least have a feeling of concern and compassion towards the patient, don’t we have? So we do have the concept of PC” His Excellency said. And I was saying ‘but Lyonchen PC is not just that. It is a multi-disciplinary approach of care and it has to be a specialty’ because it is a specialty and it requires a team. And he said ‘you must be right because I have just heard about PC and I haven’t had any training. So I don’t really know’ he said. So many thinks that it is just a small component)
