# Supplementary material for: Healthcare professionals’ views on how palliative care should be delivered in Bhutan: A qualitative study
Source: PLOS Glob Public Health. 2022 Dec 12;2(12):e0000775. doi: 10.1371/journal.pgph.0000775 (PMC10021767; doi:10.1371/journal.pgph.0000775)
Supplement: S13 Data — (DOCX) [file pgph.0000775.s014.docx]

**Field note on FGD with HCPs at JDWNRH**

Date: 14/8/2019

This FG was planned since the beginning of fieldwork some times in May 2019. Every doctor/specialist, nurse, pharmacist and physiotherapist I approached for participation was interested to participate in the FG but their availability was an issue. Most of them were so busy they could not give a specific time when they would be available. It was very difficult for me to confirm a date because when one was available the other would be away or engaged. And I wanted to involve as many right people as possible since this is the national referral hospital, the apex tertiary care facility, in the country where any kind of patients with advanced illnesses are treated and managed. Following their participation in the survey, I could finally confirm with the ENT surgeon who is known to be very concerned about the patients’ holistic care; then the only nephrologist in the country where CKD is increasing rapidly; the orthopaedic surgeon; the pharmacist who was involved in introducing PC in Bhutan; a physiotherapist and two nurses from medical ward and adult ICU.

The date for FG was finally decided for today the 14^th^ of August 2019 at 2 PM, the last day for fieldwork as I am flying back to Perth day after tomorrow on 16^th^ August. A simple lunch was organised for the participants at the Faculty’s canteen (ground floor) and the venue for discussion was decided to be the Conference Hall, Faculty of Nursing and Public Health (second floor). However, at around 1 PM there was suddenly an unexpected heavy downpour making it difficult even to come from the hospital to the faculty, which normally is a 5-minute walking distance. I was scared my participants may not turn up. Nevertheless, everyone managed to make it except the orthopaedic surgeon who called me to say that he neither has an umbrella nor a car and that he cannot come. I even offered to pick him up but the rain was so heavy to even get out of the room and for which he denied. At around 2.30 PM rest of the participants turned up most of them all fully drenched. We all had lunch and finally by 3.15 we could start the discussion.

Once the discussion started everyone forgot that they were wet or cold. The discussion picked up so well and everyone participated fully no one was in haste to rush home. Everyone acknowledged how important PC is for Bhutan and emphasized how it should be in our context. Having the ENT specialist in the group was phenomenal because of his vast experience on the needs of patients and his knowledge and passion for PC. Others were equally interested.

I am so happy that this FG, which was the most important one, has happened at the very end of the fieldwork and nothing - no rain, no storm, no thunder could stop it! More than anything else the participants were remarkable. There was a very satisfying and thorough discussion which went for 1 hour 29 minutes 42 seconds.

Thank you
