# Supplementary material for: Healthcare professionals’ views on how palliative care should be delivered in Bhutan: A qualitative study
Source: PLOS Glob Public Health. 2022 Dec 12;2(12):e0000775. doi: 10.1371/journal.pgph.0000775 (PMC10021767; doi:10.1371/journal.pgph.0000775)
Supplement: S14 Data — (DOCX) [file pgph.0000775.s015.docx]

**Transcript of FGD with HCPs at JDWNRH on 14.8.2019**

| Participant 1 | ENT Surgeon |
| --- | --- |
| Participant 2 | Nephrologist |
| Participant 3 | Nurse, Medical Ward |
| Participant 4 | Physiotherapist |
| Participant 5 | Nurse, ICU |
| Participant 6 | Pharmacist |

A very Good afternoon to everyone. Thank you very much for making this possible. Despite all these rains and storms and thunders and everything..*laughs*… *Everyone laughs..*

So today to begin with, can we first discuss about what motivated you all, six of you here, to participate in this focus group discussion. I know I have somehow been behind you for these many days …*everybody laughs*…but having read the PIF and having participated in the survey I am sure you did derive some kind of motivation to decide to participate in this discussion. So maybe we will start with that.

ENT surgeon

So I think the first motivation of course is you yourself aah…coming up with the topic and then trying to explain and I think…when you, initially I was not aware that you are doing this. I somehow heard but when you really came to me with this thing then only it was confirmed that you are actually doing a PhD in PC. And what I felt was this is a very …we all know that there is a urgent need for PC service in our country because so far we don’t have…we do have but in a very small scale. But as you rightly pointed out we really need to integrate PC into our system. Right now it is happening in a very ..you know …not in a systematic or you know in a proper you know large scale manner. So when you came up with this topic I thought it is very relevant for our country and also somebody actually doing a PhD in PC. So that is something which I found it very nice and then I thought maybe if there is a discussion where we could actually …since we are dealing you know.. with end-of-life patients on a daily basis…maybe something we can discuss from our experience in dealing with such patients..

Thank you Sir

Nephrologist

So more or less it is same and in the similar line. Since you are studying this important subject and we know there are increasing cancer cases, CKD (Chronic Kidney disease) patients and so many other chronic cases. Other cases like the gynaecological cancers seems to be ever increasing in the country. Your study in this field I feel is very timely and with the findings of your study it will help us to understand how to handle with such patients.

Thank you Sir

Nurse, Medical Ward

Aah.. same thing with me.. palliative care you know like it is timely. With the advancement in medical technology more cancers are getting diagnosed. Five years ago the diagnoses of cancer was comparatively lesser. Now even as a nurse you know, dealing and working in medical ward, it is like among every ten or fifteen patients one or two are cancer cases. And after diagnoses we almost think that for cancer there is no treatment. It seems like that. So I think, palliative care has to come to action now because it is timely and as sir (participant 1) mentioned, it is urgent. As madam (researcher) was sharing. We do face challenges in spiritually and culturally appropriate care. And Bhutan has many different culture and when it comes to end-of-life care, if we are not aware, we might go against their beliefs and culture you know. But if we know about palliative care then people need advocacy and I think people should know that palliative care exists and then it does bring lot of difference into someone’s last days in his/her dying bed.

In their quality of living and dying, right?

Yes

And that’s why you got motivated to participate in this discussion?

Yes

Thank you.

Now as a physiotherapist, what motivated you to be part of the team here sir?

Physiotherapist

Aah… the main motivation is I thought that it is quite a new idea which is being integrated in Bhutan. But in other places it is quite advanced. Since I am currently working in the ICU and surgical ward, I see most of the patients diagnosed with cancer and looking at the faces of these patients and their caregivers it is quite depressing because we know that the patient is almost at the terminal stage. So what I want to see in the coming years is the implementation of this PC. I want to see that change. I want to see the change with what is happening currently and maybe ten years down the line what could be the response from the patients and the caregivers.

So the change from that depressive look of the patients and families to a more cheerful faces you mean?

Ya, more acceptance and better quality of life

Thank you

As a senior nurse in this hospital, what made you feel like participating in this discussion?

Senior ICU nurse

As I am working in the ICU, adult ICU, we receive almost always dying patients, not only due to cancer but other cases as well who are dying. So PC is needed not only for the patients but it should also be for the patient attendants (family members) as well. They are suffering equally as a patient attendant, physically, mentally. So we talk with patient attendants as almost all our patients are unconscious. I want to see the acceptance of the situation among the family members. That is why I am motivated to participate in this discussion.

Thank you. It is not only the patient who is suffering there but the families, the relatives and the attendants who are there as well, right?

Yes

Thank you sister.

Now, a man behind PC. Everybody is in the frontline you know, the doctors, the nurse, the physiotherapist but an important part of the team is pharmacist. So what motivated you to be here today Sir?

Pharmacist

Thank you. Aah… I was a part of the initial part of the setting up of PC service at JDWNRH (national referral hospital) and knowing the state of PC service that we have in our country aah… actually I needed no second thought. As soon as you came to me and asked me if I could be a part of this focus group I needed no second thought. So that’s why I am here.

That’s very encouraging. Thank you.

Now the next question is, what are some of your experiences in managing or treating patients who are diagnosed with an advanced illness? Experiences and the challenges you know?

ENT Surgeon

Since I deal mainly with head and neck cancer patients, so one of the key things which happens to patients with head and neck cancer is the kind of treatment modality we offer for the patient. It is quite aah… sometimes quite brutal. Be it surgery it is sometime you know, because most of the time we end up doing a major surgery where we resect .. do a lot of resections, so patients they sometimes end up with disfigurement, sometimes they end up with lot of functional problems. For example difficulty in swallowing, sometimes if we do a laryngectomy then patient won’t be able to speak. So all kind of functional issues first thing.

Second thing then even other modalities of treatment like radiation and chemo. Although there are instances where radiation and chemotherapy are curative but it takes a lot of toll on the patient. So apart from giving a cure it also have a lot of side effects and especially side effects related to radiation last for many sometimes even for two to three years. So those things are quite a challenge because first of all it is a diagnosis of cancer, second thing even if you give a treatment, that treatment itself has a huge toll on the patient. So it means that we really need to take care of the patient in a wholesome way. Not just you know looking at the curative point of view but also looking after the patient in all other different perspectives. So far one of the most challenging thing is especially when the patient comes with a recurrence. That is a huge challenge that I face because ok if somebody gets cured that’s fine but we have limited options. We do surgery and if it recurs then another best option is again surgery but if the patient is inoperable by that time other options are like quite they are not curative. So they will lessen the disease but they won’t cure the disease. So then what happens is, for example somebody with oral cancer comes back with recurrence and if the surgical option is exhausted then next option is giving a palliative chemo or palliative radiotherapy which doesn’t have a cure but again has the toll on the patient. If he comes with a big ulcer in the neck then until the last breathe of the patient he suffers. So we try to lessen the patient’s suffering, we try alleviate the pain, we try to lessen the discomfort but whatever we do sometimes it is quite difficult to alleviate that completely. And despite all the care and support we give patient undergo through a lot of suffering. So that is a huge challenge but at the same time something which we cannot do to the max is the main challenge that I face.

What are some of the needs in these patients? Like you mentioned about the physical pain and discomfort. Are there other needs you see in such patients when you deal with them?

ENT surgeon

I feel that, personally I feel when we talk about PC we should not look at it as a care given to a terminally ill patient. I think that is a wrong concept where most of the people has. Palliative care has to be integrated into the patient’s care right from the beginning. Because we have to prepare the patient in a stage wise in a graded manner. If we bring PC right at the end of the thing then the name itself become misnomer. Then palliative care becomes really misnomer which means no hope, no nothing you know. So PC has to be integrated even if we give a curative treatment in has to be integrated. So even if the patient is not helped by the curative treatment yet he is already prepared and that is something more comforting. Other comforting options are available. So that has to be made understood as a team but unfortunately one of the biggest challenges we have right now is we do not have a multidisciplinary team. Any cancer case it has to be managed in a multidisciplinary team approach which we don’t have. So right now in head and neck whatever decision that I take is final but that is not ideal if we work in a centre which has all the facilities then we have tumour board and there should be a surgeon, there should be a radiation oncologist, medical oncologist, speech therapist, pathologist, everyone should be there. The whole team should be there especially nurses who can give proper care to the patient which we don’t have right now. So even if we have some experience with the PC because of our exposure outside, even if we try to implement here because of the kind of support that we have which I mean is we don’t have a system in place. So it is quite difficult to even if we have the intention, even if we have some experience to give PC but because we don’t have a system in place it is a big challenge.

Thank you Sir.

Sir you are the only nephrologist in the country, and kidney failure or CKD is so much a growing problem and a huge concern, right Sir? Sir what is your experience and some of the challenges in taking care of the CKD patients?

Nephrologist

You have rightly said that CKD diagnoses are increasing and it is not only here in the national referral hospital but regional referral hospitals also there are significant number of kidney patients requiring dialysis. And those patients requiring dialysis is just tip of the iceberg. Aah…the problem we have here is lack of facilities especially the dialysis machines. We have around more than 150 patients currently undergoing dialysis in the national referral hospital and another 100 in two regional referral hospitals and we have only ten machines here, which means our patients here, while ideally each patient should be getting three times a week we can give hardly once in a week. So the problem arises mainly because of inadequate dialysis machines. The complications arising out of inadequate dialysis, fluid overload, some metabolic volume disorder and other imbalances. These ae some of the problems that we have. Despite me and other in the team wanting to do our best we are being limited by this scarce facilities that we have here. even if we want to admit the patient but we don’t get bed. For example, patient has fluid overload and everyone can see that but yet, because others are already scheduled and others are equally important..*smiles*…we cannot even if I may request the dialysis nurses there it is very difficult for them to make space unless it becomes a life threatening. So this is one area. Then other thing is the availability of beds in the wards. So even if you.. I mean those with moderately severe cases needs to get admitted in the ward but there are not enough beds. So we have to manage them as OPD patients. So these are some of the difficulties and challenges that we have. And even for ones who are admitted because everyone is overworked because of less number of staff, doctors try to finish the rounds fast so that they can attend other patients. So these are the challenges related to kidney patients in this hospital.

Sir, besides the limited number of dialysis machines, that you mentioned, what are some other factors that affects the QOL of a kidney patientshere at JDWNRH?

Nephrologist

Well, I mean the main reason that affects the quality of life is dialysis. Then the others like the treatment modality for ESRD (End stage renal disease) is renal transplant. But there are issues and problems associated with that like difficulty in getting the donor, and so on. So these are some and even if you get a timely dialysis, what we should understand is dialysis is just a partial replacement for the normal kidney function. So despite having that patient could still have problems not as much as or not to the extent as patient not getting adequate dialysis. So challenge in other area is getting enough donors, kidney donors.

I have been to the regional referral hospitals, met patients with chronic kidney diseases and interviewed them. And because they have to live nearby hospitals as they need to come for dialysis often, at least 2-3 times a week, and also they cannot be kept in the hospitals admitted only to receive dialysis. So these patients faced several logistic issues. I learned about the new dialysis unit that is coming up, it looks so giant, infrastructure wise, and encouraging for the society and for those kidney patients. How can this centre make a difference to the QOL of patients?

Nephrologist

At the moment all the patients requiring dialysis and other patients like cancer patients and even others are all kept in the same guest house and it is overcrowded there. So I think once the new Dialysis Centre becomes functional I think there will be enough rooms for patients and their families. And that would, to some extent, improve the QOL. Other thing is we will have extra number of machines (dialysis machines) there while keeping some of the machines here (at the current location) for the indoor patients. So with those machines, I think it will be around 20 -25 machines, with the current number of patients we will be able to give adequate dose of dialysis, at least three times a week. And that will also improve the QOL.

Thank you Sir.

Can we now discuss about the needs of the patients with advanced illness like cancer, or any other non-cancer conditions and at end of life?

Nurse, Medical Ward

The way we look at it depends. Some people accept it, like for some they are diagnosed with cancer and were on supportive care for a long time. So for such patients, we do not have to go and give counselling. They accept it. They would say ‘it has been long with the illness and it is ok’.

So is it the patient or the family who accepts?

Nurse, Medical Ward

The family

How about the patients?

Patients at the end-of-life, they would already be unconscious, so we and the treating physicians treat them by giving analgesics like morphine, codeine and sometimes IV opioids also.

Ok. That’s interesting.

Sir (physiotherapist) what do you see in your patients who are there for months or years sometimes coming to physiotherapy to be better and with lots of hope maybe.

Physiotherapist

Thank you once again madam. Since I see patients who are mostly bed-ridden, so in that case, from a medical point of view, our doctors, our surgeons and our nurses are doing a great job. But what I do is I try to look at from the musculo- skeletal point of view. So most of the patients, like cancer patients, they are very weak. Their muscles are all atrophied and what I try to do is at least it is better for them to be in an upright position than in the sitting or lying position. So I encourage them from a lying position to either a sitting or if possible I make them ambulate. But it is very difficult since they are physiologically very much compromised it is very difficult to encourage them. So as far as possible, I try to at least maintain their muscle tones and if they have any secretions, since they have been bed ridden for long time, they will have compromised cardiovascular system, so as far as my competency, I try to do some simple chest manipulation technique just to remove the secretions. That’s all what I try based on my competency.

And do you see that it improves their QOL?

Yes, to some extent.

Sister (ICU nurse), do you have anything different to say when it come to the needs of the patients? You did mention about the family members who are an important part of PC. What are their needs as well?

Communication is one of the needs. Nurses should know how to communicate with the patients and their attendants and to support them. Let them know what is happening and what is being done to their patients.

Thank you so much.

Now, what is your understanding on palliative care? I know some you are aware of it. How about others?

(*Everybody indicates they know PC*)

So everyone knows what is PC, right?

Nods...

Great. So knowing what is PC and having been taking care of patients with advanced illness and having discussed the challenges, how do you see the future of PC in Bhutan? Or how can we make it happen so that our patients and families have better QOL than what they have now?

ENT Surgeon:

Actually what I feel, as I mentioned earlier, it (PC) has to be a part of the system because one day there may be a very passionate and enthusiastic person who might take up PC but if there is no systemic support then that person won’t be able to move forward. We have already seen that PC is a multidisciplinary approach. One single person cannot handle it. So everybody has to work as a team and to work as a team a system should be in place. It cannot succeed based on the motivation or compassion of a single person. So I think first thing we need to do is to sensitise our policy makers on PC, on the issue of PC. Because right now the concept is quite new in our country. Forget about PC even some of the common cancers, if we go and talk to an educated person, so called educated person, they have very minimal knowledge. Lot of awareness need to be raised. So PC being relatively new we have to sensitise our people, we have to sensitise our own health workers. I think that is very important because most of the health workers I don’t think they will have an idea about what PC is all about. Right now what I have seen is the moment they hear about PC it is like patient is going to die and they want to dump somebody you know. So that should not be the case. In order to change that, first of all we should raise awareness, we should target health workers, general public and policy makers and we should integrate PC as a part of the system. And then second thing, what I felt was, especially in a busy hospital like JDWNRH, where apart from giving PC services we also need to concentrate on other cases where turnover is very high, you know. So for a centre like JDWNRH we really need a hospice, where we can properly take care of those patients needing, terminally ill patients who need proper care. Not in a general ward setting where even our staff will lose focus, you know. Even the physician will lose focus and in a way patient will be neglected. So for me I feel hospice is quite a requirement in a hospital like JDWNRH. Because if we really need to focus our attention on patients who actually need PC, that will benefit them. So I feel that is important. And the third thing is the benefit of PC should spill over all the districts. Even ultimately, because your topic rightly pointed out, our PC should be customized to our Bhutanese setting, you know. We cannot take out the PC idea from a developed country like Singapore and US and apply here because our culture is different, people’s expectations are different, and our religion is different, lot of cultural influences. So we need to play around and then customize the principles of PC to our own setting. So ultimately the goal should be even at the level of BHU people should have access to PC. They may not have access to specialised PC but they should have access to basic PC. So ultimately for a dying patient the goal of PC is to relieve suffering and then to allow that person to have a dignified, painless death. So that should be available even at the level of BHU. Patients should not come to Thimphu to die. PC should be at their doorstep. I feel those three things are very important as far as PC is concerned.

If the Ministry can create a program just to prevent suicide why can’t they, you know, because in Bhutan if we look at, it is not published yet but recently we went through the cancer registry data from 2014 to 2018. We have just analysed, we haven’t published the report yet, but we have found that more than 3000+ patients. And out of that, that is just the number of patients, and if there is a cancer patient in the family see the impact of cancer on the family.

Exactly

It is a huge impact. But unfortunately, so far, our ministry hasn’t realised that. So I think from the Ministry also they should take a very, because cancer will definitely become like an epidemic. Right now we are still focusing on infection but we are being late to realise that cancer will hit us in a big way. Our data is showing that. We don’t have to go anywhere. Our data is showing 3000+ patients with cancer for last 5 years, see the impact on individual families. And right now in the ministry they don’t have a single program to look after cancer. Cancer is taken under the umbrella of NCD. They have other things to look after. They need to look after diabetes, hypertension, lot of other NCDs and cancer is losing focus. It is high time Ministry of Health should invest in cancer because ultimately, I mean time will come when cancer will become epidemic when it will affect each one of us. WHO has already predicted that. Once you cross the age of 50, you know, one in 4 person has a risk of getting one of the cancers. So you can see the impact. And if you are not prepared and you are still lost with HIV and TB and all these then by the time we respond I think it will be too late. So I think now even the ministry it is high time that they have it in the plan to create a separate program for cancer. That dedicated program manned by people who know, not by some general graduates appointed as a program officer. That will not work because somebody who handles the program should be competent, should have the idea, and should have the knowledge to run the program perfectly, you know. So that should come out and if we do not have a cancer control program dedicated in the ministry of health looking after cancer issues then it will be very difficult for PC to come as a system. So first and foremost we need to have a dedicated program in the Ministry of Health as a cancer control program and then PC should come as a major player in there. So then it will be embedded in our system. Then the benefit can trickle down even at the BHU.

Thank you Sir. That was a package in itself.

Now let us talk about drugs. PC is also to do so much with physical pain and other physical symptom management. Sir (pharmacist), what is the state of analgesics, particularly opioids ,because that has so much of role to play in managing moderate to severe chronic pain like cancer pain that cannot be managed by other firstline analgesics. So what is their availability and accessibility to the patients today?

Pharmacist

I think we have come a long way. Since the PC service was started by then we had good number of opioid analgesics available. We had oral morphine, codeine and we also had opioid injections like morphine and tramadol. So with the introduction of PC service we had a fair bit of discussion on what new opioid analgesics to be included in our formulary. And I am sorry we already had fentanyl patch and injection by then but that was basically for the use in ICU but fentanyl patch was for PC. But with the introduction of PC service, with the formalization of PC service we had a team working on it and I was actually given the feedback to include, to expand the basket of opioid analgesics and we recently included the sustained release morphine. So it should be available by this supply cycle. So we will have morphine SR tablets soon which will actually be convenient for PC patients because this will reduce the frequency of analgesics.

From the literature I understood that majority of morphine that is produced in the world, I think about 90 - 95%, if I am not mistaken, goes to developed countries, way beyond what is actually required there to manage pain. And only about 5 - 10% is utilised by developing countries where it is actually needed the most. It is a sad reality because being poor compromises so many things any way, right? I think being poor, being under-developed or being a developing country means being uneducated, being unaware, and being ignorant to a large extent. When we are uneducated and unaware, we don’t even know the importance of what is needed, like these drugs. I understood that morphine is one of the cheapest yet one of the most effective drug for pain management. Today, after having initiated PC, with whatever little is happening, what is the state of morphine availability in JDWNRH?

Pharmacist

That is certainly a challenge. Maintaining adequate stock of not only morphine but other opioid analgesics is certainly a challenge. I think it is mainly to do with the control that is put into the availability of opioid analgesics because we just cannot get it from anywhere in the market. I think it involves a yearlong plan. So it involves giving the quota. I think each country is allotted a quota of opioid analgesics, how much a country is allowed to import. So I think that is being strictly controlled by the narcotic control board. And our narcotic control board is further controlled by the INCB, the international narcotic control board. It is the board that controls the supply of opioid analgesics. So that process is really hectic. You can’t just get, if you run out of morphine tablet you just can’t get it. There is just no way to get the additional supply. So you have to go through the same process. That’s why we land up controlling the stock of what is available because if we just give it as it is demanded by the patients then maybe we will have no stock to spare for other patients. So there certainly is a challenge actually when it comes to maintaining adequate stock.

As a pharmacist, who is also now involved with setting up of PC in JDWNRH, do you see a need to review or revise the narcotic drug regulation of Bhutan?

Pharmacist

If possible, yes, from the accessibility point of view but there are other overriding factors and the narcotic control board must have considered those factors. So, like I said, it is a challenge to strike that fine balance but I think with our experiences growing I think it is only a matter of time. But we also need to really work it out comprehensively considering the immediate increasing number of patients who will require PC. In the near future we will need to do a good homework on it.

ENT Surgeon

Can I add something to what T has said?

Yes please Sir

ENT Surgeon

T (the pharmacist) is very right. The main reason why, now the usage has increased. So there are lot of prescribers who started prescribing morphine especially after the PC course. Initially people were quite scared of prescribing morphine but now they are quite comfortable after the course that we have attended. And as far as the regulation is concerned the availability of morphine won’t change just by changing the regulations in Bhutan because we do not produce morphine. Often we should import morphine. Just to give you an example, if you want to bring morphine from India the regulation is so strict that you cannot bring by air. If you bring by air then it is a huge hassle. So you have to bring by road. That is the only way even if you have to bring it on an emergency basis. And when you bring from road by the time the drug enters Bhutan from the production factory, the simplest example, to Bhutan they have to cross five check points. At each point they have to pay Rs. 100,000 (Indian currency which is equivalent to Bhutanese currency) as bribe. Because the regulation is so strict, if you want to have an easy access, even if you want to purchase on an emergency basis and if you want to bypass those gates then at each gate you will have to pay one lakh Rupees bribe. So that is the huge bottleneck. Why does that happen? Because the regulation is so strong even in other countries. So just because we change our regulation in our country who depend on the import from other country that cannot do anything. By relaxing the morphine law in the country will do nothing because we are dependent on others. So until and unless we as a region, as a region you know, try to do something about this regulation. It is one thing to change the regulation and one thing to make sure that morphine is used and not abused.

Exactly

So both system should be in place. Because if the narcotic regulation is loose then there is a possibility that it might lead to more availability and more abuse. So we should strike a balance between relaxing the regulation as well as making sure that the increased availability doesn’t lead to abuse. So both of these should have a fine balance. And the changed regulation should happen in the regional level. If we were a morphine producing country then it was fine but because we are based on imported supply we cannot do anything.

Thank you Sir. That was very informative and educative for me.

I have also understood that the International Narcotic Board is flexible when it comes to accessibility to narcotics for palliative care and pain management, they are not as stringent in issuing narcotics like morphine. But because our region obviously do not really understand well about PC and the importance of pain management, I feel we are not able to procure adequate supplies of opioids or narcotics. If we educate our policy makers and give them evidences on the importance of this cheap, yet very effective morphine or other analgesics, will we be able to help patients better?

Because at the end of the day that is our concern, right? Because if the physical pain is not managed adequately then other problems like psychological pain, emotional and spiritual pain is least likely to be managed as he/she will be so distressed with the physical pain. So when it comes to managing physical pain with drugs, how can we improve the availability and accessibility to opiods, especially morphine?

Pharmacist

I think, like you said, continuous availability of opioid analgesics I think becomes a cornerstone of pain management in patients requiring PC. So in that sense, to ensure continuous availability I think there has to be flexibility from the authorities involved. For that I think the policy makers have to be sensitized on the requirements of the patients. So like Dr P (ENT Surgeon) mentioned earlier we can have a program in the MoH looking solely after Cancer program. Then PC obviously will be one of the components of the program and then and the program could may be take it to the higher authorities and then initiate a dialogue on ensuring because there are a good number of bottlenecks. If you look at our current procurement system, it is an annual procurement system and however perfect our estimation of opioid analgesics is, you can actually never be able to ensure the continuous availability because if the patient needs it, that’s it, we have to fulfil his/her needs. If there is an unexpected increase in the number of patients then there you have it. So you will not be able to meet their needs because you will not be able to buy ad hoc supplies. So I think we really need to have an overarching plan to solve this issue of improving the availability of opioid analgesics.

So we do have rooms to improve, thats what you are saying, right?

Pharmacist

Yes, but going by our experience, before we used to see about 10% increase in the amount of opioid analgesics procured every year but with PC having initiated, what happened was that the requirement of opioid analgesics actually doubled. If we do not control it could even triple, right (asks his colleagues). So that’s why there are other confounding factors we need to also look at but the core of the issue is solving that bureaucratic hassles we usually encounter.

It is very interesting. Because, as sir mentioned, in the past due ti lack of education and awareness, physicians feared to prescribe morphine and patients continued to suffer unnecessarily, to a large extent. Now that PC has come into the picture, pain management is seen as one of the means to improve their QOL. Patients are in better position when their pain is managed whereby they can also accept that his/her disease is incurable but the pain and distress is managed. They have time to take care of other aspects before they finally die because there are other aspects which affect their QOL, maybe some family issues, official issues or spiritual issues. So that’s why pain management is so crucial. It is very encouraging to know that the stock of opioids have improved after introducing PC in Bhutan and I think we still have so much of potential to improve further.

Nurse, Medical Ward

So like for example madam was talking about morphine. So through my observation in the district hospitals I don’t know whether morphine is available or not but the store in-charge and the nurses will wait for the doctors to prescribe morphine. But as Sir (ENT Surgeon) said if we have a program then it will be good because then if the drugs are available then it won’t get expired in some hospitals. We can then mobilise the drug if it is not used in some hospitals. Having a program can regulate that I think. Because in a hospital like JDWNRH, everyone comes here, so maybe this hospital runs out of morphine first and some hospitals might be stocking it up waiting for the doctor to prescribe you know. So if there is a proper program then there can be proper channelization I think. And that can help patients where it is needed the most.

I think district hospitals do have morphine. I have been conducting FG in the districts as well where pharmacist were included as well and it is only today that our pharmacist here saying that we might have to review and revise the narcotic regulation. I think they (Pharmacists in the districts) were comfortable because they had stock and maybe they did not have enough patients. So they all seemed to be comfortable with the regulations at the moment. But the scenario is different here (JDWNRH) as D (nurse, medical ward) mentioned. Patients are drastically more here compared to the districts. And if we are to integrate PC, as sir mentioned, policies and regulations really needs to be re-looked, right?

Pharmacist

Can I add a point here?

Yes please.

Pharmacist

I think when it comes to opioid analgesics, not only opioid analgesics but other analgesics required for PC, I think we have a long way to go actually. Coming back to opioid analgesics we are stocked with oral morphine but I think we could explore about other formulations. There are noble drug delivery systems being tried in other countries. So if people in other advanced countries can have them why not us? If it is going to make a bit of a difference in the patients I think we should be in a position to explore noble drug delivery system which will ease the pain and help patient to improve their QOL.

What is noble drug delivery system? I am hearing it for the first time.

Pharmacist

Noble drug delivery system is like there are machines which can actually deliver doses of analgesics according to the patient’s need. It is like injectable. There are such drug delivery systems being tried out. And also in terms of other opioid analgesics. There are other options of opioid analgesics that we can try out. Then we might also have to diversify the opioid analgesic basket in our formulary. So there are other opioid analgesics which we could offer to our patients.

So noble drug delivery system is it basically a parenteral drugs given through a device? Or

Pharmacist

Yes, it is like giving through a syringe pump you know.

O ok. So we can train people and give that right?

ENT Surgeon

And plus as T (Pharmacist) mentioned I think we can explore other alternatives. For example, right now we are just considering morphine, in Bangladesh there are three to four companies which manufactures these opioids and I think the regulations is not as strict as for morphine. They are less strict and it is easily available. So I think we should have those options in mind if we really want to ensure the supply continuity. And also not just morphine, now all over the world they are all going in a big wave on cannabis. For terminally ill patients cannabis has become a big thing. In Bhutan cannabis is available everywhere, why can’t we explore that? So that we don’t have to depend, you know. We can make regulations, we can, you know, tab our own potential you know. Ultimately if our terminally ill patients benefit out of it, if they can die peacefully I think we should explore other alternatives also and not just concentrate on morphine.

I absolutely agree with Sir. And so we definitely have potential for better pain management and PC, if we explore more, right? I think we all now feel the importance of PC.

Now can we discuss about education and training our health fraternity, the policy makers and the public? Because we definitely need to create awareness, right?

ENT Surgeon

For me, personally I feel now, since we already have the data of the Bhutan Cancer Registry, yet to be published, but the data is showing enough cancer burden in the country which will keep on rising. So first thing, as I said, an individual cannot do. It has to be a team work. It has to start from the MoH. First thing is to create a dedicated program for cancer by the MoH. Because if we have a cancer control program, right now the good news is, we are already drafting the cancer control policy. We have already revised for three or four times. Now it is at the final stage. We just need to input some data from the cancer registry and then the document is ready. And once that is endorsed we have already mentioned everything. So that policy should come through. In that document we have included everything, we have included about PC as one of the important part in that policy document and that is the national cancer control policy. So in that policy we have already put all our data together and we have said ‘look now we have to do something about cancer’ and the first thing is to create a program which is capable of looking after cancer, a dedicated program in the MoH with a dedicated program manager who knows what he/she is doing and a national technical advisory group for cancer control. So if we can create that program then definitely things will fall into place because PC is a very important component of any cancer control program activities. So that will take care of PC. But if we go alone as PC it won’t happen much. So it has to be in a system and as I said and to make it more system oriented we need to have program which will not only look after PC but also developing about other cancer services to improve our cancer services, train our health care providers right from the Basic Health Unit right up to the national centre for cancer excellence. All levels of people should be trained. So all these things should come as a package I feel.

And besides cancer there are other NCDs which are also a growing concern in Bhutan. As CKD is one of the problems and others like lung disease, heart disease, and liver cirrhosis is the major killer. We all know how much trauma is there in the family right when a person dies with cirrhosis, right? We can still improve the lives of those patients and families, right?

Now, PC is a holistic approach that involves multidisciplinary approach. How can we ensure to deliver that holistic approach or that team to provide holistic care in Bhutan?

Nurse, Medical Ward

Actually the answer is here (meaning this group). I think by adding a *Lama* (religious leader) and an administrative officer who can solve all the issues. What I mean is we have to work in team as a multidisciplinary approach. We all need to work together as doctors, nurses and other disciplines. Only thing is we have to work to create a group and I think Ministry is one answer as Sir mentioned. I think if we work in group we can provide holistic approach of care.

Pharmacist

I think we should also look at the patients’ needs. We should look at the angle of what the patients would like to have, what the patient wishes to have during that stage of his life. If you really look at our social angle then you would see that a patient would ultimately want to die at home. If the patient has come from a far flung area like Pemagatshel (a district in the far eastern Bhutan), he would like to go back to Pemagatshel and die. So that’s why I think we should look at actually training health care workers till the level of BHU on maybe a basic palliative care. I think it doesn’t entail much, it is mainly about how to use opioid analgesics and making those opioid analgesics available and also catering to social and emotional needs of the patients. So if we can spread out the PC service then I think it will really make a difference to that patient because the patients coming from far flung areas would not really want to die here. It would not be a dignified death for him when he/she is dying away from a place which is quite foreign to him/her. So that’s why if you can really spread out the service and even make our HCPs at the BHU level competent to cater to the needs of the patients. Then I think it will a big step.

I think that’s very important. Thank you very much. Palliative care is patient and family focussed and as T (pharmacist) has mentioned patients, studies have found that majority of patients in developed countries want to die in their home. But only a small percent are actually able to die at home. And the rest dies in the big hospitals, in the ICUs. It may be similar here in our context as well. Many in the ICUs may not really need to be there you know. So yes, education for the HCPs and the public is crucial. Now, how should we go about? When should we start creating awareness to the public?

ENT Surgeon

Awareness to the public has to be timed because we have to be ready first. Without being prepared ourselves there is no point in raising awareness because after raising the awareness we have to be in a position to intervene and do something. So first we should make our system strong. First we should make PC recognised as a discipline. Right now it is not even recognised as a discipline forget about awareness. In all other countries where cancer has taken as a big toll, PC is a recognised specialty but in Bhutan, no. We do not have a single PC specialist so far. So we really need to, first of all, built our own capacity. We should be able to handle and then only awareness should begin. First of all the policy makers should be involved in order to have PC system in place. Then gradually we can build a strong workforce. Now His Majesty has already commanded during the Desuup (the guardians of peace volunteers) training that Desuups should be trained. So now we are already working on a course for Desuups. We will cover all the 20 districts but we are doing in a phase wise manner. So we will club three or four districts and conduct one course. So from each district we are identifying about five or six Desuups out of which two will be with a health background. So we are going to train them on, you know, on basic PC where even the availability of drugs and prescribing and all will be sensitised to them. That should actually trickle down to all the districts and to the level of community.

That's very encouraging. Volunteers play a significant role in PC. I think we in Bhutan have lot of opportunities for PC or advantages you know. We have such a compassionate King and government led by a doctor Prime Minister.

Now can we discuss about the role of Drungtshos in PC? As modern physicians and clinicians what is your opinion on the role of Drungtshos in PC where we aim to improve the QOL of patients and families?

ENT Surgeon

As far as PC is concerned, because as far as curative is concerned I am not a big fan of Traditional Medicine, to be really honest (*laughs*). But when it comes to PC, yes because as we have already discussed, PC is customizing whatever we can do to the individual patient and right now our culture is such that our general population have a huge faith equally given to the Traditional Medicine. So we should give people the opportunity, especially when they are, you know, in a terminal stage. We should give them opportunity to choose what is good for them. And if the patient is satisfied, as we know that psychological component is very important in PC. If psychologically they are satisfied and they are willing to take up other alternatives, maybe traditional medicine, why not? I think there is no harm in integrating the help of Traditional medicine into PC system.

I also did a FG with Drungtshos and they were more in favour of helping the patients with psychological and spiritual aspects. They see their role in PC and they look forward to being a part of the team actually in helping the patients and families. I thought that was important to discuss here. In other hospitals I included Drungtshos in the group with doctors and everyone but in Thimphu we decided to do a separate FGD with Drungtshos at the Traditional Hospital.

Pharmacist

I think it is ultimately the patient, what the patient wants. So if he/she wishes to see a traditional medicine practitioner then I think the option should be left open.

What do you say Sir (nephrologist)?

I think as doctor (ENT Surgeon) has already pointed out, because it is dealing with someone who has a terminal illness and if it is especially their psychological aspect that is being addressed, I think there is no harm in including traditional medicine in the team.

Physiotherapist

Yes, we have to respect the monopoly of the patient.

Nurse, Medical Ward

What I feel is also the same because once the patient, we do not know now because we are not one of them (patients) (laughs). For example, as patients, I think we would explore ways to find what would benefit and we would listen to others’ opinion, like ‘he says that is better.’ So I think the option should be kept open because I think that won’t harm even if that doesn’t help also. That is what I felt.

Thank you.

Is there anything else besides what we have discussed so far? Anything pertinent that you wanted to discuss and did not come in the discussion?

Pharmacist

I think most of the aspects were covered. So I think it was comprehensive.

ENT surgeon

I just want to say, personally I am very happy about what you are doing. The topic is very pertinent and you are going to basically customize PC to our own setting which means bringing PC to Bhutanese system I mean Bhutanese setting which is very good. All of us who are here believe that PC has to be customized. Now your project is on making PC in a Bhutanese perspective and since all of agree that PC has to be based on individualised kind of treatment, you know, individualised approach, I think we are going in a right direction. So as long as the system falls into place for PC, as long as people are, you know, trained in a systemic way, as long as awareness is made in a gradual way, I think we are moving in a right tract.

Thank you very much Sir

This project is aimed to develop PC for Bhutan and the information that I have gathered from the patients, families and health care professionals are going to be invaluable.

Do you have any comments, suggestions, or advice to me as a researcher on this project?

Sir (Nephrologist) do you have anything to say? I am so happy I could recruit you because CKD is a growing issue in Bhutan.

Nephrologist

I don’t think I have anything specific to say but the project you are doing is a great project. I think hopefully it will make a big aah..sort of breakthrough impact in PC services in Bhutan. So my only thing is I wish you good luck.

Thank you very much Sir. It means a lot to me.

Anything from anyone.

ENT surgeon

I think that you are going on a right direction and through your hard work and dedication we also want to, at the end, we want to know aah.. the expectations of PC from a Bhutanese patients. So if you can give us that evidence, you know, because basically you are trying to find out how PC system can be delivered defferently in Bhutan. So for that you need to know the patient’s expectation. Expectation maybe based on so many factors. So if you can give us, at the end of your project, a Bhutanese perspectives, expectations, you know, especially with regard to a, you know, PC patient. What they would actually expect from a PC team. So if that can be made available then it will be, as care givers, you know, we will be in a better position to respond to the patient.

Thank you Sir. The needs assessment including the interviews on patient's lived experiences should help us understand what their needs and expectations are.

Pharmacist

Aah.. I think you would have already thought of a model by now because you have interviewed quite a good number of stakeholders involved in PC. So my only suggestion would be it will be good if you can keep it (the model) simple, implementable and something which can be actually taken to the community so that it will have a far reaching impact and your model becomes a success. You would have already considered a Bhutanese context, you would have considered the social aah.. socio-economic factors involved and also the geographical terrain involved and other factors involved. So if you have incorporated these factors then my only suggestion is that please keep it simple so that it is easier to implement.

Thank you so much Sir. This is a great advice.

ENT surgeon

And just to add on T (Pharmacist) who has mentioned a very important point about socio-economic status. Because that is very important. In our perspective, in a developing country, most of the people, I mean we all go through economic hardships I think. That socio-economic hardship you have to really aah… I mean you have to really reflect properly in your whatever model you are going to develop because just through my experience when I was in Kolkata the reason why I raised fund for the cancer patients is, everything is spent by the government, you know. Patients they just have to sign the bill but when I was there, there were patients who, on their own, could not even afford Rs 300 per day. So that is the reason why I started the fundraising. I still have money in the bank, I am still sending to Kolkata, some of my friends are still donating. So just because, it is not just relieving the pain. As we have all discussed it is all about the approaching in a wholesome, you know, approach but that socio-economic factor has to be really looked at least by tying up to some agency, some donor agency, where at least you know if somebody is really economically hard and you know. Because for some, for me, for somebody Nu. 1000 may not make any difference but for the other person it makes hell lot of a difference, especially when you are in a situation where you don’t have much hope, you know, your days are counted. So when he/she has to take the stress of that economic burden that even adds on to the patient’s suffering. So that in your model, I don’t know there may be lots of ways to tackle that but if that socio-economic parameter can be reflected strongly in your model I think, especially in our situation, it can be really helpful.

Thank you very much Sir. This is very helpful and I will do my best as advised.

Anything else, sister (nurse, ICU) do you have anything to say?

Nurse, ICU

Nothing as such

Brother (Nurse medical ward), anything?

Nothing

Thank you very much everyone. Thank you for making this possible despite your very busy schedule.
