# Supplementary material for: Healthcare professionals’ views on how palliative care should be delivered in Bhutan: A qualitative study
Source: PLOS Glob Public Health. 2022 Dec 12;2(12):e0000775. doi: 10.1371/journal.pgph.0000775 (PMC10021767; doi:10.1371/journal.pgph.0000775)
Supplement: S15 Data — (DOCX) [file pgph.0000775.s016.docx]

**Transcript for FGD with Palliative Home Care group in JDWNRH on 17/7/2019**

| Participant 1 | Doctor, Home care Group |
| --- | --- |
| Participant 2 | Nurse, Home care Group |
| Participant 3 | Nurse, Home care Group |
| Participant 4 | PC Nurse, Oncology Ward |

**At the very outset I would like to thank each one of you for agreeing to be part of this study. I thought this group is very important for the project. You all are the pioneers in starting palliative care service in Bhutan and I thought I will do a separate focus group discussion with the group to mainly understand what your experiencer are after being part of the palliative care team and what are the challenges, how you would like to improve things you know. So thank you once again.**

**To start with maybe we will briefly discuss on what motivated you to participate in this discussion. What made you feel like to take part in this discussion?**

Doctor:

So thank you for inviting me to be a part of your study. This is the first kind of study that is thought regarding palliative care in Bhutan. Till now in Bhutan I haven’t seen any studies related to palliative care. Although palliative care was started a year back but we haven’t done any studies related to palliative care. So what motivated me was I saw you doing palliative project related to Bhutan I found it very interesting. It will be very useful in future especially when we start palliative care in Bhutan. So I found it very interesting and so many advantages in future also.

**Thank you Sir.**

Nurse 1:

Thank you for this opportunity. Aah… palliative care was introduced to me accidentally. I was sent for the workshop and since then I have been inspired to learn more about palliative care. So I found this discussion an opportunity to learn more (on palliative care) and to participate and aah…and I found it very interesting as well. So I decided to participate in this discussion.

**OK. Thank you. Which workshop did you attend?**

Nurse 1:

It was a Training of trainers workshop by the Singaporean group here in Thimphu.

**O ok. That’s interesting.**

Nurse 2:

Since we are very new to palliative care because earlier in Bhutan there was no palliative care services. In the beginning I also didn’t know much about what is palliative care. I thought palliative care is only giving care to the terminal cases. But later I understood palliative care is very vast. Palliative care covers the psychological, social, financial, spiritual aspects of the patient. Palliative care started since 2018 (in Bhutan). This service is very interesting and is beneficial for those patients who are suffering from advanced cancer. At the moment our service is only for the advanced cancer patients. That is how it is started in Bhutan because at the moment we don’t have so much of knowledge and experience. I think this is very important for all other patients as well. Regarding the health workers we are not well trained. We only had workshop for ten days from Kerala. So with ten days of training we are posted in the home care group which is very interesting. At the beginning there was not so much of support and still we are waiting for the approval so that we can expand the service and give more care to the patients. Just now we are doing home based palliative care for patients who cannot come to the hospital on a regular basis and we provide symptom management which really benefits the patients. This really reduces bed occupancy rates in the hospital and this even makes patients and families comfortable and they find it beneficial and that’s how I am motivated to take part in this discussion and we are very happy (smiles) when we get such opportunity because we can then get to expand our knowledge.

**Thank you sister.**

Participant 3:

Before when I was working in the ward I did not have any idea about what is palliative care so we used to give care to the patient and we used to do the procedures but we did not have idea what palliative care is. Before starting palliative care service we mainly focused on procedures and we used to talk with the patients but palliative care includes all the domains like physical symptom management, psychosocial and spiritual care. So we can use our palliative care knowledge everywhere like not only in the hospital but outside the hospital also. So after starting this palliative care I have learned many things and we can have patients in many ways.

**Thank you everyone. It is very encouraging to listen to your motivations.**

**Now the next discussion is after you have formed the team and started visiting patients at home, what are your experiences, what are the challenges and how do you see the future of palliative care in Bhutan as pioneers?**

Doctor:

Actually what inspired me to be in the palliative care team is from my own personal experience with my own mother. Since she was diagnosed with an advanced cancer of cervix so I saw her suffering from the side effects of radiotherapy and chemotherapy. At that time I wanted to do more but I could not do since I was just starting with my MBBS. Since I joined here one year ago I was asked to visit patients homes for home care by Dr. T (Gastro-oncologist). So for the first time I visited patient. What I felt was, wow, why we haven’t done this type of service before? Why for the last ten years resources has not been used for this kind of service? Why were doctors not involved in this type of service? Patients were happy, families were happy, communities were happy. Even we are also satisfied. So I felt so happy to be part of it. While we are going for home visit we came across so many challenges also. Some can be solved but some cannot be solved. One most important challenges I felt was dying at home. So difficult for the patient party. We can manage pain and other symptom at home but at the time of death there are so many obstacles for them to die at home. One reason could be because they don’t have their own home.

**Ok. So they are in a rented apartment?**

Doctor:

They are in a rented house and they cannot die in that house because the owners doesn’t allow death to happen in their house. Next thing is the funeral service in Bhutan. If they come to hospital it is a direct process. So if they die in the hospital they will be directly taken to the funeral service but at home there are so many cultural and social issues which makes it very difficult to die at home. So most of our palliative patients are admitted here at the time of death. Actually from our side we want them to die at home surrounded by their loved ones but that is not always practical.

**Sir, do you think those patients wanted to die at home given an opportunity?**

Doctor:

Yes, they want to but they are not able to.

**Because there are so many literature which says that people actually prefer to die at home but majority land up dying in the hospital. And this could be one of the reasons**

**Any other experiences? Do you (Nurse 1) also go to the homes?**

Nurse 1:

No, I take care of patients in the ward.

**What are your experiences in taking care of palliative care patients in the ward?**

Nurse 1:

After I got some training on palliative care it really improved my clinical practice. For example if I give an example of pain, before the training the pain was just the pain and after the training I knew that pain is at the top and it has got so many branches and we got to focus on all these branches and the pain is addressed. For example pain can be due to physical pain, emotional pain, any pain. So after training I am a better aah.. I do better assessment of the pain and I report to the physician in a better way and patient gets a better service and not only that the other thing is like other symptoms like psychosocial issues I go to the patient, talk to them and aah… I get to address them in a holistic way. And I do face challenges like the first thing is always the time constraint. Given the number of patients and number of beds and staff. For example if we consider about OPD chemotherapy patients we just get time to give medications and talk little bit, introduce. So if I am given a choice I would like to sit beside the patient, talk with them and ask them few questions and interact. But the current situation is like we can just give them basic services like give them chemotherapy and send back. So these are the challenges.

**What about the training? Do you think you need more training on palliative care?**

Nurse 2:

Yes, definitely. I feel I am immature in palliative care but still then I feel that I can do a lot if I have little more time and resources but I definitely feel that I need more training in palliative care.

**What has been your experiences sisters (Nurse 2 and 3) having visited patients’ home till now since last one year? How is your experience compared to your past experience, in taking care of patients with advanced illness in the ward, prior to geting into palliative care? I know sister (Nurse 2) worked in the ICU, right? How is it different when you started going to patients' homes to provide care?**

Nurse 2:

After going to the patient’s home to take care of them I found that there is a vast difference in taking care of the patient because in the ICU and in the ward I usually managed acute cases with lifesaving and very aggressive treatment but in palliative care especially we need to have compassion and understand well their problems like family problems, patient’s problems like financial, social, spiritual and everything and I experienced that palliative care service really need time and mind like to really focus and understand on patient’s problems and in the ward we usually get one to two minutes to talk with the patient and we don’t get enough time to understand them. We usually focus on the main treatment and in palliative care we have a chance to understand the patient’s feelings, pain and everything. We get chance to be satisfied and really understand the problem. If we make them happy we ourselves feel happy. For example while caring for an advanced cancer patient, when we say cancer the main priority of managing the symptom is the pain. Pain is the real problem and it limits the patient from everything. If the pain is not controlled patient usually get deteriorated and they cannot take food and if pain is not controlled there is no quality of life. After coming into palliative care I understood that pain management is really important and medication is really important. To educate the patients and the families to take medicines round the clock to control pain. Before I came into palliative care I didn’t know about pain management because in the wards cancer patients are usually under treated for their pain and they do not get adequate pain relief and the quality of life will get worse. Even for the advanced cancer patient even if there is no cure if we control pain and control the symptom then even for few days patient can live happily, there is quality of life and patients are so much thankful. This is my experience (smiles) that palliative care is very important. It is mainly time that we need to spend with the patient and we get so much of satisfaction. Even we develop relation with the patient. They call us and share all their problem, they seek help any time.

**How do you feel when they call you in the middle of the night saying you know ‘my family member is dying, what should I do?’ How is it? Do you receive such calls?**

Nurse 2:

When we receive such call we get little bit disturbed but then we also feel the importance of attending but till now we don’t have the service. At the moment we only have scheduled visits and we just advise them what to do and where to go if they need help at night or during holidays. We direct them to emergency. We just advise them which doctor to see and whom to consult. Because patients are often confused whom to consult and where to go when the situation itself is very sad (smiles) and distressed.

Nurse 3:

When we visit patients we see so many things, different situations and we can like find out the possible solutions. In some cases they have many problem but although we cannot solve all the problems like whatever we can we do try to solve their problems even like after explaining their disease condition if they have wishes to go to their village we can explain and we can facilitate and allow them to go to their village. Then we face many challenges also while at home because they have lots of questions and we can answer some of the questions but some questions are very difficult to answer. We do not know how to explain and how to talk and communicate. It is very difficult. Some families when their patients are at the last stage and are not able to eat anything thing, they request for IV fluids. For that we will have tough time explaining that and the patients party also they feel like what to say? Even if we explain they will have tough time to accept because we are not able to convince them.

**Interesting. It is a difficult topic, right? A hard topic. Umm…you did mention about some of the challenges. What are the main needs in these patients at home? Besides physical pain, because physical pain is easy to identify, right? So besides physical pain what are some of the other needs in the situation where there is an advanced illness?**

Doctor:

So needs can be physical, psychosocial. So from my experience I found physical need was more important. Basically, one of the physical needs was nutrition. So most of the time patients and families complain about poor intake. So at the time of last stage our only hope is to counsel. Giving parenteral nutrition to patients with advanced illness is not useful. Even if we counsel family members to give frequent meals they are not so much convinced because of so many expectations. So nutrition is one of the basic needs that is very difficult to address in both patients and patient parties. So apart from pain, nutrition is one.

**Ok. At the moment it is just the doctors and nurses in the team, right?**

Doctor:

Yes

**You all have made history of palliative care in Bhutan.**

**There may be patients who are spiritually and socially distressed. How do you manage the socially or spiritually distressed patients at the moment?**

Doctor:

In that situation it is very difficult to manage. While we are mostly trained in the physical needs so dealing with especially social needs is very difficult. Still from our side, apart from spiritual, since Bhutan is a spiritual country so I don’t think (Smiles) spiritual need is so much of a concern to them, social part is very difficult to deal.

Nurse 2:

Especially when some patients do not get support from the family. They are lonely you know. Some patients do not have any one to take care of them. Some are divorced and have only their young children. We used to face such problems and we used to solve through, luckily we have Bhutan cancer society (BCS), so we consult them. So far we have four or five cases that we have supported financially from BCS and also from Her Royal Highness Ashi Kezang Wangmo’s (*Our King's aunt who is the patron of PC in Bhutan. Ashi Kezang often donates fund for the welfare of poor patients and has also recently donated in developing palliative care in the national referral hospital*) donation. We consult BCS through our nursing superintendent and we get some fund of about Nu. 3000, that is once only and not continuously (smiles) but, to support the patient till the treatment is complete. When we get this support I feel that patients are psychologically well and mentally they are very happy. Some patients become physical better and they go home as well. So usually patients become better once when they get social and financial support.

Other challenge is that, most of the time the family members want us to conceal telling the truth to the patient. They don’t want us to tell the truth (the diagnosis). In that case we face difficulties to manage at home because patients do not get satisfaction, patients do not know the diagnosis and that they are not going to get cured. So patients expects lots from us like ‘if I am not getting better why I am kept at home (smiles)’ and at the end they are even reluctant to take pain medications because they know that it is only a pain medication that is given and they are not satisfied. They want further treatment. Some say ‘if there is no treatment here why they are not referring me outside?’ So they have lots of hope and it is sad that we have to listen to the family members and we have to deal according to the family members’ decision. I feel that families are doing this because of the lack of knowledge because patients with advance illness need to go through a long journey of treatment that they have to follow. It is not once or twice but they have to go months and months and even years. So if they are not told the truth at the end patients get frustrated. After getting chemotherapy and radiotherapy which are very painful patients hope that these treatment will help them feel better but instead they are getting lots of side effects from chemotherapy and all. Once they start deteriorating their health patients usually start losing trust with us (smiles) as a health worker and they are really reluctant to develop relationship. So in this regard if the family are not educated about the truth telling and make them understand I think in future our palliative care at home can be affected. Because in order to provide palliative care we need a team including family, health worker and if we discuss the problem around the patient and if we allow them to take the decision that will be better. I think the patient will be happier. But when families do not allow that is little bit of challenge to make them happy at home because of lack of knowledge. The health worker should be trained, you know, to deal with such case we need more training and skill and then we have to deal with patiently and cooperate with the team.

So are you saying that you ave limitated knowledge and skills in helping the families to understand how important it is to let the patients know their diagnosis?

Nurse 2:

Yes, because some patients like 50% of patients know the diagnosis and other 50% they do not know because families do not let them know. When we compare these patients the patients who know are easy to visit. They can take decisions and we can ask the patients to express his wishes. There are many wishes after medical treatment is no more useful like spiritual wishes like going to hot springs, holy water therapy, to the Lamas. That way patients are at peace and some patients even go back to their village and they are actually much better and improved.

**How nice. So it is interesting that you have identified those gaps and you also identified the importance of trainings, right? So at the moment the group consists of doctors and nurses, who else would you recommend to be in the home care team?**

Doctor

Firstly I will include nutritionist to give counselling especially regarding the nutrition. Second person will be physiotherapist. I would definitely recommend physiotherapist because of so many cases we have with bed sores, patients with lung disease so these patients really need physiotherapy. Third person will be one spiritual person. It is not for them to give directions but what I really want from them is the counselling to the patients and not really the prayers like there are these long life prayers.

Nurse 2:

When it comes to spirituality I think we need to understand more as every individual has different beliefs. What we do is we usually respect what they believe and if they like to do anything we allow them to do. It is very difficult to advise them what to do and what not to do (smiles)

**You are right and palliative care is anyway patient-and family -focussed, right? Care has to be according to their needs. And before I forget, why are nutritionist and physiotherapist not in the team right now? Because that’s not very difficult. There are nutritionists and physiotherapists at JDWNRH.**

Doctor:

So we haven’t formed the team yet.

Nurse 2:

That is one and the other is that it is not yet approved also (laughs)

**You mean it is not approved by Ministry of Health?**

Doctor:

Yes, by the Ministry of Health. This home care group is actually started by the president of JDWNRH (Jigme Dorji Wangchuck National Referral Hospital). So since it is not yet approved by the Ministry we could not do anything further although we really want to.

Nurse 2:

Once it gets approved I think we really need to expand.

Doctor:

We really want to take palliative care forward.

Especially we need to get the consent from the highest monastic body to involve the spiritual person to be part of our team.

Nurse 2

It is quite complicated (laughs)

**Do you see the role of Drungtshos in palliative care?**

Doctor:

I think some patients prefer to avail the services from traditional medicine when they know that there is no cure for their disease. I think Drungtshos can provide some spiritual healing which most patients wish for. So I think they do have a role in palliative care

**What do you say about the staffing? What is your advice, recommendation or opinion about the staff if we are to start formal palliative care service? What is the future of palliative care in terms of staffing?**

Doctor:

If we get approval from the Ministry of Health then definitely there is a bright future. Our palliative care patron is HRH (Her Royal Highness) Ashi Kezang Wangmo Wangchuck (The King's Aunt). So in future especially in the team we definitely need a palliative care specialist. That specialist should not only look after cancer patient but also medical and all other general patients with advanced illnesses. But to help him he needs staff under him. There should be nurses who are trained in palliative care. And even nutritionists, physiotherapists, counsellors, psychologists, social workers, volunteers. With that specialist definitely he/she will be training more in future.

**So the bottleneck right now is the approval not given from the ministry, is that right? Why do you think the approval is withheld? You have any idea?**

Doctor:

I am not really sure why it is not approved but what Ministry wants is to have palliative care all over the country. All twenty dzongkhags (districts) should have palliative care as per the Ministry but since in Thimphu itself we could not start, although we have started but we could not progress. So the ministry wants us to develop palliative care in all over the country.

**Then only they will approve you mean?**

Doctor:

Yes. But if we start from all over the country then we definitely need staff, resources. Then only they will approve.

Nurse 3:

This is the reason we heard that approval is withheld.

**But thats not easy. And they haven’t approved in the national referral hospital where it will develop further and further how can it be available in whole of the country? That’s my concern. Anyway that’s beyond our scope here, right?**

**Sir (doctor), since you are the physician in the team what do you say about the availability and accessibility of drugs, the palliative care essential drugs? Particularly pain analgesics, like opioids. What is the state of analgesics here in JDWNRH?**

Doctor:

So till now since I have managed so many patients, so even if I prescribe for two months it is dispensed for only one month from the dispensary (pharmacy). I don’t know why they dispense only for a month. But till now I haven’t found any shortage of medicines. And the good news is that we are soon getting 20 long term morphine titrate.

**Twenty? You mean 20 mg?**

Doctor:

Morphine 20 mg and 15 mg slow release. We are soon getting these supply which will be very good for our palliative care patients.

**That’s very good news.**

Doctor:

I think Fentanyl also but it is a bit expensive.

But Morphine is cheap but very effective, right?. I have also been talking to the pharmacists in the regional referral and district hospitals but then at least in Bhutan even if you have no palliative care service when it comes to drugs there is not much of issue they said. Do you agree?

Doctor:

Yes, I agree, there is not much issue

**So that’s a very good news, right? Because even if we have trained physicians and have a committed team if the drugs are limited then we are limited, right? So thats not so much an issue here, right?**

Doctor:

Yes

**So to summarize, we have covered on your experiences and before that I understood what your motivation was to be part of the project. Then your experiences and your challenges. So the main challenges are having less number of people in the team, not having adequate training, and family members not knowing the importance of letting the patients know the truth and that’s because the families themselves not having adequate education, right? And manpower obviously required but at the moment the bottleneck is the approval from the Ministry of Health. Is that right?**

All participants:

Yes

**Who do you think will help you get through this approval?**

Doctor:

Actually I think the approval is underway.

Nurse 2:

There is nothing in written. They only say verbally ok, ok.

**I am planning to present my proposal at the ministry. I thought I will present because I want to appraise about the project and emphasize the importance of palliative care.**

Doctor:

It will be very good. Better to invite high level people.

**I was thinking the same because Lyonpo (Minister) is very interested. She told me palliative care is very close to her heart. Once Lyonpo is committed others will naturally fall in place. So that’s why I thought I will make a presentation to let ministry know what is happening in palliative care. Because we all know palliative care is important and in the long run it is definitely going to cut down the cost of the health care service. That’s there in every literature. Initially may be for training and starting it may be expensive but then in the long run it will be an investment. It will reduce number of patients in the ER if we have good palliative care service, palliative care can also reduce bed occupancy rate and also so many other unnecessary investigations and drugs and even referrals abroad.**

**So now that you know what this project involves, as a pioneer team in the country, what is your advice to me? Because this study will bring about lots of evidences and I am hoping that this project will be a baseline information for all of us and I am involving as many experts besides my supervisors who also help me get connected to every expert which is very helpful. What do you want me to take from this discussion so that it has an impact on the team here and the new palliative care service in Bhutan? Any suggestions, comments or advice.**

Doctor:

Since it will be the first resource on palliative care so now we will have scientific evidence to prove that palliative care is happening in Bhutan. So what I want you to emphasize is not only present to ministry but if you can put up in journal. Soon there will be some conference. I think you will be here.

Not this year but next year I am hoping that I will be able to present in the University conference. I will anyway be coming back next year for phase II data collection where I will be involving the relevant stakeholders like the policy makers, the parliamentarians, the drug agency, the civil society organizations like cancer society, kidney foundation, Lhaksam, and the clinicians here, the administrators, and many others. I am hoping that I will be able to bring all of them together. That will be another big challenge because this year is on the first phase which is palliative care needs assessment among patients, families and health care providers. Next year, in the second phase. it will be presenting the findings of this year, educating the stakeholders on what is palliative care and asking their opinion and finally coming to a consensus on a suitable palliative care framework for Bhutan.

Doctor:

So if you could write a very good review about the scientific journal. Through your research it will be very beneficial for us.

Nurse 2:

It will help us to know about appropriate palliative care for Bhutan

Doctor:

To pursue further definitely we will need scientific evidence on palliative care. Through your work we put evidence to the Ministry of Health.

**Sure. That’s very encouraging. You are very small but very committed and a very passionate team. Thank you for your time and thank you very much for the information you have shared and I look forward to working together in future.**

**Is there anything else you wanted to discuss besides what has been already discussed?**

Participants:

*Nothing..*

**So nothing, right? Thank you everyone.**
