# Supplementary material for: Healthcare professionals’ views on how palliative care should be delivered in Bhutan: A qualitative study
Source: PLOS Glob Public Health. 2022 Dec 12;2(12):e0000775. doi: 10.1371/journal.pgph.0000775 (PMC10021767; doi:10.1371/journal.pgph.0000775)
Supplement: S16 Data — (DOCX) [file pgph.0000775.s017.docx]

**Field note for FGD with Home Palliative Care Group JDWNRH**

Date: 17/7/2019

Venue: Oncology Ward

The palliative home care group was formed in 2018 in the national referral hospital and they cater to patients with advanced cancer at home in the nearby community in Thimphu. Their main focus at the moment is physical pain management. I went with the group in two of their home visits and learned that they are providing the best possible service at this stage, where palliative care is a new concept, despite so many challenges.

A separate focus group discussion was planned with this group besides another group in JDWNRH consisting of specialists, pharmacist, physiotherapist and nurses. This discussion was mainly to understand their experiences of rendering home care service and the challenges they face. The group consisted of one doctor and three nurses. The team leader of the group could not attend the discussion at the last minute due to some personal issues she had to attend to. There were four participants in the discussion group, a doctor and three nurses. One of the nurses (Nurse 1) did not belong to the home care group but he was involved in providing palliative care to patients admitted in the oncology ward where there were three beds allocated for palliative care. Having participated in the survey, he was very interested to participate in the focus group and so he was included in the group.

The discussion was fruitful and all four participants were interested and motivated in the discussion although it was mostly the doctor and one of the nurses (Nurse 2) who talked elaborately but the others agreed to what they were saying.

From the discussion I could feel that the group had limited knowledge and skills on palliative care which is obvious because they received only ten to fourteen days training on palliative care. More training for them would further develop their knowledge and skills and that would help advance palliative care service and improve the quality of lives of patients and families they visited. All four in the group were interested, committed and passionate about palliative care.

Thank you.
