# Supplementary material for: Healthcare professionals’ views on how palliative care should be delivered in Bhutan: A qualitative study
Source: PLOS Glob Public Health. 2022 Dec 12;2(12):e0000775. doi: 10.1371/journal.pgph.0000775 (PMC10021767; doi:10.1371/journal.pgph.0000775)
Supplement: S17 Data — (DOCX) [file pgph.0000775.s018.docx]

**FGD with HCP in Rangjung BHU Grade I on 15.5.2019**

| Participant 1 | Doctor |
| --- | --- |
| Participant 2 | Nurse 1 |
| Participant 3 | Nurse 2 |
| Participant 4 | Nurse 3 |
| Participant 5 | Nurse 4 |

So I would like to thank you sir (doctor) and your staff for willing to participate in this FGD. To start with, can we discuss about your experiences so far in managing patients with advanced illness like advanced cancer or heart failure, lung failure, kidney failure or a very old patients or patients at end of life?

Doctor:

Aah… Like I said in my introduction, we do not have a separate room allocated or to be allocated for palliative care or for patients who require palliative care. We just keep them together in the general ward and then we symptomatically treat them. Aah…Those symptoms which are beyond our knowledge and our experiences we consult with our seniors and treat them and if the patient is symptomatically better and do not need much of our services we discuss with the patient parties (family members) because in our Bhutanese context most of the people like having the last phase of their life to be spent at their home so we discuss with them the need of continuous medication and need of any symptomatic treatment and then we ask them to weigh themselves and then since we do not have a separate unit we ask to decide if it is better to continue medications at home and in between if there are any emergency situations that arises we ask them to come back. Because I believe that rather than keeping them here in the single warded room it is better for those requiring end of life care to be at their own home. So we assure that their symptoms are controlled and the medications can be taken at home under the guidance of their caregivers. That’s what we usually do.

**Do you have many such patients in the community within your catchment area?**

Doctor:

Yes, aah…during my stay for the last four years in this BHU I have encountered several patients at end of life especially related to cancers. This region, the north of Trashigang, there are too many undiagnosed Hepatitis B leading to hepatocellular carcinoma. Now in our Bhutanese setup, I am not trying to say that there isn’t treatment but the only possible treatment is the test that is Trans arterial chemo embolization which is radiologically done or next thing is to be for liver transplant if possible but since we do not have this and then aah…with the clinical scenario and then the investigation procedures since we can direct towards hepatocellular carcinoma then we send for CT imaging which is now available in the eastern regional referral hospital in Mongar. We send the patients there get the specialist opinion and get the patient back and upon reaching here we just ask the patient like I just said. And then the other thing is mostly we get this end of life care for congestive cardiac failure, then interstitial lung disease, COPD with cor pulmanale and pulmonary hypertension and then currently we have aah…in the ward with neuropathic ulcers who has poor socio economic status. The wound has been there for the last two to three years and since I came here the specialists have been consulted. And she is here for daily dressing and the wound heals but then aah.. it takes a huge lot of time and so she can also be a palliative case because due to this problem her daily works are limited. These are what I can remember at the moment.

**Aah… So do you or your staff go to patients' home to care for them when they are not able to come to the hospital?**

Doctor:

So far we haven’t attended any at their home and then we have such a circular saying that none of our health workers until and unless from a higher position will attend, even our community health workers like HAs (health assistants) will not attend. If it is nearby yes, definitely but if it is a day’s travel or further then we are asked not to go.

**What could be the reason for discouraging home care? Is it the financial implications like the travel allowance or?**

Doctor:

Yes, mainly to do with the travel allowances. If the palliative care protocol comes through then this segment should also be included because yes, we are serving humanity but at times humanity should serve us also. That is what I feel. So this protocol should also incorporate those budgetary heads and those officials who deal directly not just at the grass root levels. That’s what I feel.

**Brother (Nurse 1) what is your experiences and challenges in taking care of patients with advanced illness?**

Nurse 1:

Thank you madam. I don’t know how I can answer very well with my limited knowledge *giggles…*Being a nurse we are mostly dealing with the chronic patients giving nursing care and as already our doctor has highlighted that in our area we are now getting more of such cases where we have to provide palliative care. So when the doctors admit the patients in our ward most of the time we are only dealing with or providing nursing care. So sometimes with our limited knowledge we also come across problems as we provide care to the patients. So firstly, some of the challenges we face are aah… sometimes while the patient gets admitted in the ward and have some communication with them so these type of patients are we know that they have been having this problem for a long time and so we lack the cooperation from the patient side and the patient party. And also because of our limited knowledge we also fail to give adequate information and care to them and that’s why I want to suggest one thing here that as you develop this palliative care protocols if you can include in your protocol some training programs for the nurses and health workers that is going to help and we can also provide better care and service to the patients.

**Thank you so much. Education and training is one of the objectives of this project because the aim is to develop a public health approach of palliative care model where umm…education for the health care professionals and even the general public is one of the components and also looking at the policies and reviewing the policies if required and making certain drugs that are required for palliative care available. So education and training will be one of the priorities.**

Nurse 2

I graduated from India, Tamil Nadu, and there we didn’t get much practical exposure. Theoretically we are trained well but in case of practical we were attached to a private hospital and in the private hospital patients pay for the services so we were not given much chance to practice. Unless we are well trained we cannot touch the patients. But in Bhutan the hospital is attached with the faculty of nursing and the students are trained well practically. These are the challenges and for me I have experienced with regard to palliative care when I was in Tsirang hospital (one of the district hospitals in the southern Bhutan) during my attachment after completing my course when I was waiting for the placement. So there was a terminal case with an advanced stomach cancer. As a recent graduate on duty I had to provide psychological support and some basic nursing procedures like IV infusion several times. Such patients are psychologically not well and I realized that we really need be able to provide psychological support to such patients. And here in our BHU in Rangjung we don’t have a separate room as stated by our doctor and we have to keep all our patients together in the same ward and we give nursing care to different patients according to the doctor’s order. We are at times very busy to provide nursing care because we have to follow doctor’s orders and even if the number of patients are less but on the other hand we find it very difficult as our colleague here stated we are not given much training on such specialised course.

What are some of the needs the patients who are terminally ill? Because when the patient is diagnosed with an advanced illness it is not just physical pain, right, there are so many other needs.

Doctor:

Aah… Now Rangjung BHU is strategically located, neither too remote nor urban but then we cater to various group of population from well off to the poverty line. So yes, taking care of the symptomatic part basically everyone has the same symptom but now more than that I feel like aah… once someone is diagnosed with a terminal illness aah.. there is much lack of aah..social support from their own relatives la. If one is diagnosed, if one has been productive citizen for last aah.. during his normal healthy life and then suddenly he/she is diagnosed with a terminal illness then the family part, yes, Bhutan is a Buddhist country but at times there are a sect of people that where people think that once they are unproductive it further hinders them from being productive. If the husband has say like hepatocellular carcinoma or something like gastric Ca and then the wife has to …yes, few does it because aah…aah…the main purpose of life is to help when in need but few does it and few ignores it. They just dump terminally ill patients to our ward or somewhere and they are left on their own. Then they have some aah..social responsibility also because back home at times because of the socioeconomic status they have only working individual at home and then I don’t think they have other way than to discard the patient in the ward and then leave for their works. And at times I also feel that it is their responsibility to take care of the patient aah… since they were together they should continue to take care of their near and dear ones during the illness. Now mostly people even if, yes, at times we suspect that this patient has a terminal illness which will need a lifelong treatment, even if you refer to higher centre they say that socio economically I don’t think I can because yes, due to free health services by our beloved Kings we are being given free ambulances services where we can drop till Trashigang if they wanted and the highest (at the most) we can do is till the eastern regional referral hospital in Mongar which is three to four hours’ drive from here. Yes, we can do it but now coming back from there and staying there it incurs lots of economic burden. So psychologically yes, and that if possible illnesses can be categorized and some support can be given from our government because we Bhutanese have a common notion that we ask everything from the government rather than asking what we can do for the government but for the few sect of population if we can categorize and then have some welfare, though there are so many welfare schemes like the MSTF, the Multi sectoral task force, and so on but still they do have the protocols but those protocols are different. So in view of those aah..poor patients and attendants if something is there we can provide them especially during referrals and in getting back (home) it would be helpful.

**Thank you sir.**

**Now can we discuss about your understanding on palliative care?**

Doctor:

So palliative care is I think caring for patients with terminal illness.

How about the nurses?

Nurse 1:

Taking care of terminally ill patients

Nurse 3:

Same I think, taking care of terminally ill patients

**Yes, so palliative care is a hoilistic approach of care encompassing physical, psychological, emotional and spiritual domains with a aim to improve the quality of life of patients and families affected by an advanced illness.**

**So palliative care requires multi-disciplinary approach where doctors are involved, nurses are involved and according to the needs of the patient like a physiotherapist if required can be in the team, nutritionist or dietician can be in the team and then the social worker to take care of the social aspects and then spiritual leader or a monk who can help the person spiritually, if needed. So some of you did mention about the training needs.**

Doctor:

Yes, since we have this palliative care training incorporated in our curriculum and since none of our staff including me have availed this special training on palliative care and then if at least one or two are trained from at least a district or if possible one staff nurse from every BHU is trained then he/she can disseminate his/her knowledge to others and then if possible if the structure of the BHU, if the rooms in the BHU permits, we can provide better care to the patients.

The government has a plan to give every BHU one set of hospital like a mini hospital to upgrade everything. The basic is the doctor, be it general or any one, then the staff nurse who is well trained in palliative care because I think that one particular staff should be fully designated rather than all other fields incorporated. Then he/she will take care of that ward and the rest as madam mentioned like physiotherapist, can be called as required. I don’t think they will need daily rounds or everything daily changed. So like madam said aah..culturally, spiritually if we can aah..make it such a way that they feel homely even if they stay within our health facility with inbuilt facilities if possible the household amenities can be provided that would benefit them. Starting from fooding, their sanitation taken care. Yes, it is doable la. There is not much only few will opt to stay here. Most of the people think that this is the last phase of life and prefer to stay with their siblings and other near and dear ones and have so many undone works back at home. So it is up to them to choose but we will have to give the options that such things can be provided to them and it is up to them to decide because we can’t force anyone or forcefully ask patients and patient parties to directly dump here or stay here.

**What are some of the analgesics that are available here (BHU Grade I) and what are the challenges with the current supply of drugs especially when it comes to treating pain in terminally ill patients?**

Doctor:

Ok. Regarding the drugs that means the medicines required for symptomatic care, we are provided near or equal to the district hospital. Only thing we don’t have is injectable morphine but we have tablet morphine. We have pethidine, we have tramadol injection but we don’t have capsule tramadol. These are the main things we have.

**Thats good**

**When you have terminally ill patients in the ward and if the patient wish to perform some religious rituals in the BHU, do you allow them to do, or you do not allow or is it difficult or how do you deal?**

Doctor:

Now since we do not have a separate room we don’t usually allow them to perform in the BHU but we give a separate letter of consent, a letter of understanding that they are are allowed to take the patient home for a ritual for these many hours and will be returning before the next medication starts. That’s how it is being done. We don’t allow any rituals where they need to produce smoke or we even don’t allow other simple rituals which requires *drebu* and *trantrin* (bells that produces noise) which will disturb others’ wellbeing and their peace of mind. Then if they really want we ask them to take leave and return at the exact time mentioned because once they are admitted it is our responsibility that if something happens we are accountable.

**Right. How do you see the role of Drungtshos in palliative care?**

Nurse 1:

I think many patients like to go to Drungtshos when they don't get cured. I think Traditional medicine will be helpful in palliative care.

Doctor

In Bhutan I think the other option for patients with advanced illness is traditional medicine. I think they can give psychological and spiritual healing and patients may get satisfied. So I think it is a good idea (to involve Drungtshos in palliative care)

**Thank you so much. Is there anything you want to discuss besides what we have already discussed?**

**Nothing....**

**So to come to a conclusion aah… I think it is understood that this research is mainly to help develop PC model for Bhutan. You might have understood from the information form the aims and objectives. Do you have any specific comments, advice or any suggestions for me on this project?**

Doctor:

Aah..As far as I could understand from your questionnaire the palliative care, particularly in ward (inpatient) care, palliative care that we provide in the hospital if you can like allow the community workers at the community level like I said most of the people might choose their own form of the last phase of life. So if some provisions can be kept for some dos and don’ts so that we can also attend because attending to a patient a doctor has to go, a nurse has to go or someone has to go and it becomes a team then. Now when it becomes a team, suppose say like I am the only doctor in the BHU and I have around six nurses. Now if I go and attend that patient other patients who are admitted will say that in case emergency arises they will think that … or else we can designate that only a focal person is sent but then at times they might have to call back and say that such situation is there. So if you can keep some aah…space where the team can visit and I don’t feel it economically feasible for a single patient because if I go I don’t think I can do much so I will have to take two to three staff from here and then vehicle has to be arranged, everything has to be arranged where at least four to five thousand bugs will be spent. So this should also be taken care of when you are developing the protocol because developing protocol is easy but working at the ground is so much a hassle.

**I think this is a very important point. Actually palliative care is not only to be focused in the hospital. Palliative care can be reached to the community, at patients' home. It can be home based care and there has to be a team with a routine schedule to visit patients at home as required. Once a week or once in fortnight. And we will also explore on the provision for the patient and the family to call their doctor for information or advice, you know. Most of the time palliative care is not an emergency. Most of the time they will call you for certain information or some kind of advice or to inform you what is happening. Palliative care team can also train the family members, devoted family members, on how to do certain procedures like wound dressings, and provide basic care. So for all these we need supportive policies, which is one of the focus of the project. So what you have advised is very, very important. Thank you sir.**

Doctor:

Like in those era when this Hansen’s disease was so prevalent there was a system where health workers visited the particular person’s home. Not just only he gave the medication or monitored how he is compliant to medications but there was also the system where patients were given some shoes and something like cash. And forget about the shoes I have also seen Dr. Ugyen Tshomo (*a senior gyne oncologist* ) who is a aah…the first thing in palliative care was taking care of the person. Even a single flower you take she usually comes to Kala Bazar all the way just to pick a single rose, one morning I before 8.30 she came and she was picking a rose and then I was asking why was she picking this rose because she was in white coat and she told me ‘even a single rose that I take to my patient feels like it is a gift’. That’s an emotional impact. So if something like that not necessarily that we will have to give it but if something like that can benefit because there was a system when the Norwegian Mission who served in a way to show empathy and not trying to give what is not possible but they used to mobilise funds ensuring that the patients with Hansen’s disease were not alone and that they are there to support them.

**That's interesting. Dr. Ugyen has been very supportive for palliative care.**

**OK. Is there anything else you want discuss before we close it.**

Nurse 4:

I have nothing to say. My opinion is same as what our doctor has said. (*She didn’t want to say anything each time I pointed to her and I did not force her*)

Thank you very much for the participation, for the time you all have given me and the information which is going to be invaluable for the research project.

So thank you very much everyone.
