# Supplementary material for: Healthcare professionals’ views on how palliative care should be delivered in Bhutan: A qualitative study
Source: PLOS Glob Public Health. 2022 Dec 12;2(12):e0000775. doi: 10.1371/journal.pgph.0000775 (PMC10021767; doi:10.1371/journal.pgph.0000775)
Supplement: S18 Data — (DOCX) [file pgph.0000775.s019.docx]

**Field note for FDG with health care providers in Rangjung BHU Grade I**

Date: 15/5/2019

Venue: Doctor’s chamber

Rangjung BHU Grade I is under Trashigang district. The BHU has two general doctors and ten nurses altogether. One of the doctors was out of station. Focus group discussion was conducted in the doctor’s chamber in the afternoon, at 1PM, after all the patients were cleared. The participants included one doctor and four nurses including three male staff nurses and one female assistant nurse. The BHU did not have physiotherapist, pharmacist and Drungtsho. In the discussion today, the doctor was the only one who was the most participative and the nurses were not very participative. The three male nurses responded to the specific questions asked to them but a senior female assistant nurse did not want to say anything. I guessed she was not comfortable speaking in English although I had, in the beginning of the discussion, made it clear that they can speak in any language they are comfortable with like Dzongkha, Sharchopkha or Lhotshamkha.

One of the male nurse, participant 2, and the doctor seemed to be very interested in the discussion on palliative care.

Thank you
