# Supplementary material for: Healthcare professionals’ views on how palliative care should be delivered in Bhutan: A qualitative study
Source: PLOS Glob Public Health. 2022 Dec 12;2(12):e0000775. doi: 10.1371/journal.pgph.0000775 (PMC10021767; doi:10.1371/journal.pgph.0000775)
Supplement: S19 Data — (DOCX) [file pgph.0000775.s020.docx]

**Field note for FGD with HCP in Samtse Hospital**

Today on the 7^th^ of June 2019 focus group discussion was conducted with the healthcare providers of Samtse hospital. The participants included a general medical doctor, a physiotherapist, two nurses, one each from the two general wards, *Drungtsho*, the traditional physician and the pharmacist. During the last few days that I was in Samtse hospital I could meet two other doctors, one was the chief medical officer of the hospital, who is based in the OPD this week, and another general medical doctor who is posted in the ward this week. While the chief medical officer said he cannot leave the OPD till 3PM even though he is interested to participate in the discussion the other doctor in the ward refused to participate in the discussion as she said she is not feeling well on the first day I approached her two days ago. However I was told that there is another young medical doctor who was away but will be joining this morning. And fortunately I met this doctor during the morning rounds in the ward and I approached him and he was interested to participate both for the survey as well as for the focus group discussion scheduled for this afternoon. I gave him the information form. Similarly the pharmacist was away for the last two days and had joined this morning and as I approached him he was willing to participate too.

The discussion was conducted in the Administrative Officer’s room upon the suggestion of the Admin Officer as I was told that the conference hall next door is too big and with the noise of the fans, as it is so hot here, and the echo from the room the recording will be affected. A simple lunch was arranged for the participants as the discussion could happen only in the afternoon. There was a heavy downpour this afternoon and the admin’s block was just under the roof for which I am worried that it might have disturbed the recording. Otherwise there was no other disturbances or any interferences during the discussion.

All the participants were participative and there wasn’t anyone who dominated the discussion. In fact I had to point to each individual to speak if they wished. The discussion was satisfactory and fruitful. Everyone felt that palliative care is important and required in Bhutan.

Thank you
