# Supplementary material for: Healthcare professionals’ views on how palliative care should be delivered in Bhutan: A qualitative study
Source: PLOS Glob Public Health. 2022 Dec 12;2(12):e0000775. doi: 10.1371/journal.pgph.0000775 (PMC10021767; doi:10.1371/journal.pgph.0000775)
Supplement: S20 Data — (DOCX) [file pgph.0000775.s021.docx]

**Transcript of FGD with HCP Samtse Hospital on 7/6/2019 (1.30 PM)**

| Participant 1 | Doctor |
| --- | --- |
| Participant 2 | Physiotherapist |
| Participant 3 | Nurse 1 |
| Participant 4 | Nurse 2 |
| Participant 5 | Drungtsho |
| Participant 6 | Pharmacist |

**Good afternoon everyone. To start, can we discuss on what motivated you all to participate in this discussion? After having read the participant information form, what made you think or interested to participate in this research?**

Doctor:

First of all I am very thankful for inviting us for this session. Actually it is a very important project and aah…now as you have already mentioned that our country is approaching towards like aah..NCDs are rising so in future palliative care is a must. So I think it is a very right time for us to integrate what the western people are doing and at the same time as you mentioned that it is a socially, culturally appropriate. So it feels very important. As you mentioned, for me as a doctor, I have not worked in palliative care but as a general practitioner we do see lots of patients with end stage disease where there is no cure and all we can do is symptomatic treatment. So there I feel is very challenging for us, not only for us, we have to not only deal with the patients we have to think about the patient’s party (patient's family) also. So more than the patients the patients’ party are involved there and sometimes we also think about that when we are doing something they should be culturally and spiritually acceptable and because of that sometimes it is very difficult for us. We want to administer simple drugs, we have to seek advice and sometimes the patient party do not want us to give those drugs. So I think it is very challenging and as a doctor I also feel that we have critical role in treating the patients with prolonged disease where we are deciding about the end stage of the patient. I feel that it involves like physical, social and spiritual problems integrated in such situation and at the same time for me it is difficult to counsel the patients when such situation arises. For treatment is very easy but to counsel patient and let the patient party know what the actual reality is very difficult and aah… at the same time at certain period we have to remove the end stage support also and there it is very difficult to decide when to call off (wean off) the support and when the patient should die peacefully. And at the same time patients prefer to stay at home and do symptom management. So I feel it is very important for us to understand when the end stage [of the] disease is occuring and when to focus on symptom management rather than treating the disease. So I feel, for symptom management, it requires multi team approach to be involved there, and not only the doctor. Even the palliative care nurses, even the physiotherapists, the staff who is monitoring the administration of medicines. So if those people do not understand what is palliative care then it is not going to be successful. So I feel that as a doctor it is very difficult to manage end stage of the patients. So I feel with your project I might learn some important aspects and how to realise the western and the Bhutanese care. What other people does may not be suitable here in Bhutan so we have to consider our background and the integration must be suitable to our culture. So I feel I can learn something interesting and important aspects while I am treating palliative patients in the future. Thank you

**Thank you sir. Thats very encouraging.**

Physiotherapist:

Aah…The reason I am here is kind of selfish for my profession because aah… it is like in Bhutan from what I have seen and heard we physios are not involved that much in the general patient care. It is just the pain management and the few exercises. Even in post op and pre op we are not involved that much but we have been taught about all these things. So I think like being here and having discussion I can put forward our profession that we can be involved in such things and we can be helpful.

**So you see your role in palliative care. Thats great. Thank you.**

Physiotherapist:

Yes, and then I am also hoping to learn more from this discussion.

Nurse 1:

Thank you for inviting me in this discussion. Aah.. I was interested in this topic, palliative care, because we come every day across people who require palliative care. We have patient every day who come through but we do not know how to take care because we are not taught. I don’t have any idea but just the general whatever I learned I think eleven or twelve years back. With that I am carrying out. So I got interested because I felt that I may learn new things to take care of my patients. Thank you.

**Thank you.**

Nurse 2:

Just like what my friend has said I also work in the (in-patient) ward so I thought maybe we could gather some ideas because we are not sure who is going to need palliative care. Even it can be my family member. I think it is for better understanding and to help each other its better we learn about it.

**Thank you**

Drungtsho:

(*Translated for Dzongkha*) At the present situation when there is an emergency cases and severe diseases we the traditional practitioners and not involved at all because patients also choose to directly go to the allopathy side where there is fast treatment. But I felt that if I participate in this study then we can also understand our role in terms of diet and behaviour and other therapies that we have in Traditional Medicine that we may link with physios and all that may help patients improve their quality of life. Also by joining in this study I thought we can contribute whatever little knowledge we have in helping such patients and moreover with the outcome of the study we can work together in future.

**(Translated from Dzongkha) It is particularly when patients with advanced illness have no cure that they look for alternative therapies. When they know that the treatment they are going through is of no help to cure them any more. They will want to go to anyone who is going to help them, you know. And Traditional medicine is a part of our health care system and we do see so much role traditional medicine can play to help improve the quality of life of patients and families in such situations. So that’s why we made sure that traditional physicians are also included. Thank you Drungtsho**

Pharmacist:

My name is R. R. and I am the pharmacist…

Interviewer:

**R can you be little louder please. Thank you**

Pharmacist:

I am so happy to be here. When madam approached me and introduced about this project I was so happy. Actually I heard from some of my seniors in the national referral hospital who talked about palliative care in life threatening illnesses. We as pharmacists have a major role because if palliative care is introduced (in Bhutan). This gives a hope to the patients even if it is a life threatening illness or a chronic disease. I as a pharmacist I felt that I can do drug intervention and contribute so much because just now in our country the pharmacists are looked down so much. They just feel that this discipline is not that important. That our job is just *doba, nima, chiru* (morning, noon and night) but in this hospital the doctors have started encouraging me to come to ward for rounds and all. I am so happy and feel that I can contribute from what I have learned from my college. So thank you for inviting me.

**Why do you think that people think that way for pharmacist, that your job is just doing *doba, nima, chiru?***

Pharmacist:

In our country in the past the pharmacy technician were known as the compounders so because of that. Today even though the pharmacy technician has good knowledge and they perform much better than the compounders then.

**It is interesting. Thank you.**

**Now to move on let us discuss on your experiences treating and managing patients who are terminally ill? What challenges you face when dealing with these patients?**

Doctor:

As I already mentioned that those patients who are terminally ill we know that they don’t really have cure for their disease. So what they and their family members expect is firstly they don’t understand what disease it is, and they don’t know whether it is curable or not curable. The first challenge is this one. If the disease they have is not curable and to say this one point is very difficult for us because breaking a bad news is not easy for us. For me personally I feel that breaking a bad news is very challenging. I prefer for treating the patient rather than breaking a bad news. So we have to consider other situations. We have to know before breaking the bad news we have to look at the settings where to break the news, in front of all the patients? In our hospital settings there is no privacy there. So we have to break the news in front of other patients and there is no separate doctor’s room also. Actually in the western context there is a doctor’s room where we can call them, consult them and we break the news in privacy. So in our context we don’t have a good setting there. So we have to break the news in the public places where all other patient parties will hear it. This is not only like a bad experience for me. Even to the nearby patients it is disturbing for them. So for me one thing is breaking bad news is very challenging. And to make them understand that the disease is not curable is very difficult. The second thing is that once the patients have understood that they have this disease they say that they want to have a symptomatic relief. So once the patient have, for example, in the case of cancer patients where they have no cure at all so we have to focus on the symptomatic treatment. So even for symptomatic treatment we have to know the patient’s preferences. Some patients they don’t want to come to the hospitals, some patients’ pain is really unbearable where we have to admit them but they don’t want to get admitted also. They want to do some religious things as you already mentioned that Bhutan is culturally very religious and so they prefer to do the rituals and all. So we have to consider that also. Sometimes we can’t force them also. So it is very difficult for us whether to admit the patients or let them do what they prefer. And sometimes we have to be forceful also considering the benefits over the risks. And once they get admitted there will be multiple complaints. Usually those cancer patients they have multiple complaints. One thing is the pain. And it is also very difficult how to tackle the pain. So actually WHO say that ladder wise pain management but the setting like in Bhutan like here in Samtse we don’t have all the analgesics available. There is only handful of medicines available in our hand and with that we have to treat the patients. So the availability of the drugs is also one major issue for us to tackle the things. And one thing is in hospital like ours we don’t have palliative nurse. In other countries they have specific nurses who can take care of only palliative care. Those nurses are very specialized and know how to handle such patients. We lack such specialities. Our nurses are all general nurses and have not received any trainings and even me as doctor I have learned such things while I was a medical student and during internship I learned few but I have not got any training as such to deal with palliative care. And one thing is that as I mentioned that we have to think about the relatives and the patient party. So once the patients are admitted those people we have to think. We should not neglect them. We have to look at their social wellbeing and we have to counsel them. And we have to manage them also. Sometimes we neglect the patient party where we only focus on the patients and we forget the background, the main supporting role. So we have to be wide and open and kindly talk with the patients. And the last challenge for me is when to call off or end the support. So when to stop that treatment. It is very challenging for me. Sometimes patients living in comatose state and is very challenging when to take off the organ support and sometimes even while treating the patients some patients prefer to do religious things inside the hospital. They come to us and seek our advice. That also sometimes we have to be cautious. In a setting like ours all patients are mixed together. We don’t have a separate rooms. On one side there is a cancer patient and the other side there is a pneumonia patient and sometimes TB patient. Now we have TB separated so it is not an issue now. So we have to think about other patients also. So like I said it is very challenging we don’t know how to deal with it. I feel that if we have a specific palliative care system, specific palliative team, as a team it would be very beneficial for all the future terminally ill patients. So I feel we do need a specific palliative team.

**That's very encouraging Sir. You have highlighted that there are so many factors at the moment while dealing with patients who are terminally ill.**

**Pain is one of the main symptoms in a patient with a terminal illness. Actually it is not only the physical pain that the patient goes through. Patient will have psychological pain, social pain, emotional pain and spiritual pain which is termed as ‘total pain’ in palliative care. And we do know we have lots of challenges but if we do not treat this physical pain first then rest of the pain becomes very difficult to treat. So to treat this physical pain we need adequate drugs, right? And specially to treat the moderate to severe pain we do need opioids and other strong drugs.**

**Sir (pharmacist), what all analgesics do you have in Samtse Hospital?**

Pharmacist:

Currently regarding the analgesics we have lots of drugs available like NSAIDS and all the pain killers we have. Then opioids we have morphine and codeine.

**So you have morphine tablets?**

Pharmacist:

Tablets, injections (both). We have sustained release tablets.

**So when you have a patient in Samtse hospital who is on morphine umm… say for example, I don’t know whether it is prescribed that way but when a patient has severe pain patients are usually prescribed 4 hourly morphine. Are you in a position to supply morphine tablets to a patient who is on 4 hourly morphine for about two weeks and then he comes back to get it refilled?**

Pharmacist:

Yes.

**So you have adequate stock?**

Pharmacist:

Yes we have adequate stock.

**OK. thats very encouraging.**

**Any other different experiences? How about the sisters (nurses) here because you have mentioned you see such patients daily day in and day out right? I am sure at times you must have felt extremely helpless. So what are your experiences?**

Nurse 1:

Aah.. like the first thing is the doctors explain the prognosis to the patient party, not to the patient, but to the patient party but they (Patient party /family members) don’t understand. But they get scared to ask again to the doctor. So what they do is they can nag us (Nurses) only. So we feel that we have to be more supportive to them because doctors do not have so much time but we are there for them 24 hours. But sometimes we feel helpless because patient is in pain and we are giving him the pain killer but that may not be the physical pain. He must be having other like psychological pain and all and I think we need some extra knowledge to counsel

**And to communicate, right?**

Nurse 1:

Yes

**Communication is a very, very important topic in palliative care and umm… Why do you think our patients are scared to ask not only the doctors, but patients are scared to ask the nurses or ask any health professional in general I think. Why do you think they are scared?**

Nurse 1:

Umm… Like may be in our society there is so much of aah…

**Hierarchy?**

Nurse 1:

The hierarchy with the doctors. Like if I share one example, few days back there was a TB patient and the doctor had asked them to shift to the old TB ward. And they (patient and families) said ‘its OK we will go down’ but then when I asked them whether they were ready. 'If you are ready the vehicle has come and we are going to take you to the old TB ward’. They (the patients) were like so aggressive with me. So the same thing happens, the hierarchy.

**So how did you deal with the situation?**

Nurse 1:

Then I told, I just cooled down, before I used to get angry but now I think that stage has gone so I just cooled down and said ‘ok I will pass your message to the doctor and let’s see what is the response’. So I feel like in palliative care also it must be like that. The patient may not be able to approach us or maybe we are so ..*laughs*…In our attitude we may be having that … we may have shown that attitude to the patient. And another thing, maybe the doctors do not have time because our doctors have to see so many patients and they have very little time. So the patients may not feel comfortable to ask also.

Doctor:

To supplement on what sister has just mentioned that aah…what the patient wants to ask the doctors and nurses largely depends on individual and we cannot generalize. For me when I go there, when I look at the patient, when I see them, if I approach from above (meaning if he act big/bossy) definitely they are not going to ask me. So what I generally do is in the morning I don’t get time to talk to them. I need to sit with them and if all the patient parties are there it is again going to disturb the others and the ward round. So in the morning I just keep the patient and one patient attendant in the ward. I do a quick review and I finish up and I usually come in the evening around 6 PM. I just do the evening quick round and I take that time to talk to the patients, communicate with the patient party and I generally spend around 30 minutes each on those who require counselling and all. So I always come in the evening just to talk with them and just to clarify their doubts and usually I select the patients those who require the counselling. So morning I am very busy so usually I come in the evening so that I can talk to them freely and so that they can feel.. that is the only opportunity I have to talk with my patients and patient parties.

**So in the evening you come and focus on those who require your attention? I think that’s a great way of doing it.**

Doctor:

And actually today I have one patient down stairs. This guy has carcinoma of the pharynx and he has received more than three or four times of chemotherapy and he has metastasis to his eyes and to the ears and throat also. So now at the moment the patient cannot take anything orally. So what we are doing is that just to give quality of life and quality death since the patient do not know, he cannot ask or say he is having pain. So we know that he is having pain because before when he could talk and say he has pain and was very painful so we have just put him on morphine round the clock. We just keep it round the clock and then we have to keep on talking with him. Like I always keep encouraging the patient party that even if he (patient) cannot talk or he cannot hear we should keep on talking with him to say that you are near him so that from inner side he can feel it. Then actually what we do is sometimes patients have their last wishes also. So like madam mentioned that palliative care is to provide the quality of life to those patients who have no cure at all. So during that period their life should be very peaceful and at the same time as it was mentioned the death should be dignified. So I always tell the patient party to even if they cannot respond just keep on talking at least once in the morning. So let’s say the patient downstairs also is similar. We have started on IV paracetamol also just to relieve him. So I feel that the quality of life while they are alive and the dignity of death must be considered.

**(*Translated for Dzongkha*) Drungtsho, do have anything to say? Such patients may or may not have come to you but having heard of such scenario today when there are such patients what kind of help and support can you think of from the traditional side?**

Drungtsho:

Till now may be because my service is still quite young I have not encountered such situation and no such patients also came to me but I can see such challenges because firstly when we deal with such patients and families we need counselling skills, right, and communication skills as well. And it is not just the patients but also the patient parties who are left behind after the death of the patients, to them too, we need to help overcome. So we need to plan all these.

**Do you see your role in taking care of such patients and families? As a Drungtsho, in such situation, even if you cannot help with whatever drugs you have, do you see other aspects of care that you can provide like the spiritual care or is there any opportunity for traditional medicine to improve the quality of lives of these patients and families?**

Drungtsho:

May be due to lack of experience madam, I am not able to say anything right now. Although we did study from the theoretical aspect that we can also contribute in such contexts but until now I think I have not dealt with such cases, may be none of us have, even the seniors I think, and may be in future too we may not be involved because in emergency cases it usually requires high care where they are directly referred to the allopathic medicine. So that’s why serious patients do not come to us. So I cannot say anything concrete right now but I feel we can contribute because we did study in theory but just that we have not applied in practical. I can see that we can give certain advice on diet and behavioural changes, then on the ways of caring and plus traditional medicine is so much related to Buddhism

**So you do see an opportunity to help in such situation right? So if we have a system because palliative care, as doctor said, is not a one-man-show. Palliative care is a multidisciplinary or interdisciplinary approach. That’s why we are different disciplines here. We have a doctor, we have a physio, we have nurses, we have Drungtsho and we have pharmacist. And we can have a spiritual leader, a social worker, and we can have volunteers. So it is a team approach. And the beauty of palliative care team is that there is no hierarchy in the team you know. Because palliative care is patient-focussed. Many a times aah… you know in conventional medicine it is disease focussed, right, where the treatment objective is how to cure the disease but when we are in a palliative care team our aim is the patient and the family. So that’s where the care differs you know. If the patient for example, on that day if the patient has severe pain the physician’s role is the most important and he is the leader of the team. On the next day the patient has a severe bedsore ok. The pain is not there but the bedsore is the biggest concern the role to play is the nurse. The nurse's role is critical today and it is like that and there should be so much of regard to eachother in the team. One of the days the drug, may be the morphine has caused severe constipation the role is between the physician and the pharmacist on how to help. So it is like that. There are patients with spinal cord injuries, lots of paraplegia patients and the physios have such a big role there right? So likewise palliative care is patient-focussed, palliative care is family-focussed. If we do not include the families then we are not actually doing right you know umm… our objective doesn’t get fulfilled because patient needs family and if we are against family then the patient doesn’t get the care that he requires. So that’s where palliative care is patient-and family-focussed.**

**So you now understand what palliative care really is, what do you feel about the staffing? And the training and other aspects. Even infrastructure, what do you feel about these aspects?**

Nurse 2:

Palliative care in Bhutan is new I think

**It is very new**

Nurse 2:

It is very new so like as our doctor said we keep all our patients together (at the moment). Even now we are keeping together two cancer patients. They are among other patients. So because we have already explained the patient and the patient party that it is a terminal case and they have less number of days and that’s why patients parties will come and many of them will come together (visitors) and that is where other patients are also disturbed. I think we need a setting for those kind of patients a good accommodation, and also the team who can look after this kind of people who are receiving palliative care. Setting plus the team and I think like earlier madam also said about the traditional medicine, there is a hope like when the doctor explains the prognosis and we are done with our medicine but sometimes the patient party might suggest what about the traditional medicine. Many actually does that aah…not just terminal cases but even other cases when people feel like they turn or they are like cross referred and I think this happens.

Pharmacist:

Can I add?

**Yes please.**

Pharmacist:

Because like there are many patients like when the doctor says that there is no cure the patient will go to the Drungtshos and there is this pyramid scale where there are stories where patients became the victims where they are told that cancer patients get cured and even diabetes is cured

**So all those misleading information you mean?**

Pharmacist:

Yes, such misleading information and this is a serious concern because last time I have seen one patient who stopped the diabetic medication. Like for three months he stopped taking diabetes medication and all of a sudden the sugar level went so high and I asked why he didn’t take medication he said that he is taking the Orien’s medicine. It is a biggest issue and a biggest challenge. Such patients are becoming the victims of those schemes in Bhutan. Even I feel that now DRA (Drug Regulatory Authority) and all have started to come on board to address this issue.

**So there is a big role of pharmacist to correct those misunderstandings and misconceptions, right?**

**Having understood little bit about palliative care and the service to patents who are terminally ill, how do you all feel? Especially for our colleagues here, the nurses. Umm… do you see because as I mentioned I used to feel very helpless when I saw patients who were dying. For patients who has cure he/she gets referred, you know, they are done surgery and somehow whether they struggled or not being in other countries they come back ‘cured’. But for patients who are literally termed as ‘no cure’ with lots of complications like for a Ca cervix patient in a corner of the ward you know, how do you feel about it?**

Nurse 1:

I think we have a bigger role to play to make them more comfortable like make them feel that they are also equally important like other patients.

**We have an opportunity, right?**

**How about you, our physio sir? From here onwards how do you see the future as a very young professional in the department?**

Physiotherapist:

Yes la, I definitely see the role of physiotherapy in such patient care. One thing is like there is very less information about such system. Like our friend here said about the Orien’s scheme. It comes from all these like when someone says that it helps all this wrong information spreads.

**They are so vulnerable, right? They don’t know where to go and they land up going to anyone who, at times may give them false hopes.**

Physiotherapist:

Yes, they have to be given the right information and aah.. they have to know that even after they get terminal illness they can live their lives normally and they can contribute to the society and hlp their family. It is just that they don’t have enough information about all this. So if there is such a system and then like if that system is successful the I think in the same way as the false information are fed the good information will also be fed to the people and then they will stop aah… and come back on track. They (patients) are often being misled. So if there is a good system which can successfully help such patients then more patients will opt for this care system.

In 2016, I and one of my colleagues went to Kerala where there is a very good palliative care centre there in Trivandrum and we attended a six-weeks training there. When we came back we made some sensitization presentations in Thimphu involving the Ministry of Health, the University, the JDWNRH, and the Bhutan Cancer Society where the present Lyonpo (Minister), who is the founder, was the Executive Director. So you know everybody thought this is important and thereafter few nurses from JDW were sent to attend the same training and today something is actually happening in Thimphu. They have formed a home palliative care team. You know stakeholders, even the Royal Family have identified how important palliative care is. There are support coming in and there are people who are interested. One of my concerns is we do need doctors trained in palliative care.

As a doctor, because many of the doctors at this time, in this modern era, look at treating the disease, right? Treating the disease, curing the patient and I did come across young doctors who said (Translated form Dzongkha) ‘oh those illnesses which cannot be cured is kind of boring’ you know, and they want that acute management, that instant treatment. I want to hear from you do you see potential doctors who would be interested in palliative care? What is your opinion sir?

Doctor:

Ya, as you mentioned that palliative system in Bhutan is new at the moment and I believe that there is no such word as palliative for us at the moment. So what we generally do is some patients are kept in medical ward like those who have stroke and all. Some patients with cancer are kept in onco ward and aah… I feel that like you mentioned there should be doctors who should be specialised in palliative care. You are asking me whether there is going to be doctors who are interested to join palliative care. I personally feel that it will depend upon the personal perspectives, their interest and what you have mentioned about the young doctors may not be true with everyone. So it may not be true also like I said there are lots of sub specialities to choose and out of that it depends on the individual preferences, individual choice. I don’t think that young doctors don’t prefer to be in palliative care. I feel that there are lots of interested young doctors who want to be in palliative care, want to serve in palliative care and provide aah… what you call…the terminal treatment. And I can think of some of my friends who would want to be palliative care physician also. So I feel that there won’t be any problem in future.

**Thats very encouraging. Thank you Sir**

**Aah… So I am understanding in Samtse Hospital there is not much issue with opioids or any other analgesics whenever there is a patient, right? Now you might be catering to the BHUs in the district, through the pharmacy. You must be the only pharmacist in the district, right? A patient, say for example, the patient is admitted in the Samtse Hospital with advanced cancer and getting discharged and goes back to the farthest BHU, I don’t know the farthest BHU from Samtse, and the patient is on morphine. How can you assure that this patient is continuously on morphine so that he is not in pain?**

Pharmacist:

For this we have a system by Form II and Form III. So those drugs which are not available in the BHUs they can indent from us. There is a form to fill where they need to produce prescription, get the form filled and take the medicine for three months. So it is the same thing with our GDMOs (General District Medical Officers), our doctors, from Thimphu they prescribe the drugs, they refill from there, collect from there. So it is same here too for those drugs which are not available in the BHUs. This things are already sensitized to the HAs and in some BHUs there are also pharmacy technicians. Just that the staff in the BHUs they need to be active and alert.

**I see. So there is a system already in place to ensure patients in the village have access to medicines, right? That's very good. Thank you**

**Umm…. Awareness… now can we discuss about education and trainings for the health care workers on palliative care?**

Nurse 2:

Umm… if we look into our setting, Samtse, we have shortage of staff as well and our area aah… I think we can first look into assessing who needs palliative care. We can like talk to them, their families and relatives and then we can explain to them about such things, introduce them to palliative care. We can tell them that there are this kind of system that can help the patient.

**Initially when palliative care started, it was mainly identified for cancer patients. But today palliative care applies to all patients including those with heart failure, lung failure, liver failure, we know how much ALD cases we have in our country, right?**

Doctor:

Spinal cord Injuries

**Spinal cord Injuries, HIV/ AIDS, and so on and so forth. And even for very old and frail elderly person**

Doctor:

CKD (chronic kidney disease), Dementias

**Yes, CKD and dementias and you know the list is so long and also palliative care is not only for patients who are at the terminal phase. Palliative care can be started at a time when a person is diagnosed with advanced illness. For example, say I have been sick for months and I finally get a biopsy and today I am given my biopsy result saying ‘I am sorry, you have cancer’. Patients and families are often devastated when they know the diagnosis,right? They go through so much physically, psychologically emotionally and spiritually. So along with the treatment therapies like chemotherapy, radiation, and other things, the other aspects of psychological, emotional, fear, all these can be taken care. So palliative care can also go hand-in-hand with the therapeutic and treatment aspect. This is very important to understand.**

**So basically I am understanding from you all that yes, palliative care is required, palliative care is needed,right? And it is important that we all learn about it, we get trainings, we have a separate infrastructure or at least a basic setting for it and then the drugs are in place, am I right? and aah… we all see the role of traditional physician as well. So it is quite encouraging that we have that potential to introduce palliative care.**

**Is there anything else to discuss tha we haven't done so far?**

*Nothing.*..

**Now you all are aware that this project is mainly to develop a palliative care model for Bhutan which is unique to our culture, our social context and our spirituality. As a last question what is your advice or suggestions for me? Because you are the ground reality, you see patients every day. So what is your advice to me for the project umm… so that those are taken into consideration and, you know, help materialise this project?**

Doctor:

So like you have mentioned earlier it is a multi-team approach, everyone should be involved, so I feel that advocacy should be done to make people aware and one thing as you mentioned awareness is very important and second thing is like aah… to bring about such change in a country like Bhutan, I think the parliamentarians should be involved to get their own point of view and as Bhutanese, we are a religious country, I feel that some religious bodies, like a monk and all those *Lamas* (religious leaders) should be also involved here. And those social NGOs, social service workers, all should be involved I feel. And at the same time I would like to word that palliative care in the west may not be applicable here in Bhutan. So I feel you should do some research like the package of palliative care that can be more suitable to the setting of our country keeping in mind our cultural background, our religious background, our society and our system also. So I feel like you have already mentioned that most of it is already comprehensive so if you can keep this things in mind I think palliative care will be successful and Bhutan will be having multi palliative specialised team soon.

Pharmacist:

If you happen to start in future this palliative care I want to suggest that because madam just said that palliative care can be started at the time of diagnosis but as sir just said creating awareness, like for example, someone comes with pre-hypertension and he is started on antihypertensive and educate him to continue the treatment. And if he stops he will have complications. So creating awareness is the most important.

**Thank you. Anything else? Drungtsho, do you have anything to say? Your opinion? Because here in this team almost everyone is supportive and complimentary to each other because when there is a patient the doctor refers to the physio, nurses will take care, pharmacist is anyway involved, you know. The main reason why we also involved Drungtsho is because in the Bhutanese Health system modern medicine and the traditional medicine are both under the same ministry, right? But till now the system is that if the patient wishes to see the traditional physician he goes there and those who wish to see the doctor does so. But when it comes to palliative care if these two specialties comes together it is felt that these patients and families would benefit even more. So today Drungtsho, you have understood about what is palliative care. Drungtshos in other hospitals have told me that in the Traditional Hospital in Thimphu they have started with all these healing therapies. So Drungtsho do you have any suggestions to me today?**

Drungtsho:

First I want to clarify this doubt, people often think that traditional medicine is only to do with Buddhism and so people who follow other religions like Christians feel it is not for them and they do not avail the service. Traditional medicine is nothing to do with Buddhism but it is so much to deal with our minds and so it has so much of relation with the spirituality of the person where we focus on the mind of the person irrespective of any religious background. So our main focus is not just the physical therapy but even the internal healing and that’s why those therapies in the Traditional Hospital like the Yoga, massaging, and meditation will all be effective in palliative care I feel.

**Thank you Drungtsho.**

**Anything else?**

Physiotherapist:

I also think, as doctor has mentioned, that we have to involve more people and not just the hospital setting. Like if there is a palliative care system there should be a proper infrastructure where people can go and counsellors are there. Also create job opportunities, like I have few friends who have done aah…their Bachelors in Psychology and now they are here in teaching in schools. So we can also create job opportunities for such relevant people and that can help both the country as well as the patients and so if more people are involved then I think it can be better.

**Thank you everyone. So this project is trying to develop a suitable model of palliative care based on a public health approach. Public health model is recommended by the WHO where the main components are; first look into the policies because if you are to integrate palliative care into the health system then you need to look at the policy where you may need to review and revise the policies in order to accommodate palliative care into the existing health system; and then the other thing is aah… train health care fraternity, create awareness among the public and every one; and the other thing is making drugs, palliative care essential drugs available and accessible, and the last component is to make palliative care service available at all levels of healthcare like from the referral hospital down till the BHUs. And to frame it socially, culturally and spiritually applicable to our context. As doctor mentioned we cannot just borrow, there are very good palliative care models in other countries like Germany, UK, Australia and the US, even in India but then we just cannot borrow that here. That’s why I am doing this needs assessment to look at our context, our aspect, to identify what are our needs based on those internationally defined WHO components. So this is Phase I. Next year in Phase II we will be involving the relevant stakeholders like policy makers, the parliamentarians, the drug regulators, the drug controllers, you know like DRA, BNCA, and then the Cancer Society, Bhutan Kidney Foundation, and then the clinicians like the nurses and doctors, patient representatives and family representatives, spiritual leaders from the Central Monastic Body, to advise on a suitable framework for palliative care in Bhutan. So from listening to you, from your opinion I feel what we have proposed is in line with what you are suggesting and ahh… hopefully if everything goes well then we will, you know, our objective is to bring in palliative care service to Bhutan.**

**Is there anything else, any final comment?**

**Nothing, right?**

**So thank you very much for your invaluable information which is going to really, really benefit the project. Thank you everyone.**
