# Supplementary material for: Healthcare professionals’ views on how palliative care should be delivered in Bhutan: A qualitative study
Source: PLOS Glob Public Health. 2022 Dec 12;2(12):e0000775. doi: 10.1371/journal.pgph.0000775 (PMC10021767; doi:10.1371/journal.pgph.0000775)
Supplement: S21 Data — (DOCX) [file pgph.0000775.s022.docx]

Field note of the interview with HCP in Sinchula BHU

Date 12. 6 2019

Sinchula BHU is managed by a couple who are both Health Assistants (HAs). With the help of the DHO (District Health Officer) who gave me the couple’s phone number, yesterday I called the husband who was the in-charge of the BHU to inform that I will be coming for data collection and explained about the research, its aims and objectives. He has heard about it from the DHO whom I had informed prior to my fieldwork in the first week of May. Since the husband had to attend another activity in the community, they decided that the wife will participate in the study. As she read the participant information form she agreed to participate both for the survey and the interview. However, she wanted to participate in the interview only if she can use the local language and not in English as she said that her English is not good and that she is not comfortable to do the interview in English. She had been in the service, initially as auxillary nurse midwife, a program which the Ministry of Health later merged with health assistant program, for more than 30 years. The interview was conducted at around 11.30 AM after clearing the OPD. It was conducted in the office of the BHU. The place was very silent and there was no disturbance or interference during the interview.

As a senior health worker, the participant had experiences of managing patients with advanced illnesses and patients at end of life. Although she has never heard of palliative care she seemed to be keen to learn about it and I could sense that commitment in her in taking care of such patients. She could identify various needs of those patients including physical, psychosocial and spiritual and could spell out the challenges she faced as she cared for those patients. She was very happy to know that such specialty exists and that this project is aimed at introducing palliative care into the Bhutanese Health care system.

I enjoyed doing the interview and it was very informative.

Thank you
