# Supplementary material for: Healthcare professionals’ views on how palliative care should be delivered in Bhutan: A qualitative study
Source: PLOS Glob Public Health. 2022 Dec 12;2(12):e0000775. doi: 10.1371/journal.pgph.0000775 (PMC10021767; doi:10.1371/journal.pgph.0000775)
Supplement: S22 Data — (DOCX) [file pgph.0000775.s023.docx]

**Transcript of Interview with HCP Sinchula BHU on 12. 6. 2019**

**Thank you Sister for willing to participate in this interview. To start with can please share about your experiences so far in taking care and managing patients with advanced illness where there was no prospect of cure, you know. Or patients with terminal illness or at end of life. Whether you have come across any such patients and if so what are some of your experiences of having taken care of such patients.**

There was one patient I remeber who was doubted as a case of Liver Cirrhosis but at the end it was cancer, you know.

**Was it Liver Cancer?**

Ya, Liver Cancer. So the patient was brought back to her home when she was in her last stage.

**Can you please tell me when was that?**

Aah… it was three years ago.

**OK.**

So when the patient was brought home what happened was that the family thought the disease was curable and at that time when we went to see the patient we provided nursing care to the patient but it was so difficult to convince the family members about the seriousness of the disease.

The man had two wives and it was his younger wife who was ill. And her son did not know that his mother is not going to survive and it was so difficult to convince this son because we were trying to tell him that she can be allowed to eat whatever she wants and let her wear what she wants and try to keep her as happy as possible but her son still wanted to restrict certain diet like she was not allowed to eat meat and when I was trying to explain to him he would get angry at me. When I told the family about letting her eat and wear whatever she wants and also slowly to also explain to her about the illness so that if she has any last wishes to fulfil, you know, because we knew that she is a terminal case and some of her family members also knew but in some family it is like that, you know, that not everyone has the same level of understanding.

**So some of them knew about the prognosis and others did not? And it is not always easy to make everyone understand, right?**

Ya, even if we try to explain and help them understand although they understand what is explained but within the family they had so much of conflicts. Her son would continue to say to the rest of the family that ‘my mom will get alright and none of you know about it’. And in this case I think one reason why the son was very confident was that the patient did not have pain and she could still talk and so he thought that she can still recover. And so he emphasised on diet restrictions and all. So that was what I witnessed typically.

**Interesting. And at that time how did you feel?**

To make everyone understand is very difficult I felt because one thing is they are uneducated and so do not understand about the nature of the illness. They have so much of belief and faith on the traditional rituals that they feel once all the rituals that are supposed to be performed for the sick is done then they have high hope that the patient will recover and that’s where I find it very difficult to explain to help them. Even to the educated people when we know that the disease is terminal and try to explain to the family that it would be better to keep the patient home rather than taking to the big hospitals which is only going to incur so much of expenditure, it is not easy to make them understand. They will want to try everything including the traditional healing practices where they have so much of faith and belief, you know, and they will even take the patient to a traditional healer like *Mata* (Hindu female traditional healer) across the border to India. So they will do everything possible to help the patient recover or cure the illness. So in such situation what we tell the family is that even if nothing has worked for the patient to let us know and we are always there to help them and the patient. And our aim is to relieve pain and further discomfort. Like they will be given pain killers from the hospital when they are discharged and we can give them those medications, we can give the required nursing care and try to make them understand that a terminal patient means that it is less likely that the patient will be cured despite the huge expenses they incur on treatment and rituals. But it is very difficult to convince because the community feels that certain local healing practices and rituals should help and they continue to perform all those. So that’s one problem we face in such situations.

**OK. So you feel that when there is no cure to the disease those traditional rituals are not helpful as well, is that how you feel?**

Yes, they will spend so much of money, you know?

**I see. So here in Sinchula BHU how often do you receive or how often do you have to take care of such patients, like patients at their end of life or patients with advanced illness with limited cure prospect?**

Actually not many of such patients comes to the BHU. Although they would have come here in the beginning when they were ill initially for the first time and that time if we doubt something serious or requiring further investigations we refer them immediately.

**So where do you refer from here?**

We refer to Gedu Hospital which is a district hospital and from there if required they are further referred to Thimphu national referral hospital. From Thimphu once they establish that the disease is advanced and/or terminal then the patients are referred back to Gedu Hospital, where they will be closer to home, for symptomatic treatment. And if patient and family wishes to be in BHU then we keep them here like we had one patient last year and even this year one month ago we had a patient with lung cancer. That patient stayed here till she passed away and her body was then taken to her village.

**So how often such patients die in the BHU**?

Very rarely, may be one or two in a year. It is actually not that patients do not come. Patients do come but they often come late, I mean they will come only after performing all the rituals at home and even across the border (Indian border) then only they will come to the BHU. Once they come to the BHU we do all the investigations that are possible here.

**What all investigations are possible here in the BHU?**

In the BHU we can only do physical assessment and nothing more. So when we doubt something serious we refer.

**Ok. So when you doubt that the patient could be having a serious illness, like for example cancer, you refer to Gedu and from Gedu the patient may be further referred to Thimphu. Do you think that there are more terminally ill patients from Sinchula community being treated elsewhere than what you see here in the BHU?**

Well, I don’t really think that there are many terminal patients in the community because we have system that once the patient is referred from the BHU here to Gedu hospital two of us (they are couple -Health Assistants in the BHU) here make sure that we follow up on every patient on what is the status of that patient and even if the patients are further referred to Thimphu from Gedu I call the patient’s family members to follow up what is happening. And all those updates we maintain in the records here. And we do that for all the patients whom we have referred from here.

**That is so good. So you told me that you had a patient with lung cancer who died here in the BHU, right? So was the patient here at end of life. So what were some of the needs of that patient?**

The main need for that patient was 24-hour oxygen supply and for that the family had bought themselves an oxygen concentrator for the patient. But we also had our stock of oxygen in the BHU for this patient to use when there was electricity failure because the oxygen concentrator functioned with electricity and here we have so much of problem with unstable electricity supply. Very often it keeps going off. So once we had this patient in the BHU I made sure that we have adequate oxygen cylinders as stand by because of the need for 24-hour oxygen supply. The other thing was to administer the medications and the patient only had morphine tablets to be administered. I even arranged to get morphine tablets for this patient from Gedu as we don’t have supply of morphine in the BHU. So I made sure that oxygen and morphine was always adequate for this patient. Patient often used to feel very hot, you know.

**How old was that patient?**

I think she was 75 or 76. And her son bought a table fan for her and he even bought a generator to ensure regular electricity supply.

**Wow...so he even bought a generator?**

Yes, poor guy he bought a generator also so that his mother can have continuous oxygen supply through the oxygen concentrator because they were not really satisfied to use the manual oxygen from the BHU when power failed and we also had to give her nebulization from time to time using electricity. And they also had many visitors coming. I even explained to those visitors about the status of the patient that she is in her last stage and ‘I am sure you all must be told by the doctors as well’ and they were all aware of it but they didn’t want to take the patient home.

**Why do you think they didn't want to take her home?**

Because I realised that the family didn’t get along well as half of the family members were Christians and the other half were Hindus.

**And what about the patient, was she a Christian or Hindu?**

The patient was Christian but half of her children were Christians and the half were Hindus. So in that situation when the patient was here in the BHU they could all come to visit her but if they took her home there was going to be so much of conflicts and they felt uncomfortable about it. They didn’t have a clear understanding as to how to deal in case she died at home. The patient party would insist that they would like to keep the patient in the BHU and all of them were I think comfortable keeping mom here in the BHU. When the patient eventually died here, you know, both the group performed their own rituals and we didn’t have any objection for that. We supported them according to their convenience.

**That’s interesting. So when you were taking care of this patient, who was at the end of life, what were some of the challenges that you faced? Did you face any difficulties in relation to the limited facilities here like you said you had to arrange for more oxygen for the patient? And even to bring in more morphine tablets? Did you face any problems?**

To get more oxygen and morphine was not so much a problem but having to witness such patient (at EOL) was very difficult for us. You know, she would wish to eat but she could not eat due to her breathing problem, you know, we tried liquids and she could not even swallow that. She could not even stand up for two minutes without oxygen. When the power failed we had to immediately connect to other manual oxygen or else she would go so breathless. We had to use a manual fan when the power failed. She was in such a pathetic condition that even for us to witness her situation was very difficult, you know. I used to feel like it is better not to be born than to suffer like that.

**So even as health worker you felt so helpless, right?**

Yes, I was so helpless. Even to help the family members understand the situation was not easy. When we tried to explain that the patient may go off any time and to be prepared, it was not even easy to say that the patient may die any time, you know. Not everyone had the same level of understanding. So it was challenging. I feel we need a good communication skills to convince them. Otherwise I felt there is so much of risk to us as health workers and to the family as well.

**So you really felt helpless taking care of this patient. Do you know anything about palliative care?**

I don’t know what is palliative care. I have not even heard of this term.

**Although you had been taking care of such patients and like in this recent case you knew that the patient was very ill, kept her in the BHU as the family aspired and gave so much of supportive care like giving continuous oxygen, managing pain and all those are supportive care you gave. And you did all those to help patient have a better quality of life for whatever number of days she lived, right. So such care is called palliative care. Palliative care is like even if there is no cure for the disease we can still take care of such patient to improve the quality of life of the patient and the family. When there is such patient even the family members go through lots of stress, it is very difficult for them and so they need help as well and palliative care can help not only the patient but also the family members as well. Like we can communicate with them, extend practical helps like if there is just one family member who is taking care of the patient all the time then palliative care can provide respite care. Palliative care team member can do shopping for them if required. Social worker who is in the team will take care of the social aspects like resolving conflicts, if possible and rendering social helps like if the patient and the family are economically deprived may be the social worker will help explore ways to help them financially. So palliative care can do all these to help the patient and families.**

**So as you understand what palliative care is how do you feel, how do you feel about this concept of palliative care?**

I really liked this concept of palliative care because in such situation it is not just one who suffers but many around that patient equally suffers and the suffering as you said is in different areas like may be financial or any other issue. I think it is so good because when patients and families cannot afford for treatment and management such help is so great, you know. So it is not just the medical care but care to the community where we can communicate and give health education, facilitate help.

**For the community here (at Sinchula), as a health worker if you initiate to facilitate the community to be engaged in helping such patients and families in their community in various ways like financial aspects, because in our country most of the people are poor, right? So for such patients, some may not have caregivers, some may not have money, so if you initiate to form a group in the community who can help such patients and families, do you think they will come forward or are the people in this community not very cooperative? How do you feel about it?**

People in this community are not very uncooperative. Of course there are always one or two who doesn’t agree but when the majority, like if 90% agrees then the rest 10% will definitely have to agree I feel. People in general here listens to our suggestions and comes forward. They are not very difficult in such things. I have been here for the last eleven years the patients and families here so far has always been cooperative. And one thing is when a patient is brought to the BHU they usually come in group. People from the village accompany the patient to the BHU. Once here if the patient needs to stay they work out who is going to stay with the patient and if the patient is sent to Gedu who all will accompany the patient they will decide all those. They discuss within themselves whether they have adequate money. There were few occasions when patients came here during emergencies like cut injuries and required referral and if they didn’t have enough money I would lend them and they pay me back later. If it was not a big amount I sometimes just tell them no need to pay me back.

**How amazing.**

And when the patients had to stay here in the BHU in the past there was no supply of ration for the in-patients in the BHU. There was even no kitchen or cooking facility. Later we constructed a patient kitchen. And because people will not know whether they will be kept in the BHU or not so they not bring along the ration so we used to supply them from our homes as a help. Then subsequently they will also arrange themselves. Later the BHU raised some funds by selling sugarcane and broom grown in the campus here and we bought rice cookers, curry cookers and water boilers. Today there is a kitchen where patient’s family can cook there and so there is no problem even if they need to stay in the BHU.

**That’s so wonderful because sometimes there can be patients like the one you had recently had who cannot be taken home due to several reasons, they may not have proper toilets at their homes, some will have such religious conflicts within the family, and so if they opt to stay in the BHU there is no problem at least from an infrastructure point of view, right?**

No, we don’t have that problem.

**And with kind and compassionate health workers it is amazing. So patient’s families can also stay, have cooking facilities and proper toilets and bathrooms?**

Yes, we have all those facilities.

**How about staffing here in this BHU? I understand that it is just two of you posted here and when you have such seriously ill patients kept in the BHU do you feel you would need another health worker posted here or do you feel that two of you can manage well?**

If there is going to be just one such patient any time we can easily manage by two of us. We won’t have any problem because here we don’t have so much of workload. Our OPD attendants is not very heavy because we have five or six ORCs (Out Reach Clinics) and many of the follow up cases come to the ORCs, like the NCD cases. Since we have five ORCs we cover most of the cases there and so our OPD caseload is not heavy.

**So if there is one patient for palliative care in the BHU two of you can easily manage, is that what you are saying?**

Yes, we can do that.

**Would you like to be trained for palliative care?**

Yes definitely.

**And in your BHU what all pain medicines do you have?**

We just have Brufen (Ibuprofen) and Paracetamol and besides those two we do not have anything else.

**So you do not have other stronger analgesics like morphine and all, right?**

No, we don’t.

**But then if patients come here with morphine prescribed from other higher centres you can arrange to have more morphine for the patients to continue pain management here in your BHU**

Yes, we have no problem to arrange an additional stock for a particular patient.

**So where do you get the additional stock for the patient. Is that from Gedu?**

Yes, from the medical store in Gedu hospital.

**Have you ever experienced where you were told that Gedu hospital has no stock of morphine and that they are not able to send for the patient who is in your BHU here?**

No, not so far.

**Thats so good.**

They don’t give us in bulk but they give us on weekly basis. Even in the past there would be patients like an asthma patient who would not want to go to a higher centre and would rather say that even if they are going to die let them die here but will stay here in Sinchula BHU. For such patients in the past we used to have only Salbutamol. Even for such patients we would discuss with the doctor in Gedu hospital and request to send us the necessary medications for the patients.

**And who brings the medications? How do you transfer the medications?**

Our caretaker goes to Gedu to collect the medication and sometimes my husband, the other Health Assistant, himself goes to get the medication.

**How good, how encouraging because it is said that patients with advanced illness usually have or moderate to severe pain and for such pain analgesics like Paracetamol and Brufen usually do not work. You don’t even have codeine phosphate or Tramadol, right ?**

No, we don’t have.

**And for such patients morphine is very effective it is said. Morphine is said to be very cheap and very effective for most of the moderate to severe pain. But in our part of the world morphine is utilised very little. From the literature that I have reviewed so far, the developed countries where palliative care is well developed consume 90-95% of morphine produced in the world. But in developing countries, like including India, China, Bangladesh, Bhutan, and other developing nations you know, in these countries there are actually more patients with advanced illnesses like advanced cancer and other NCDs but because palliative care is not well known in developing countries and so only 5-10% of the total morphine is consumed by the developing countries. How sad, right? So that’s why for patients in this part of the world their pain is very poorly managed. For that reason I am trying to understand the use of morphine here but I am so happy that in Bhutan the supply of morphine today is not so much an issue as in some of the developing countries. Even in the BHU even if there is no regular supply of morphine when there are patients who are already on morphine you can arrange to get a top up for their morphine stock. That’s very good.**

**Now, do you feel that BHUs should be supplied with morphine as well? Or do you feel that so long you get that additional top up for the patients it is ok? Or how do you feel about it?**

I do feel now that the BHUs should at least have a minimal stock of morphine and other strong analgesics. I think that would be good because we never know sometimes like if the patients comes to the BHU and has finished his morphine stock and by chance if there are road blocks due to heavy rainfall because climatic disasters can never be predicted, you know,

**I know**

So in such occasions we won’t really be able to help the patient who may have severe pain and we will be so helpless. We cannot tie him up to relieve his pain and when we have to witness such situation it is so hard. So I really feel that if BHUs also have morphine and other strong pain killers it will be very good.

**And if you have those drugs in stock you feel that you can give even better care to such patients, right?**

Yes, so much better care.

**Now when you came across such patients, besides the physical pain what are some other problems that they go through? Like they can easily complain of having a physical pain, and we can easily notive physical pain, right? They may already have prescription for pain management or even if they do not have one you can treat that physical pain to a large extent, right? Like the patient with lung cancer who died here in the BHU, what were some of her other problems, besides those family issues you mentioned?**

There can be lots of other problems. That poor lady was here but mentally she had so much of torture because she wanted to go home and she could not go on her own. She had her sons here with her, they were from her second husband. Her first husband had died and she and remarried and from her second husband there were three sons. She also had children from her first husband. And I think she must have felt that she was going to die soon and she was not happy that her children from her first husband did not come to see her. Of course she did not express that to anyone but very often when she was deteriorating she would call their names. So I used to feel that she calls their names because they are all in her thoughts and they did not come. She would ask whether her elder children have come or not.

And those can be family issues I think. She also wanted to go home because occasionally she would ask me ‘are you not going to discharge me?’ And I used to tell her that I will send you when you feel slightly better since I was never comfortable to tell her that she is very ill and that she might die any moment. I could never tell her that truth that she is dying in whatever nice and gentle way I tried. I felt so uncomfortable.

**Why was it very hard or difficult for you to tell her the truth?**

I felt that that will be a huge suffering for her which I could not imagine to witness.

**Plus she was never told this truth even in the higher centres, right?**

Ya, and even towards the end of life she actually did not know what her diagnosis was . She didn’t know she had lung cancer.

**So it was only her children who knew about it?**

Yes

**And the children also had not told their mom what she was suffering from or what her disease was.**

Ya, they also could not tell her and they decided that they will never tell her.

**So the patient never knew that she was actually going to die soon and must have continued hoping that she will get well one day.**

But I could feel that she also sensed that she will not get well. She used to say that ‘my past habit of smoking tobacco has cost my life. I think I am not going to make it.’

**I think in a way they do know that they are dying, right?**

**So as I tried to explain to you that this is a research project, my PhD project, and if it goes well the objective of the project is to develop a suitatble palliative care model for Bhutan. So as a very senior health worker in the field who has vast experiences and have come across patients who actually required palliative care, what would be some of your advice, or suggestions to me so that this project becomes helpful for health workers like you in providing services to such patients and families even in the BHUs and communities?**

I have found that your project is very good because when there is a patient with terminal illness it is not just the patient but also his/her family members and community who also would require help mentally, socially and spiritually. And we don’t have such approach of care in our BHUs at the moment. For an innocent villager he/she will not even know what actually cancer is. So it is very important to help them understand. So when you complete your studies and come back you should come to the BHUs to educate us whereby our approach to care for such patients and families will also improve and we can improve their quality of life, you know.

**Thank you and that’s going to be so fulfilling and satisfying, right?**

For the patients, their families and for us in the BHU it will be very satisfying if we provide palliative care even if the patient is ultimately going to die. I think we really need palliative care.

**Do you have anything else to say besides what we have already discussed? Like what you thought is important to mention but we have not discussed so far?**

I think I don’t think I have anything more to add.

**So the overall goal of my project is to develop a suitable palliative care model from a public health approach for Bhutan. So there are few things that needs to be focussed; first pont is we need to review and revise the current health policies to accommodate palliative care. So I have to look at the policy aspect, you know. Then after that is training the health workers because many health worker do not know what palliative care is. How would you know about palliative care because you were trained many years ago, even many of the recent medical doctors do not understand about palliative care? In this part of the world palliative care is a new concept so that’s why first we have to train the health workers then we also need to educate the general public, you know create awareness may be through the BBS (Bhutan Broadcasting Service) TV channels or social media. The public should also know that there is such care aspect even if there is no prospect of cure for the disease. Then the third component is making essential palliative care drugs available and accessible. Like for example, morphine and other strong pain killers and other drugs. It is not just the physical pain to manage even a constipation becomes such a discomfort and we need to relieve that. So it is called essential palliative care drugs and we need to ensure it is available and accessible. Those three components are very important. And on top of it palliative care has to be designed according to the cultural beliefs and values of each country. During chronic illness and death and dying one’s cultural beliefs becomes very important. So the policy, training and drugs components are same everywhere. It is a WHO recommended public health approach of palliative care. But the palliative care approach has to be culturally appropriate where the culture, beliefs and values of individual patient and family needs to be respected.**

Yes, That's very important to everyone.

**Therefore, this project involves public health approach model which is culturally, socially and spiritually applicable to Bhutan.**

That is an important aspect because in Bhutan today we have people with so many faiths and religious background and even within families people do not get along as I mentioned about a case in our BHU here. There was so much of conflict in that family, you know.

**And what must have been the mental state of the patient who was dying, right? How much pain the patient must have gone through.**

And I was trying to explain to both the group that she is everyone‘s mother and each one of you can help her in your own ways. No one has to blame the other. Let everyone come near her, talk to her. Sometime we get patients in the BHU who wants to perform local rituals and we let them do it. We don’t really restrict on it. But we tell them that they will also need to continue the given treatment from the BHU.

**Thats right. How nice.**

We have to do that otherwise it becomes difficult for us to handle the conflict.

**I know and to respect their beliefs and culture is also one of our responsibilities as a health worker, especially when they are dying, right?**

Sometimes we get patients who came to seek help but will not swallow even a single tablet that is given and if we plan to give any injections it is even worse. If something happens they will blame the medication.

**I know, I also came across such situation when I was working in the district hospital.**

And the community should also know about the availability of such care when they are struck with a life threatening or terminal illness.

**Anything else you want to share before we close down?**

I have nothing more to say. All I would say is you do well and come back to help many Bhutanese communities and for that best of luck to you from us.

**Thank you sister. Thank you very much. It means a lot to me and thank you so much for giving me your time.**
