# Supplementary material for: Healthcare professionals’ views on how palliative care should be delivered in Bhutan: A qualitative study
Source: PLOS Glob Public Health. 2022 Dec 12;2(12):e0000775. doi: 10.1371/journal.pgph.0000775 (PMC10021767; doi:10.1371/journal.pgph.0000775)
Supplement: S23 Data — (DOCX) [file pgph.0000775.s024.docx]

**Field note for FGD with HCP in Trashigang Hospital**

In Trashigang Hospital Focus Group Discussion was conducted with eight health care providers including a Chief Medical Officer, a general medical doctor, Drungtsho, two nurses from the general ward, a pharmacist, a physiotherapist, and a health assistant who works in the gyne OPD and follows up with patients who are diagnosed with Gynaecological cancers, HIV/AIDS and does pap smears. She was mainly included because she is involved in cancer patient follow-up and knows and understands what the patients are going through.

A simple working lunch was arranged for the participants because the discussion was scheduled in the afternoon and after a busy morning both in the ward and OPD the participants looked forward to participate in the discussion also because they were told about the lunch that will be provided.

The FGD was conducted in the hospital’s conference hall where a round table sitting arrangement was made and there was minimal disturbance. As we were about to start the discussion a nurse came running to call the doctors to attend to an emergency in the ward where a patient was having convulsion. Other participants agreed to wait and we could start about 30 -45 minutes later once the doctors joined after stabilising their patient.

I could observe that all the participants were keenly interested to participate in the discussion and there wasn’t anyone who really dominated the discussion although there were one or two who hesitated to talk more, particularly the nurses as usual. So from my experience in CRRH, I slightly changed the strategy and asked individually to give their opinion on each discussion topic which was a better approach.

At the end of the discussion almost everyone expressed what an important project I have taken up and that they look forward to having palliative care service introduced in Bhutan. The chief medical officer said that this was one of the most important and interesting research that he enjoyed to participate. “Other research that were conducted in the past did not make so much of sense” he said.

I understood that the participants found the discussion important, I learned a lot and over all the discussion was satisfactory.

Later in the evening, I was invited for a dinner by the paediatrician of the hospital whom I knew as a Paediatric Resident few years ago. In an informal discussion about my project she and another doctor, a gynaecologist, who also did not participate in the focus group said that nursing care in this hospital is very poor and that the nurses do not have attitude in patient care. They were directly saying that the graduates both from the faculty where I work and those who graduated from India are not interested in patient care. They do not provide any of the nursing care like bed making or any other personal hygiene and they are not even willing to do what doctors advise. Both of them expressed real frustration on the nurses’ attitude and I am very concerned but at the moment, as a researcher, I cannot do anything to improve or address the issue.
