# Supplementary material for: Healthcare professionals’ views on how palliative care should be delivered in Bhutan: A qualitative study
Source: PLOS Glob Public Health. 2022 Dec 12;2(12):e0000775. doi: 10.1371/journal.pgph.0000775 (PMC10021767; doi:10.1371/journal.pgph.0000775)
Supplement: S24 Data — (DOCX) [file pgph.0000775.s025.docx]

**Focus Group Discussion with Healthcare providers – Trashigang Hospital Date**

Date: 14/5/2019 Time: 2 PM

| Participant 1 | Chief Medical Officer |
| --- | --- |
| Participant 2 | General Doctor |
| Participant 3 | Physiotherapist |
| Participant 4 | Nurse 1 |
| Participant 5 | Nurse 2 |
| Participant 6 | Drungtsho |
| Participant 7 | Pharmacist |
| Participant 8 | Health Assistant |

**Good afternoon everyone. So just to start with aah…can we start by discussing about your experiences in taking care of patients with long term illnesses like advanced cancers, or patients with kidney failure or when you see patients with heart failure, lung failure, liver failure. What are your experiences?**

**Chief Medical Officer:**

Aah…thank you very much for the question. So aah.. I think to take care of those chronic patients basically I face three challenges. One being to treat biologically, another to treat psychologically and another to treat socially. So therefore, when it comes to biological aspect, so many of the hospitals in Bhutan particularly where I have worked is not well equipped for palliative care. Aah.. at the most that we have is morphine, of course that is quite OK for cancer patients, but then that is also in limited stock. So therefore we have to again restrict. And to procure that from somewhere else is a big challenge because that again needs money. We have to send some staff to mobilise that and that’s too much and TA/DA (Travel Allowance/ Daily Allowance) for this hospital is too limited. So that way. And on top of that also just other than NSAIDS like Ibuprofen and paracetamol we are not equipped with most of other analgesics. So therefore, in terms of biological management, from the pain perspective, very limited.

And also from other perspectives aah…most of our patients aah..aah..when they are chronically ill or when we first suspect to have a diagnosis of cancer, underlying cancers so we have to arrange referrals. And our referral centres are very far. Mongar is supposed to be the nearest and yet that hospital is also not that equipped. So therefore it is a big challenge. Aah….so coming to again biological aspect by the time aah..a patient is really diagnosed and, to be very frank enough, diagnosis is a big challenge still in Bhutan perhaps in some parts of the world as well. So therefore, there seems to be a delay in diagnosis of cancers and by the time patients do present it is already advanced most of the time. So therefore, there is a big challenge. And therefore patients invariably lands with palliative care forgetting about the radical therapy aah..because of patient’s condition.

Aah.. when it comes to psycho-social aspect aah.. this part of the country people are from quite faraway places and they are not facilitated with their personal vehicles, cars. So therefore very difficult. They have to make long journeys to the hospital and that too with big expenses. And even if they are equipped with money it is not easy for them to find a vehicle. Taxi charges are very high and that way they come from all the way from long (faraway) places and by the time the reach here and we also, because of all those biological limitations of therapeutic aspects for palliative care again we are quite dubious that OK we want to try help you but then we are helpless from social point of view and so for those kind of cases sometimes through our administration we do arrange certain amount so that if we can help but then that too is also quite limited. And so therefore it is a BIG challenge. So always a big challenge and we know the kind of challenges they do face. Because the moment they start from their place it is a challenge and when they reach here is a challenge because it (Hospital) is a strange environment, everything is strange. And most of the time our people are quite afraid that ok if I ask a question to that particular fellow would he shout at me and most likely that might happen as well. So very distressing sometimes. So therefore there is a big challenge psychologically as well as socially. So therefore, I think those are the biggest challenges and I have been, to be very frank enough, have been trying a lot myself to bring down those three main streams that I did mentioned right in the beginning. But however, the demography change is impossible for me and our aim even is to keep them longer but we cannot do much. Since palliative care doesn’t just boils down to curative but it is rather a holistic view and takes care of mental, psychological, spiritual, their background, and their views, whatever. So it is a very complete treatment. So therefore, with limited facility we will try and personally I am geared up to advising my staff to be quite compassionate and passionate and not to shout at the patients. That is my, actually more or less my regular advise given to my staff out here. To try to be decent and try to first listen to their problems. That is the only way that we can treat the patients quite comfortably. Or else we may miss so many things. So I think those are the biggest challenges. So as of now as per my experience for the last four years working in Bhutan and perhaps I may be too inexperienced to go ahead

Thank you Sir.

**General Doctor:**

Aah… to say something about that thing, palliative care, usually those patients who are terminally ill they are very much dependent to hospitals and during the course of their disease, at terminal point of their life usually most of the patients are aah.. much concerned spiritually. Since people are much embedded with the thoughts of spirituality at the terminal stage aah.. at terminal point they try to go home to make themselves spiritually fine, to make some, to have some, I mean like *rimdos* and *pujas* (rituals) they do because they think that if the hospital is not that or isn’t helping much because they are not aware of their disease. Sometimes because socially the family members doesn’t want the patient to know about her or his own condition, their prognosis and they keep on hiding. And then patients are actually frustrated as they thought that their disease is actually curable but they are actually not curable and they are just for palliation. So the most challenges recently we had a chronic pulmonary obstruction patient. He had infective exacerbation and he was here and I think it was HAI, Hospital Acquired Infection, and we had been giving antibiotics to just control that infective exacerbation. However he convinced me that we will go home do some puja and come back but however I heard that he succumbed to that illness. So what I am trying to say is that like other hospital, like the national referral hospital, if every hospital could afford or if our policy makers could afford a spiritual leader in every hospital so that our patients they don’t have to go home unnecessarily because they most of the time they go on discharge on request. They use to convince is that we are, it doesn’t mean that they are not grateful to hospital but so we have that thing call *menchey-rimdo* (medicine –rituals) so usually they want that spiritual part done .

**How did you feel when you heard that this patient died at home? After you heard that this patient died after performing the rituals at home how did you feel as a treating physician?**

**General doctor:**

Aah… I regretted that if the patient had not gone home, he was actually oxygen dependent, so he could have survived for few more days if he was here and if we had spiritual leader here whereby he could do some that spiritual thing here nearby hospital only. But he had to go all the way to Yonphula which is so bad for COPD (because of being a cold place)

**As a physiotherapist, what is your experience dealing with patients with life -limiting illness or chronic illness where there is no cure prospect ?**

**Physiotherapist:**

Aah..I would like to speak rather on the not exactly life threatening disease which normally physio doesn’t deal with it so we physios mostly deal with patients who are mostly the chronic conditions like spinal cord injuries. So in spinal cord injuries what happens is like aah.. we face the challenges somewhere at four to ten years after the injury. Say like from first year to say like five years patients still have a hope that yes, that they might regain the function, that they might start walking. Then aah.. they listen and does whatever we ask them to do or whatever we prescribe and they are always regular to the therapy sessions and all with the hope that they will regain the motor functions and all. But as the time goes there will be peak time that they think that yes, this is not going to come back. So this is a time that aah..somewhere between five to ten years that, it takes around five years to take that reality and that’s the time that patients attempts to take their own life. So this is what I have learnt during the short period of my time as a physio during the last 15 – 16 years. Then after crossing that tenth year of their paraplegic life then they start gaining that confidence in themselves that even without the limbs we can live with it. So they realise that limbs are not all that is required to live their lives. These are some of the realities that we as a rehabilitation team face. And during the peak years we have to be very careful with their feelings and all. We lost most of the SCI patients during that period about five years after the accident and this is the very aah.. like the cream age or the time for the health care team to take care of those patients. So if palliative care is coming I think our physio staff can be trained on it where we can deal with those patients and where we can really convince them about the conditions and all and this is what we rehabilitation team face.

**So you see that palliative care can play a significant role in physiotherapy, right?**

**Physiotherapist**:

Yes, yes, especially for SCI pts time plays a significant role la. Because in the early phase even the educated lot they do have a hope that 'yes my spinal cord is going to come back.' Aah… I have one patient who has complete T10, T11 fracture, a complete injury and still has a hope and just now he is in that 'hope' stage. May be, I don’t know, may be after five years, now it has been three years and may be after two years he will start deteriorating his hope. So these are some of our challenges.

**So may be after two years he may give up hope and thats when he may consider taking away his own life, right? and that’s where he needs help, counselling and other necessary support, isn't it? Have you had patients who took it positively as well?**

**Physiotherapist**:

Basically few years after crossing the first five years they don’t, for at least five to ten years they don’t take it positively. Normally most of the spinal cord injury patients they take it negatively only. And there are some patients who had SCI for last 18 to 19 years and still living with negative emotions. There is someone holding a high post in the civil service and he is still having that negative outlook. So these are some of the challenges that we are not able to convince the patients.

**Real challenges indeed.**

**Now we have three nurses here. As nurses, who are 24 hours with the patients, you see suffering in patients and you see everything in patients and families, what is your experiences?**

**Nurse 1:**

(Translated from Dzongkha) As nurses we are together with patients for 24 hours but whatever happens to the patients we have to inform to the doctors. When it comes to like personal hygiene and other care it is in our hands but what is difficult for us is that for us to keep the patient clean and tidy at times the family members say that 'since the patient is anyway going to die you (nurses) don’t have to do anything to the patient'. And that’s the problem we face. Some time for us peaceful death of a patient is our priority and as we work to assure peaceful death to our patients at times I feel if we can create the same awareness to the family members. Till now sometimes some patients having big wounds are taken home by the families and they never come back. We wish that they can be cared better in the hospital with wound dressing and pain management where they can have peaceful deaths. From our side we do what we can but the social issues like the financial burden is huge in Trashigang and it may be same elsewhere but for some patients who are very old with no one to take care we witness the old couples quarrelling and we try from our side but when we don’t have their cooperation we cannot do much to help them. However, later if there is a financial help for those who do not have any one to take care, if the government could afford to do this, I think it will really be good.

**So you mean that when the patients are terminally ill and when you approach to provide nursing care the family members do not let you do anything, is that what you mean?**

**Nurse 1:**

Yes.

**Can you please explain further on what type of nursing care or on what nursing approach do they refuse your help?**

**Nurse 1:**

For example once the prognosis of the patient is explained to the family members by the doctors then whenever we do something to the patient they will ask 'what are you doing' as if they are angry at us for doing something and they will say please don’t touch. They say that the patient is anyway going to die, why you should touch. They seem to believe from a spiritual perspective that when a patient is terminally very ill that the patient should not be touched and when they say that we also cannot do much no la. So we also don’t do anything and leave them alone.

**What do you say (another nurse)? How do you feel about it?**

**Nurse 2:**

Aah.. most probably mental weakness is very important to address while providing nursing care because the terminally ill patients usually stay in the hospital for long duration and so they get exhausted by seeing medicines, doctors and nurses. So they feel somehow weak, aah… mentally weak I mean. So maybe we can help them through having some outdoor parks or some recreation facilities.

**OK. So here in this hospital for people here, as per your experience what could be some of the best recreational facilities that would benefit them, that would be suitable for this context?**

**Nurse 2:**

May be they can go for some walk around the hospital, or maybe they can go around *mani dungkhor* (Prayer wheel).

**Interviewer:**

**Which is there already, you have one right? So that might help them to heal psychologically, right?**

**Chief Medical Officer:**

I think other perspective or the challenge which I forgot to mention earlier is probably our patient-staff ratio is not working. So because generally when we talk about palliation or palliative therapy so we are talking about patient who is chronically ill, bed bound, bed ridden and terminally ill and who needs constant vigilation and monitoring, aah… constant concentration I mean. And since we have shortage of staff including doctors and staff nurses out here in Trashigang hospital. So just to focus on that particular person aah…biologically, psychologically, socially is as of now is quite a challenge because that demands lot of time. So therefore sometimes may be from that perspective we might be becoming quite impatient and then we are trying to just focus on the biological aspect of the treatment forgetting the psycho social aspect. So therefore if we had adequate number of staff members and particularly if someone is trained in palliation, palliative therapy so really I do feel that those patients will be benefitted, like a palliative nurse or someone like that. She or he will focus only on those patients and in particular they would be trained by the time and on top of that to be very frank we doctors, nurses out here, general staff in Bhutan altogether in general is not trained for palliation and as I said palliation is just OK pain killer, so that’s the only concept. So probably I think now we have to gear up. So that’s the challenge. No trained palliative staff and more patients but less staff, a challenge for palliation.

**Ok so as we started discussing about experiences we also discussed about challenges. Challenges you mentioned were related to staff – inadequate staff, lack of training, inadequate knowledge on palliative care. Any other challenges la?**

**Besides inadequate staff, inadequate knowledge and lack of trained staff are there any other challenges? What are some of the social challenges? You did mention about patients being poor, so financial aspects, right? Any other challenges?**

No answer…

**Of course may be illiteracy as well in the community because illiteracy in patients also plays so much of role in diseases and its management and its outcome, right?**

**Health Assistant:**

(Translated from Sharchop) There is this one thing about patient attendant. Those patients who do not have attendants or not having right attendant. I faced a situation where there was a woman who has not even done pap smear and she was diagnosed with cervical cancer and there was so much of foul smell and her attendant was her son and she did not have any one else. And there is so much of awkwardness between the son and the mother when it comes to reproductive health. So this was one of the problems I witnessed because it was so much uncomfortable for both the son as well as the mother and she did not have anyone else to take care of her.

**OK and this is very important in our context, right? because in the west family member plays a least role because there they have a system where children don’t necessarily have to look after their parents and they have proper system of care organizations like, Aged care facilities, nursing homes and very advanced palliative care services. But in our context we are so dependent and our family members gives so much of support and care care. In Kerala, family members plays a key role. They know how to do wound dressings, they know how to prepare normal saline. They are the key persons. Health workers facilitates but the care because they have trained the family members, family members take care of the patient. So I agree that when there is no family member or when there is not the right famly member, how much issues there can be and that’s where palliative care team can help identify community support system.**

**What is your inderstanding on palliative care? How many of you have leaerned about palliative care in your undergrad studies?**

**Nurse 1:**

(Translated from Sharchop) Actually when it comes to this I did learn little bit about palliative careduring my nursing training in India and when I came here although I haven’t really stopped applying the knowledge and I still do what I can but I have not been able to provide continuous support because this is a district hospital and to be honest there are two specialists and staff shortage is one reason I feel because when there is one delivery case then more than the chronic case delivery case becomes more important. Likewise if there is a paediatric distress case then should we focus on the chronic case or attend to the distress case? Although it is not that chronic case is not important but while prioritising the urgency and with shortage of staff, during night duty also we are just two staff and so we can’t really focus on the chronic case. However, it is not that we have stopped providing supportive care but because we are not able to provide adequate palliative care we remain unsatisfied. Actually we do receive chronic patients and we do attend to them but we are just not able to provide adequate care.

**So here too the challenge is due to inadequate staff and due to lack of training on palliative care, right? Had there been others who are also trained in palliative care then you will be able to prioritise accordingly, is that what you mean? Because as you prioritise for a distressed patient and as you prioritise for delivery patient the need of the dying patient can be prioritised, right? I just wanted to know what other factors are there which you made it very clear. Thank you for that.**

Umm…I think we will now focus on our pharmacist sir. Sir, palliative care is primarily pain management and so drugs like analgesics are needed. I think you must be aware of the WHO analgesic ladder.

*Pharmacist shakes his head indicating that he is not aware of WHO analgesic ladder*.

**That's OK. The WHO has dveloped an analgesic ladder to be used for pain management in palliative care.It is called the WHO analgesic ladder. So there are three steps in the ladder. Step one is for patients with mild pain then it is managed with step one which includes paracetamol, brufen, you know those basic analgesics, and other adjuvants whatever is needed for the patient. If that doesn’t work and if the patient has more than a mild pain, moderate pain, then you go to step two where you give mild opioids like aah..codeine phosphate, tramadol you know and then some adjuvants like anxiolytics and whatever is needed for the patient. But if the patient has severe pain then the patient must have narcotics, like morphine and other opioids. Now most of the time as doctor mentioned our patients are diagnosed very late. So when they are diagnosed they usually have moderate to severe pain and where umm…first step is hardly helpful and they need narcotics. Now as a pharmacist in the district hospital, my first question is do you have morphine supply in the district hospital?**

**Pharmacist:**

Yes, we have morphine supply. Aah..(translated from Dzongkha) here I will have to say directly that now the problem about morphine and analgesics is aah..mostly our patients will have already gone to other hospitals prior to getting treated in Trashigang hospital. That’s the first thing. Like aah.. like cancer patient some are coming to Trashigang, right, after being diagnosed in Thimphu hospital. The situation in Bhutan is difficult, very difficult. When we keep a stock it is sometimes difficult because if the drugs gets expired we have to give explanation on why it got expired. First thing the problem in Bhutan is that doctors in Thimphu or like in higher centre will diagnose like end stage cancer or likewise and they will have told the patient to collect medicine from Trashigang. So it is very difficult to provide medicines at that time. That’s one thing. So second thing is, second problem is right now we do not know the morbidity data. We don’t have that right now. And we don’t know, health staff or even the DHO doesn’t know that the patient is coming, right. Then the third problem is that the patient will demand from anywhere, any hospital. So that’s the problem. So how to solve these problems? How to solve palliative care problem? I feel firstly my opinion is we need data, data about patient, right. How many patients do we have? Even if the patient had been there for three to five years we do not have proper data (records). So where does the patient usually reside is important to know. Then the second thing is patient will travel to Thimphu where they will have a relative or to Gelephu if they have their relatives there and likewise in Mongar. Then they will migrate always. That’s the problem we face. Last year we had morphine, before last year we had morphine in stock and it got expired. That’s an example. So how can we procure? How to indent? How to compare? That’s the problem. There was no consumption in last few years and in the coming year we don’t indent but then we get into trouble in the coming year. So that’s the problem. So that’s why we need data and then we need to know the context like these many patients on analgesics and these many on narcotics. So if we have these the problems might decrease I feel. Thank You

**I think this is very important..**

**Pharmacist:**

In Bhutan if we do not have data we can’t do anything.

**Chief Medical Officer:**

Cannot estimate how much is needed

**Pharmacist:**

And last year like we had an opportunity to indent MDIs which are used for chronic patients like COPD. MDIs are actually indented only in the national referral and regional referral hospitals but last year we could do for our hospital as well. So we indented few thousands and finally we received letter from the Ministry of Health that it cost huge amount, in millions, and it got cancelled. So that’s the problem. So this time we got limited stock of MDIs.

**What is MDI?**

**Pharmacist:**

Metered Dose Inhalers. So these are some problems. So solutions may be we have to discuss with stakeholders like Ministry, policy makers, even like budgeting. Otherwise it is very difficult.

**At the moment, say for example a patient from Trashigang has gone to Thimphu and got diagnosed with an advanced cancer. Now the patient wants to come home. Patient is told that it is a terminal illness and so he/she wants to come home. Patient is in moderate to severe pain OK. Will it be possible to arrange opioids for this patient?**

**Pharmacist:**

Ya, we just have morphine tablet in this hospital. Usually we try to keep minimum stock for some patients. We don’t know how many patients will visit us. So we keep a minimum of 100 tablets. Just now we have a patient from Pam ( a nearby village) who usually takes Morphine SOS (*whenever necessary*).

**Let us come to Drungtsho now. Aah.. traditional medicine is integrated into our health system, right? When you have a chronic patient or a patient who is terminally ill, what is your approach? Aah.. does the patient come looking for Drungtsho or do you get referrals? (translated from sharchop) or when patient comes to Drungtsho do you refer terminally ill patients to the doctor or does the patient choose to go themselves or how is it?**

**Drungtsho:**

(Translated from Sharchop) At the moment it is as per the patient demand, I mean patients themselves come looking for us and sometimes doctors also send them to us for consultation. I will put it into five points for discussion. First one is the cross treatment between homeopathy and Ayurvedic, next is the diet, then patient attendant which was discussed earlier, and the last is patient demand. So firstly about the cross treatment is like first the patient goes for allopathy treatment, like a patient with high blood pressure gets treated with allopathic physician. As they get treated with allopathic medicine they will realise that the medicines have side effects like kidney failure or so on. Then they will discontinue that treatment and come to us. That is one problem because when patients are on allopathic treatment the drugs are strong because they are all chemicals and when they start traditional medicine which is not as strong as the allopathic drugs so it doesn’t help to treat the patient. Now other thing is about cross therapy. Some of the patients, when they wait for the results of their blood tests like RFT (renal function test) or LFT (liver function test) which are usually ready by 2 PM or so, they come to us and ask for therapies like *serkhap* (acupuncture) and that’s another problem. Now the next is regarding the diet

**Can I interrupt you Drungtsho, we might forget this later…Just wanted to clarify.. is it that if the patient has not got his/her LFT report you cannot give *serkhap*?**

**Drungtsho:**

What we believe is that if blood was just drawn from the patient then giving *serkhap* will not benefit the person because it is said that both these procedures cause pain and will have no benefit. Some patients what they do is they will first come to us, get *serkhap* and from there they will go to a doctor who will then advise for blood test which is again a problem. So if the treatment is mixed there is no benefit.

**Say for example, there a patient with heart failure who is on anti-failure drugs and also comes to you. Is it that the person cannot take both the treatment at the same time?**

**Drungtsho:**

Yes, we should not mix them because the efficacy will be compromised. We particularly focus on hypertension because hypertension patients tend to avail both the services at the same time and we discourage. Now the other thing is the diet. There are so many diet restrictions that is required when the patient is on traditional medicine.

**How about for those advanced cancer patients or those patients who do not have a cure for their disease? Do they still have to restrict their diet?**

**Drungtsho:**

Yes, they will have to. Then the other thing is regarding the patient’s attendant. Last year there was a problem. The patient was referred to us from Rangjung BHU. After assessing the patient I was getting ready to draw blood for some tests.

**So am I understanding that you also do blood tests in Traditional Medicine?**

**Drungtsho:**

Yes, we do various tests depending on the needs for patients. So what happened was as I was getting ready to draw the blood the patient just collapsed on the chair, I thought she had that phobia for needles, and the patient did not have an attendant and moreover both my staff were out of station and I was managing the unit alone that day. So then I immediately called for help from the doctor and other staff and then we transferred her to the examination bed and observed her where she recovered about two minutes later. So when there is no accompanying attendant with patient we face many problems.

**So you mean to say that patients should have their family members accompanying them when they come to hospital?**

**Drungtsho:**

Yes

**And I guess it is even more important for patients who are chronically ill with advanced illness, right?**

**Drungtsho:**

Yes. And the other thing is the patient demand. Patients do come to us asking for specific medicines. We had one *Tshampa* (a yogic practitioner) who comes to us and demands that specific medicine even before we assess him. *Other participants laugh*.. So we do face such problems. Then we also refer patients to the doctors when we find that their problem is indicative for surgeries and other medical procedures.

**Now, when there are patients with chronic illness at an advanced stage where they go through lots of physical pain alongwith psychological, social and spiritual pain and distress**

**Drungtsho:**

*Even before the question was completed….*For such patients we advise that treatment for them should involve both medical help as well as religious rituals that health and religion should go hand in hand. We do get patients who complain that they are not getting better with treatment from both allopathy as well as traditional and we ask them whether they also conducted some rituals and encourage them to do so since it helps in the psychological and spiritual distress. During our outreach clinic services, mostly our patients there are the *tshampas* who are on retreat meditating in the caves up in the mountains with complaints of chronic airway disease and chronic joint pains for the last few years. Most often they will send somebody else to get medicine for them and those family members or whoever comes will not even know the exact symptoms and the exact information about the patient’s problems. So these are some of the issues we face.

**Thats interesting. Aah..I think we have already covered in the discussions although I had sequential questions but we did cover in bits and pieces here and there. So we talked about experiences, we talked about the needs for the patients. Do you have anything more to add here? When there is no prospect of cure for the disease what are some of the pertinent needs in the patients and families, like we discussed about the financial needs, family members’ or attendants’ needs. Is there any other needs? What are some of the specific needs in patients and families that you are not able to address?**

**Chief Medical Officer:**

Aah… I think from Buddhist point of view towards the end of life they would really like to perform some spiritual rituals which I think most of the time is not permitted because of the risk of hazard with incense burning nearby oxygen cylinders and all those limited facilities in the hospital. Actually I think there should be aah..aah.. but as of now after I came here there was no such request but usually I do entertain them by ensuring that they are not burning incense or lamps nearby oxygen cylinder. I think we have to allow that because that is one of the needs towards the terminal phase. So they would request that really genuinely. And we have to also, if the space allows and if the surrounding is comfortable, I think why not allow them to do some *puja* (ritual) not just for the Buddhist but to anyone with any religious background. I think we need to entertain those things in the hospital but making sure that fire hazards and other risks are prevented. That way we can comfort the relatives and also the dying.

**And that would improve their quality of life, right? That’s where palliative care plays a role. So long they don’t create problems, so long they don’t burn incense nearby oxygen cylinders which will be a big issue then. That’s exactly what palliative care is, fulfilling what such patients and families wants and if that is going to improve their quality of life. And this gives me an understanding that we do need a separate unit for palliative care. In an ideal situation, patient has a bed and the family member who is supposed to be with him day in and day out may have just a stool for him to take rest, right? Because here our focus is just the patient but palliative care identifies that family member also needs rest. So in palliative care unit there can be a small bed for the family member who also needs rest. And that the patients can also practice their own beliefs and values whatever is possible in that small unit at that moment, of course not go on for days performing rituals, but yes anything specific like *motapni* (seeking spiritual opinion) or anything which will improve the quality of life of the patients and family members.**

**Drungtsho:**

In the Traditional Medicine we have rooms for practices like Yoga and meditation for patients who would like to avail such services.

**Do you have such facilities in the districts?**

**Drungtsho:**

At the moment we have not started in the districts yet. It is only in Thimphu in the Institute of Traditional Medicine. At the moment they are training the traditional physicians on those services.

**I see. I heard about it from the Drungtsho in Gelephu Hospital. He was saying that yoga and meditation can also help patients when there is no medical cure. That’s interesting, right?**

**Drungtsho:**

Yes, they can be helpful

**Now to the pharmacist sir, what do you say about the current narcotic regulations? As you mentioned about the challenges and the reality of having the drugs here where you mentioned about the not having proper data and other factors in patient management, what do you say about the current drug regulations? Do you think it needs to be reviewed and revised because more than anybody else I think you would know the regulations best in order to ensure the availability and accessibility of opioids for patients with life threatening and terminal illness. Do you think the current regulation is ok or do you wish that it is reviewed?**

**Pharmacist:**

Ummm… I of course do not have any idea about the narcotic regulation but aah.. aah…as per my knowledge the DRA (Drug Regulatory Authority) monitors the use of controlled drugs in health. When it comes to regulations, when a patient is prescribed by the doctor it means it is essential for the patient right and so they are not strict for those who genuinely need it. So I think there is no need to change any regulation at the moment concerning narcotic drugs and that’s my opinion. But when it comes to control we do need to monitor misuse because sometimes the health staff themselves are the source of abuse of such drugs. Abuse in the sense, even if they do not abuse themselves, at times our health workers do issue control drugs to their friends who do not have proper prescriptions by just maintaining the record in the register. So we do need to be strict in controlling misuse but for patients who are prescribed they are genuinely in need and they are issued as per the need.

**So the control is mainly to prevent misuse but it assures provision to patients who needs it, right? And that’s what the International Narcotic Control Board emphasizes as well.**

**Anyone has anything to add on this?**

*Nothing*...

**So now to summarize, aah… you are all aware that these information that I am gathering from you all is to inform a suitable palliative care framework for the country, right? If everything goes well, the objective of this research project is to integrate palliative care into the Bhutanese health care system. Besides what we have discussed today, do you have anything pertinent advise to me, any suggestion or comments that would help integrate PC into our health care system? Because you are the ground reality, you know the real issues or the real picture here and my study will be based on whatever information I get from the study sites. So besides what we have discussed so far do you have any advice or suggestion to me?**

**Chief medical officer:**

I think my opinion is that doing such a research is great and most likely your paper will get through and be accepted, *smiles*…. Much more than that the most important thing is that how to really make sure that our policy makers do accept that because policy makers themselves should be made aware of what is palliative care first and until then they will always say NO, they will deny, they will say we have enough nurses, we have enough doctors, physiotherapists, pharmacists and that we don’t need that as of now. Probably that will be the answer. But my suggestion to madam would be after this research if madam could take that extra initiative and responsibility to really somehow through health ministry or RCSC (Royal Civil Service Commission), through someway, get through them and let our policy makers know the importance of palliative care so they even might talk in the House (National Assembly House). So I think that will really become practical one fine day otherwise I don’t think…*laughs*…

**Las la.. I think that’s very important suggestion. I have noted your point Sir. Thank you very much Sir.**

**Physiotherapist:**

To supplement on what Sir (CMO) said aah.. like…I would like to wish you a very best of luck for the publication of your paper.

**Thank you very much Sir.**

**Physiotherapist:**

And with it as doctor has already mentioned about the importance of high level authorities so I will just share one example. We in the physiotherapy department have been working with many stakeholders to have a Disability Act of Bhutan and we are yet to get one and I think in coming summer I think they might discuss in the Parliament. So as doctor has rightly mentioned if we can have a Bill on Palliative Care in the country then it would really benefit because we have already discussed about the uprising of the NCDs so if you could have a Bill then they will discuss in the Parliament and the relevant stakeholders can really help and similarly if those people are involved then they will definitely help with the infrastructure and human resources development. So that is one thing you may consider on high level talk.

**Very important suggestion and a very good reminder Sir. Thank you. Anything else la?**

**Nurse 1:**

Translated from Dzongkha… For the work you have taken up we have all our support to you. And our aspiration is that palliative care gets integrated into our health system and once it gets integrated then we would like to avail the training opportunities on palliative care.

**Sure. Thank you very much.**

**Is there anything else that you wish to discuss that we didn’t do so far?**

Nothing….

**So I understand there is nothing left to discuss further. And once again I want to thank each one of you here for your participation. The information you shared today is invaluable for the project and thank you for giving me your time, for your patience and I know it is beyond 3 PM and I am very grateful to everyone for the participation.**

**Thank you very much!**
