# Supplementary material for: Healthcare professionals’ views on how palliative care should be delivered in Bhutan: A qualitative study
Source: PLOS Glob Public Health. 2022 Dec 12;2(12):e0000775. doi: 10.1371/journal.pgph.0000775 (PMC10021767; doi:10.1371/journal.pgph.0000775)
Supplement: S25 Data — (DOCX) [file pgph.0000775.s026.docx]

**Transcript FGD with HA Ura BHU Grade II**

Date: 25.5.2019

| Participant 1 | Health Assistant 1 |
| --- | --- |
| Participant 2 | Health Assistant 2 |

**So to start with… can you please share some of your experiences so far, not only in Ura BHU but elsewhere as well, your experiences on caring for with patients with chronic illness, particularly those who did not have cure, and their families and the whole situation.**

HA 1:

Aah… first of all I would like to thank madam for choosing our BHU as one of the samples for this research and on behalf of Ura BHU I would like to welcome you to our BHU la. To introduce myself I am Sonam C. and I graduated in 2007 (from RHIS – Royal Institute of Health Sciences) and I have been in service for the last twelve years. In the last twelve years of my service I have given aah…palliative care to at least few patients that I have come across and of course in Ura I have not given to a single one but in my previous place of stay I had to care for patients like Ca stomach and all these type of patients and those who were suffering from stroke and who had paralysis where we had to give wound dressing services and those who cannot take orally we also had to give IV fluids. And for the paralysis patients we had to visit quite often to change their positions to prevent bedsores. So this is my introduction.

**That’s good. So you haven’t had opportunity yet in Ura because I just knew that you have arrived here just two months ago. Is that right?**

HA 1:

Yes madam, I have been here for just two months now and till now I haven’t come across any cases but I think from now onwards there will be few cases. We do anticipate.

**Ok. Thank you.**

HA 2:

Aah… Thank you madam. Aah… to introduce myself my name is Deepen. S. and well, till today in Ura I haven’t met any aah…I haven’t received any terminal patients till today. I also have been here only for the last two months. We were both posted here at the same time. But then when I was in Lunana (Lunana community lies in the extreme northwest of the country and is located at an altitude of 3400m above sea level), that was my first posting, there I found many terminally ill patients whereby I had to give palliative care. Aah.. when it comes to giving palliative care in Lunana it was totally a different scenario. There we had to travel (walk) for around four to five hours to reach their place and we had to give all the over the counter medications and like for example a simple paracetamol, even to give that paracetamol we just have to travel on foot for four to five hours and visit their places and at the most what we used to do was advise the attendant as to what to do, what should have been done and what they have to do in terms of like emergencies and also on how to make life more better. So it was really challenging in Lunana. And aah…

**Just to interrupt, was the family cooperative? Were they willing to help the patients? How was it? Did they just rely on the health workers or were they also participative in the care?**

HA 2:
When it comes to cooperation, people there are quite primitive. I should say that word primitive because what I found was when there was a bedridden patient, when patient could not move around and could not go to the toilet so what they used to do was take the patient I mean outside the house. They won’t care if it is snowing, or whether it is raining, they just pitch a tent and keep the patient outside.

**Really?**

HA 2:
Yes, that’s the scenario of the patient care out there.

**And who would take care of the patient? Does somebody nurse the patient outside or is the patient left alone?**

HA 2:
They just give them food

**Just give them food and how about the other care, pain management, elimination needs and other needs?**

HA 2:
Aah… what they were saying was like aah ..the patient is defecating, passing stool, urine and that’s giving smell in the house. So what they do is just take the patient outside and let him/her be there. Most of the time there will be elderly people.

**They must be the grand parents or their own parents.**

HA 2:
Yes.

**How many such cases did you witness?**

HA 2:
Aah… around five including a 26 year old girl.

**What was wrong with her?**

HA 2:

Aah.. she was found to be paralysed. She could not move. There was no one to take care of her and she was bedridden.

**As a health worker in such situation how did you feel and what could you do to help these patients?**

HA 2:

Well, in that place I could not do much. Just aah…as a health worker I just gave the basic medications, basic nursing care and more than that I just asked the *Kidu* officer (peoples’ welfare officer) I just asked him that there are such cases going on and people are so ignorant about it. They are just OK with those kind of things. I just gave a focus, a light on that subject, on that matter and the *Kidu* officer could do something about it.

**What was done? Did something improve?**

HA 2:

The *Kidu* officer directly asked the Royal Family and what the Royal Family did was give the support and even they gave the helicopter to evacuate the patient to Thimphu and gave the major interventions and major health check-up.

**But then, its very interesting, right? We never thought that such things were happening in Bhutan. So was it a usual trend that whenever a patient is bedridden and they were not able to take care of their elimination needs that this was the practice?**

HA 2:
Yes. And nobody bothers about it. Aah…Lunana is such a place that even if you break your bones on the way nobody is going to pick you up. There may be people passing by you but they will not care about you. What they would do is give you a few packets of biscuits and leave you there only. That’s the situation in Lunana.

**So when they do that to their own family members then it is least expected that they help strangers on the way, right? Interesting.**

How about you Sonam, did you witness anything like that in in places where you have worked so far?

HA 1:
Aah… I think what my colleague said is a typical scenario but the cases that I have come across were not that extreme. The family members were very cooperative till the last breath of their patients. They gave care to their patients and all that I encountered were normal because the patient parties were cooperative and from time to time we also had to visit them and they used to give optimum care in many areas like personal hygiene. The first case that I came across was a case of aah..Ca stomach. That was recent not even one year. I think it was five to six months back when I was in Yurung BHU (under Pamagatshel District in eastern Bhutan). There was a woman who was 60 plus years old. She was a chronic gastric ulcer patient and towards the end she was also alcoholic and she used to eat *doma* (betel nut) regularly. She was later diagnosed as a terminal Ca stomach and for her every after two days I had to visit their home. I had to drive for about 30 minutes.

**Did they used to call you or since you knew that there is this patient you used to go on your own?**

HA 1:
Aah…firstly that patient was referred by me to Mongar Hospital and she was diagnosed at Mongar Hospital. And since she was in advanced stage they could not do much and the patient was sent back to her home for palliative care and once they reached home the family just informed me that their patient is sent back home. After two days I visited her and assessed her and found that she could not eat food or even drink anything. She vomited each time she ate or drank. The patient’s family asked if I could give her some IV infusion. So I had to go there every after two days and stay there whole day monitoring the IV fluids and in a day I could give two to three bottles of IV fluids, change her position.

**Did she have bed sores?**

HA 1:
Yes, towards the end. I think after discharging from Mongar hospital she survived only for a month. And the terminal stage was very painful because the family members knew that she was dying and the patient was also informed of her diagnosis at Mongar Hospital and I think she was also very cooperative.

Interesting Sonam. You saw a typical case which required palliative care at end-of-life, right? Did the patient have pain?

HA 1:
Yes madam. During the end, towards the terminal stage she had severe pain.

**Could you give her some pain medications?**

HA 1:
Yes madam, we could of course give Injection Voveran (Diclofenac) sometimes and she could not take oral tablets. What we could do was when she had severe pain the hospital had given her one or two ampules of Injection Tramadol and I used to give her that but the relief was very short and after few days she died.

**So was her pain controlled with injections Voveran or Tramadol or you thought the injections did not manage the pain adequately? How was it?**

HA 1:
At the initial stage I think the injections were really helping her and she could at least rest for few hours but as the disease got advanced I think even the pain killers had lesser effect on her pain. She could get only one to two hours of sleep. At the end I think there those pain killers were of no use of because thy didn’y work at all.

**Have you heard of opioid analgesics like morphine?**

HA 1:

Yes madam.

**Do you have supply of morphine in the BHU?**

HA 1:
Actually we didn’t have supply but sometimes hospitals give Tramadol injections for acute cases but in our case we do not have morphine la.

**This patient was sent from Mongar, was there any such analgesics prescribed for that patient? What was the analgesic prescribed for that patient?**

HA 1:
Aah… she was prescribed with Tramadol injection for severe pain

**Not morphine, right?**

HA 1:
No, not morphine

**So when she had severe pain towards the end-of-life and those medications you had did not work, how did you feel?**

HA 1:

Aah… the analgesics that are available in our BHUs, since we do not have other injections, we only have Voveran injection but on the other side the paracetamol she could not take orally. Whenever she took orally she used to vomit everything.

**How did you feel at that time? Did you wish that you had better knowledge or better skills or better facilities or did you ever feel helpless when you could not manage her that severe pain? How did you feel?**

HA 1:
Aah… firstly I really felt pity on her ..*smiles*..because she had been diagnosed with such a disease at aah…60 plus age. She was not very old and at that time what I thought was since I could not do much to relieve her pain I thought at least if some kind of other stronger analgesics were available in our BHUs that could have relieved her pain. Since we do not have supply in our BHUs we could not do much la.

**Interesting. Both of you had interesting experiences, witnessed interesting cases in different contexts umm….As a health worker how do you think the situation would improve? You know like Deepen has witnessed patients at terminal stage are taken out of the house, by their own family members, irrespective of the weather condition and they are kept outside the house. Umm.. why do you think people did that and how would the situation improve? What do you say about it?**

HA 2:

Well, here I would like to compare the situation between here in Ura and patients in Lunana. What I found was that people here are quite exposed to the new things (modernization) whereas they are limited in Lunana. Limited to their way of thinking, their way of life, they are still primitive. What I would say is they are still in like 1980s. I mean their thinking is like in the 1980s.

**So do you mean they are even limited to love, limited to compassion?**

HA 2:

Yes madam. Aah.. for example, there were a group of guys watching this aah… pornography movie and even the women, the children and their father, mother all watching it together. And I found that quite strange. And I asked them ‘don’t you feel ashamed’ they said it is a natural thing and they said it has come from outside meaning that it has come from the west. So that is the level of their thinking.

**Aah… when you give health education, I am sure you must have done lots of health education on areas, may not be in palliative care, but health education on non-communicable diseases and so on. How receptive are they? Are they interested to receive any kind of education. And do you think it will help improve their thinking and their way of living?**

HA 2:

Of course education is improving their lives I should say. Till today or till my duration what I found was there was gradual improvement in their health. They were more health seeking. Their health seeking behaviour improved. When I was first posted there they used to consider even health workers like some kind of enemy. Then as I stayed there, knowing them, knowing their habits, I mean we also adapted to their way of life. They kind of accepted us. So in that way they were complying with our advice, health education and what I would say is out of 100% around 80% of them follow our advice but there are few pockets of people who do not want to change. May be that is due to political reasons, I don’t know. But then they are never changing. There are pockets of people who are strictly following their religion whereby they don’t even if they are going to die they won’t take our medicines because they believe that their local deities and our medicines do not get along. Instead they clash and result in patients dying. So that is their belief. So that can’t be changed.

**OK. But it is interesting that as you mentioned about 80% of the people are comply and cooperate. Do you think that when it comes to palliative care, with education of health workers on palliative care and then educating the community, do you think the quality of life of patients who are terminally ill will improve compared to what you have witnessed there in the past.**

HA 2:

Well, what I believe is that it will take a long time for that to happen. Long time and only the effort of the health workers is not going to work there. What I believe is high level advocacy involving the bigger personalities, or involving like the Je Khenpo (The Chief Abbot, who heads the Central Monastic Body in Bhutan), the Royal family members.

**So it is that hard to make a difference for people in Lunana and for the way they take care of their health and living, right?**

HA 2:

Yes. As a health worker that’s what I have experienced there.

**With your experiences of having of having taken care of patients who were terminally ill and were dying what were some of the main challenges at a BHU level in the community context. Aah…How difficult was it? How was it?**

HA 1:
Aah… first thing was the distance la. Many of the terminal cases did not stay at the BHUs. They are always taken to their homes and the family members provide care at home and sometimes patients’ homes are far away from the BHU where we have to walk for two to three hours and sometimes one to two hours and for that we need to travel frequently and we also have lots of work at the BHU. If the patient was at the BHU the time taken to travel to their home will be greatly reduced. Secondly, what I feel is if all the terminal case patients are admitted in the ward and kept in the hospital they could get regular care and follow up and especially for those who are living in the far flung area the distance is the main challenge I faced so far.

**What do you know about palliative care so far? What is your understanding on palliative care?**

HA 1:

Aah.. when it comes to palliative care of course I have not received any formal training or workshop but what I understand is palliative care is the care given to the terminal cases, terminally ill patients, to reduce their pain and to give comfort.

Interviewer: How about you Deepen?

Ha 2:

Aah… same as what my colleague said but adding to that palliative care is also to adjust pharmaceutical and nursing care and also emotional care. So emotional care to the patient and also the family as to how to tackle the problems, how to handle the situation. I believe all these ingredients make up palliative care.

**Yes, it is interesting that without any training or anything you do have a basic understanding of palliative care. Yes, palliative care is a holistic approach of care to patients who are diagnosed with a life-threatening or life-limiting illness or a terminal illness and their families to improve the quality of life for as long as the patient lives and even beyond. Once the patient dies the families do go through lots of, you know, grief and bereavement and lots of issues afterwards so palliative care also extends to grief and bereavement period. So palliative care as Deepen mentioned is not only for the physical pain but when a patient is diagnosed with a terminal illness they also go through lots of social pain, spiritual pain and they also go through lots of cultural issues you know. So palliative care is a holistic approach of care. So umm… today having understood what palliative care is, how do you think that you can make a difference? What would you need? May be will see patients here in your community, right? They may be diagnosed elsewhere in the bigger hospital but and many a times actually in palliative care patients, studies have found in other parts of the world that patients actually want to come back to the community to die at their home. They want to come back to the community and if we have palliative care services we can reach the service at home. So when you understand that what is your wish? What do you think would be important so that we render this care?**

HA 1:

Aah… Training on palliative care, because we have not received any formal training on palliative care, once we receive such care then we would know how to care for such patients on how to give emotional care, psychological support. But till now as we haven’t received any training on it we just give them the basic care. If we receive palliative care training looks like we can care for such patients even more.

**So you think that training on palliative care would make so much of difference in the approach of care?**

HA 2

I also believe that having such training and improving our knowledge would make a difference to the patients because we are giving the health care. So as well as aah…making the public aware that such care is available, that would make a difference. Not just us but they should also be knowing that such care is given and are available. That’s what I feel.

**Aah.. So when a patient is diagnosed at an advanced stage you know at an advanced stage of cancer or any other chronic illnesses where there is no prospect of cure and the patient wishes to come back to the community, you know, the role of Health Assistants in the community becomes very, very important to them, right? They look up to you for taking care of their pain issues and other nursing aspects like may be position changing or wound dressing. You can also involve the family members to help them actually. In places like Kerala in India where there is very good palliative care service the health workers educate the family members and the family members actually can do procedures like dressing you know, position changing, feeding and many other such procedures. Aah… in Ura BHU here, what are some of the cultural or spiritual aah…beliefs or values that people prioritises when there is a terminally ill patient in the family? Any idea? Or even in other places where you have worked?**

HA 2:

Aah… well.. last time I went to attend an emergency outcall.

**Was it here in Ura?**

HA 2:

Yes, and what I found out was that when I was about to give Diclofenac sodium to the patient, the Voveran injection, the patient party refused saying that injecting while they are sick is like directly going against their religion. They said in the local term ‘*Dhoen Kham*’ (evil spirits). When the local deity is doing some harm to the family.

**What was the problem with the patient?**

HA 2:

Patient was bedridden. The patient, I mean the patient actually expired few days later. The patient was suffering from cardiac disease.

So was it a chronic heart disease?

HA 2:

In that situation I just wanted to relieve the pain. At that time the patient was having urinary tract infection and it was giving an uncomfortable pain to her. So I just wanted to relieve that pain. But then they believe that injecting the medicine is against the religion and will kill the patient. So that was the situation and they didn’t let me inject the pain killer.

**So umm…the religion and cultural beliefs sometimes makes it difficult to treat the patient, right? Umm.. when there is a terminally ill patient in the community, do you think that as a health worker, as a health assistant in the community, do you think that you can actually improve the quality of life of those patients and families? Do you see your potential although at the moment you may not have adequate knowledge and skill, but if you receive some kind of workshops or trainings on palliative care do you think that you have a potential to improve the quality of life of those patients and families even at a BHU even as BHU staff?**

HA 1:

Aah… Actually to some extent after we get training and workshops since we will learn many things from there and to some extent I think we can help the terminally ill patients and give them some comfort. With training I am sure we can do much more than what we have been doing so far and that’s what I feel.

Interviewer:

Because umm…palliative care is actually nothing very complicated, you know. Umm.. any procedure that you do is already learned as a clinician during our Health Assistant training. But in palliative care our outlook is to improve the quality of life. So it is not always technology based. It is not only intellect but it is also so much to do with our compassion, with your heart and with two of you here in Ura BHU at the moment aah.. if, you know, if one of you or both of you get to attend palliative care workshop for a week or so do you think two of you will be able to manage or do you think you would need extra staff if you are to render palliative care service? What do you say about it?

HA 2:

For now I think we two are doing good and I think we can tackle that problem. I mean till today we have been caring for the elderly people. We are taking care of them, looking after them, following up on them. I think we won‘t need any extra people to be involved to provide palliative care. Provided we get some opportunities for training.

HA 1:

Yes madam, of course we will definitely be able to do that.

**Because if you have a palliative care training you will also understand about other essential palliative care drugs like, you know, opioid drugs like Morphine which is very cheap but very effective in most of the moderate to severe pain. So I could understand that you could not adequately manage pain in your past experiences mainly because you didn’t have knowledge and also you didn’t have drugs in stock. And the drugs were not in stock may be because HAs are not trained in giving such drugs, right? So if the system improves then you will be able to do that, right?**

HA 1:

Yes

**So in our brief discussion today you have understood that we are looking forward to introducing palliative care service in the country, to integrate palliative care service into the Bhutanese health care system and you know gradually bring palliative care to every health centre. That is the objective of this project. Having understood that, do you have any specific advice, suggestions or any comments to me for this project?**

Ha 2:

Well, today I am just hearing about this but then I would like to know more about it. I mean I would like to get some trainings and I like to know what its core values are, I mean, what and how it is going to work and how it will be helpful to us as well as to the community. So here what I believe is first and foremost is training us would be like improving the senses like improving our knowledge. What I believe is first and foremost the program should focus on improving the HAs because we are always the first one to .. what I believe is that we are the fingers of the health system. So making the fingers senseless would be a problem. So (…laughs)

**That’s interesting and I do agree. Because palliative care is a service which need not only be at the referral centres, you know. As I mentioned earlier, given an opportunity many of the dying patients would actually like to come back and die at home. So one of our aims is to train the community health workers to be able to give palliative care to those patients who are in the community. Because the patients and families should not regret that, you know, ‘We have come home but the pain is not adequately managed. At JDWNRH in Thimphu at least the pain was well managed’ you know what I am trying to say. So that is our objective. Our aim is while planning to bring palliative care to the community the first thing, even before creating awareness among the public, is to train the health workers. It may take three to five years but that is what the project aims.**

Is there anything else that you think we should discuss before we close? Anything from your experiences, you know, from where you have taken care of such patients and families? May be we might have missed to discuss certain things from your experiences. Is there anything?

HA 1:

There is nothing as such

HA 2:

In future when madam would be giving lectures on palliative care I should say that we should train on how to handle the emotional part of the palliative care. How to give emotional support. Like just saying ‘don’t worry, this is the way of life’ when you say that it is not going to work. That’s not going to work. So we need to understand whole lot of things here. Their side of the story, not just their emotional pain, but their psychological pain as well. Well I have been in my service for four years and I have tried my best but then still failed in terms of giving emotional support to the families. So I would like to know more on that as well.

**That’s a very important point. I think maybe I missed a very important point when I was explaining about what is palliative care. Yes, palliative care, because patients with advanced illness or chronic illness goes through different types of pain, physical pain, psychological, emotional and spiritual pain, so palliative care is a holist approach of care you know. Addressing each of this pain in the patient and that of the family members is crucial in palliative care. The training modules will be designed accordingly. It is very important that we all be able to help the person holistically and you have rightly pointed out Deepen. Thank you for this comment.**

**Anything else before we close?**

**Nothing? That’s it, right? So I think I have learnt so much from you from your experiences, the challenges you have had and the limitations you faced as you handled with terminally ill patients and their families. Thank you very much for sharing your experiences. I look forward that one day we will have palliative care service brought to the community of Ura.**

**Thank you very much**

HA 1:

Thank you very much madam

HA 2:

Thank you so much madam
