# Supplementary material for: Healthcare professionals’ views on how palliative care should be delivered in Bhutan: A qualitative study
Source: PLOS Glob Public Health. 2022 Dec 12;2(12):e0000775. doi: 10.1371/journal.pgph.0000775 (PMC10021767; doi:10.1371/journal.pgph.0000775)
Supplement: S26 Data — (DOCX) [file pgph.0000775.s027.docx]

**Field Note FGD with HCP in Ura BHU I**

Date: 25.5 2019

Ura BHU is about two hours’ drive from Bumthang town and the two health care professionals were informed over phone yesterday that I am visiting them for the research purpose and had provided a brief background of the research project. Both the health assistants were eager to participate in the research. I arrived at the BHU at around 10:30 AM this morning and since it is Saturday there were not many patients. They had cleared their OPD and were looking forward to my arrival.

On arrival I introduced myself again and provided with the participant information form and were given enough time to read it. The BHU also had traditional medicine unit but it was managed by the technician and there was no Drungtsho, the traditional physician. Since the technician is not included as a study participant I could recruit only the health assistants. Both of them wanted to participate for the survey and as well as for the discussion. Although in the proposal it is mentioned that only one health assistant will be interviewed however, in this BHU both of them expressed their interest to participate and so a focus group was conducted with the two of them.

Although both the HAs were quite new to Ura BHU, both have been there for the last two months, they both had interesting experiences from their past workplaces when it came to taking care of patients with advanced illnesses. They were both very keen to participate in the discussion and were both very participative. The discussion took 46 minutes 58 seconds. The discussion was conducted in the office of the HA and there was no disturbances or any interferences. The discussion was conducted in English, Dzongkha and at time the local dialect. The discussion went very well and it was satisfying. They were both very supportive for the project.
