# Supplementary material for: Healthcare professionals’ views on how palliative care should be delivered in Bhutan: A qualitative study
Source: PLOS Glob Public Health. 2022 Dec 12;2(12):e0000775. doi: 10.1371/journal.pgph.0000775 (PMC10021767; doi:10.1371/journal.pgph.0000775)
Supplement: Attachment — Submitted filename: Responses to reviewers.docx [file pgph.0000775.s028.docx]

Dear Catherine Kyobutungi,

Thank you for reviewing our paper. Please find below the responses to the comments from the editors and reviewers. The page numbers reflected in the response column are the page numbers in the revised manuscript with track changes.

| **Editor’s comments** | **Response** |
| --- | --- |
| Please address all comments from reviewers. | Thank you for this comment. We did our best to address all the comments from reviewers. |
| Please make use of the COREQ guideline (or equivalent) and attach a completed checklist: <https://www.equator-network.org/reporting-guidelines/coreq/> | Thank you for this comment and for the link to the checklist. We have completed and attached the COREQ guideline |
| The manuscript is quite long. Can some sections in the introduction and discussion be condensed? For example, information about Gross National Happiness or indeed disease burden is not directly relevant to the topic of the paper | Thank you for this comment. We have now deleted some information about Gross National Happiness, Page 3, Lines 67-71; and about the traditional medicine in Bhutan, Page 4, Lines 80-83. We also tried condensing where possible in the discussion too Lines 531, 538-539, 562-564. |
| Please consider adding a box to summarise some key recommendations based on your findings for how palliative care can be developed in Bhutan | Thank you for this comment. A box with recommendations is added in the manuscript, Page 31. |
| Please do not use the abbreviation 'PC' as this is non-standard (spell out palliative care in full) | Thank you for this comment. We have replaced ‘PC’ with ‘palliative care’ throughout the manuscript. |
| Reviewer #1 |  |
| Thank you for the opportunity to review this document. It is an original and significant study in the field of palliative care, in my opinion. | Thank you very much for this comment! |
| There is no such thing as a qualitative cross-sectional study. One of the quantitative designs is cross-sectional design. You could say: descriptive qualitative study only. | Thank you for this comment. We have changed it to ‘descriptive qualitative study’, Page 5, Line 102. |
| Table 1 might be moved to the results section | Thank you for this comment. We have moved Table 1 (now it is Table 2) to result section, Page 9-10. |
| I am uncertain as to why TDL collected quantitative and qualitative data (line 28, page 6) This is a qualitative study, so please keep that in mind. Even if the researcher is collecting quantitative data for another study or for the demographic information, I do not believe this is relevant to this study's reader. | Thank you for this comment. The statement ‘collecting quantitative and qualitative data’ mentioned now in page 7, Line 133, was to explain to the readers why data analysis could not be performed while collecting data and thus data saturation could not be achieved for this study. |
| Data analysis: please provide step-by-step instructions. | Thank you for this comment. We have added a step-by-step description of data analysis, Page 8, Lines 149-155 |
| Reviewer #2: |  |
| Thank you for the opportunity to review this important work. This is a lovely study and conducted and reported really well – congratulations. It will be really interesting to see your full program of research evolve – such an important program of work. I hope my comments below are helpful as you finalise this study for publication. | Thank you very much for this comment! |
| Abstract: This is well written and provides a good overview of the study – well done. Just a few small thoughts for your review: • I am not sure it is helpful to say palliative care is more necessary in one country over another to be honest – would suggest removing ‘where it is actually needed most’ and replace with ‘ despite evident need’ or something similar. Even in high-income countries there is an equal need for optimal palliative care etc. | Thank you for this comment.  We decided to remove the phrase ‘where it is actually needed most’. |
| Line 33 – rather than palliative care ‘beginning to get recognised’ – perhaps is ‘in its infancy’ or something like that? | Thank you for this comment. We have replaced ‘beginning to get recognised’ with ‘palliative care is an emerging concept’, Page 2, Line 33. |
| Line 34 ‘their’ country | Thank you for this comment. We have changed it to ‘in Bhutan’, Page 2, Line 35. |
| Line 44 ‘services’ | Thank you for this comment. We have added ‘s’ to service, Page 2, Line 44. |
| Introduction – from my perspective this provides a great introduction to your study – well written and interesting to read – well done. | Thank you very much for this comment! |
| Data collection – I think it would be helpful to include a table or textbox with your semi-structured question route so readers can see quickly the key elements of your discussion points (as many won’t access your transcripts in supplementary files etc). | Thank you for this comment. We have included the semi-structured questions in Table 1, Pages 7-8 |
| I understand your point in relation to data saturation – but I think it would be helpful to note something about sampling that confers confidence that you considered the numbers and representatives carefully to ensure adequate information overall? You may find some useful resources to support your thinking in the following? | Thank you for this comment. We have added a sentence explaining the sampling strategy and distribution of study sites as a rationale behind not being able to consider data saturation, Page 7, Pages 135-137 |
| Results Well written and easy to read. These are quite long but if they fit within the journal’s guidelines – all good. I found it a very interesting read! The quotes clearly articulate your points and support the themes noted. | Thank you very much for this comment. |
| Discussion Again – well written – thank you. I just wonder if it may be helpful to frame your results within the WHO Innovative Care for Chronic Conditions Framework – it seems to really fall within their key areas strongly. | Thank you for this comment. We agree that our points in the discussion does fall within the ‘WHO Innovative Care for Chronic Conditions Framework.’ However, since the findings are more consistent with the WHO public health strategies specific to palliative care, we decided to frame our discussion around it. |
| Line 471 – when you state ‘in several LMICs’ – could you be a little more specific – which LMICs and how might this be useful when considering the Bhutan context – what has worked well / what not so well etc? | Thank you for this comment. We have specified the countries, Page 26, Line 514, and added a sentence to elaborate further on how Bhutan can also achieve Line 515. |
| Line 480 – how is the Bhutanese government committed – what evidence can support this as ‘appears committed’ seems a little vague | Thank you for this comment. We have rephrased the sentence and included additional information to support the sentence, Page 26, Lines 522-525. |
| Your strengths / limitations and conclusion sections all make good sense for me. All the best as you finalise this work. | Thank you very much for this comment. |

Thank you very much.

Sincerely Yours,

Tara Devi Laabar

(Corresponding author)

Date: 31/08/2022
